# Scandium‐Catalyzed Highly Selective Deoxygenation of Alcohols by Using Hydrosilanes as Reductants

**DOI:** 10.1002/chem.202501596

**Published:** 2025-06-30

**Authors:** Ruqaya Buhaibeh, Neethu Thyagarajan, Guillaume Bousrez, Louis Monsigny, Thibault Cantat, Emmanuel Nicolas

**Affiliations:** ^1^ Université Paris‐Saclay CEA, CNRS, NIMBE Gif‐sur‐Yvette 91191 France

**Keywords:** alcohols, ketones, scandium, selective deoxygenation

## Abstract

Herein, we present a straightforward and efficient scandium‐based catalytic system that enables highly selective reduction of σ bonds (C─O) in the presence of more reactive π (C═O) or O─H bonds. Employing 5 mol% of Sc(OTf)_3_ as the catalyst, a diverse range of aryl and alkyl hydrosilanes proved to be highly effective in reducing secondary and tertiary alcohols to the corresponding alkanes. Furthermore, this protocol was extended to the deoxygenation of ketones and the hydrodehalogenation of alkyl halides.

## Graphical abstract

A new protocol for selective C─O bond cleavage using Sc(OTf)₃ as the catalyst in the presence of hydrosilanes as reductants is reported. Alcohols, ketones, and alkyl halides were efficiently reduced to the corresponding alkanes, with excellent regio‐ and chemoselectivity.

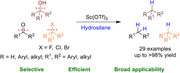

## 1 Introduction

Given the depletion of oil reserves and the urgency of combating climate change, enhancing energy efficiency and resource utilization in chemical processes is critical for sustainable development. Among these efforts, the targeted conversion of abundant functional groups found in nature is pivotal in improving organic synthesis efficiency.^[^ ^1^, ^2^, ^3^ ^]^ A crucial aspect lies in the deoxygenation reaction of alcohols, which facilitates the conversion of biomass‐derived alcohols into environmentally friendly fuels by cleaving C─O bonds.^[^ ^4^, ^5^ ^]^ Alcohol deoxygenation methods generally follow two main strategies. The first is a two‐step approach, exemplified by the Barton‐McCombie reaction, which involves converting the hydroxyl group into reactive xanthate intermediates, followed by a reduction step using a stoichiometric amount of a highly toxic tin hydride reagent. While effective, the toxicity and practicality issues of this method limit its broader application.^[^ ^6^, ^7^, ^8^, ^9^, ^10^, ^11^ ^]^ The second approach is direct deoxygenation, which faces significant challenges due to the high bond dissociation energy of C─O bonds. To address these challenges, various transition metals have been investigated, including Ti^[^ ^12^, ^13^, ^14^ ^]^ Pd,^[^ ^15^, ^16^ ^]^ Ir,^[^ ^17^, ^18^, ^19^ ^]^ Ru,^[^ ^20^, ^21^ ^]^ Mn,^[^ ^22^ ^]^ Co,^[^ ^23^ ^]^ and Ni,^[^ ^24^ ^]^ yet the need for relatively elevated temperatures, excessive bases, additives, and the high costs of noble metal catalysts has significantly limited the widespread application of these methods.

**Scheme 1:**
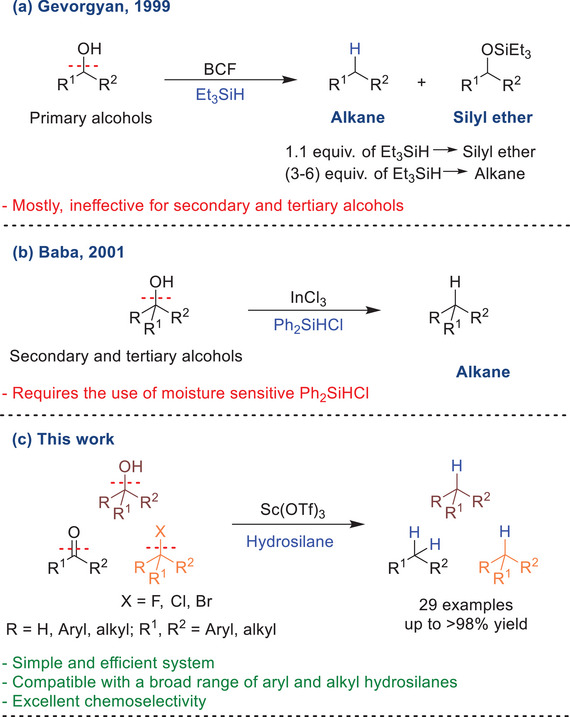
Lewis acid‐catalyzed deoxygenation of alcohols.

Since the mid‐1900s, Lewis acids have been recognized as effective agents for activating C─O bonds in deoxygenation reactions when used in conjunction with hydride sources.^[^ ^25^, ^26^, ^27^, ^28^, ^29^, ^30^, ^31^, ^32^ ^]^ Tris(pentafluorophenyl)borane (BCF), stands out as one of the most extensively utilized Lewis acids in such transformations, particularly when paired with hydrosilanes as reductants.^[^ ^33^ ^]^ This combination has proven effective in reducing a variety of carbonyl derivatives including ketones, aldehydes, and carboxylic acid derivatives.^[^ ^32^, ^34^, ^35^, ^36^, ^37^ ^]^ Gevorgyan et al.^[^ ^32^, ^35^ ^]^ demonstrated the successful reduction of alcohols to their corresponding alkanes using BCF as a catalyst with excess Et_3_SiH (Scheme 1a). Although this system efficiently deoxygenates primary alcohols, it is largely ineffective for secondary and tertiary alcohols unless they contain substituents capable of strongly stabilizing carbocations. Baba et al. explored the deoxygenation of ketones^[^ ^38^ ^]^ using InCl_3_ with Me_2_SiClH and subsequently expanded this method to alcohols,^[^ ^29^, ^30^ ^]^ successfully reducing secondary and tertiary alcohols to alkanes. However, the practical utility of this system was limited by the requirement for moisture‐sensitive chlorohydrosilanes (Scheme 1b).

In this work, we introduce a simple and efficient protocol for the deoxygenation of secondary and tertiary alcohols to their corresponding hydrocarbons, using Sc(OTf)_3_ as a catalyst and hydrosilanes as reductants (Scheme 1c). Additionally, we extended the system's reactivity to effectively deoxygenate ketones and promote the hydrodehalogenation of alkyl halides. Importantly, this method addresses the limitations of previous systems, providing a highly selective strategy for the reduction of σ bonds (C─O) in the presence of π (C═O) or O─H bonds.

## 2 Results and Discussion

We initiated our investigations on the catalytic activity of Sc(OTf)_3_ in the direct deoxygenation of alcohols, using 1‐phenylethanol as a model substrate (Table 1). Reaction of 1‐phenylethanol **1a** with 2.0 equiv. of Et_3_SiH in the presence of 10 mol% of Sc(OTf)_3_ in CD_2_Cl_2_ at 60 °C for 4 hours resulted in complete conversion (>98%) to the desired ethylbenzene **2a** (Table 1, entry 1). Lowering the amount of Et_3_SiH to 1.1 equiv. maintained the reaction's efficiency, achieving full conversion with 98% yield of **2a** and only a minor formation (2%) of the ether product **3a**, (Table 1, entry 2). However, reducing the reaction time to 1 hour under identical conditions significantly lowered the yield of ethylbenzene **2a** to 6%, while increasing the formation of the ether product **3a** to 65% (Table 1, entry 3). Further, increasing the reaction temperature to 80 °C restored full conversion and produced 98% yield of **2a** within 1 hour (Table 1, entry 4). Reducing the reaction time further to 30 minutes under these conditions led to a moderate decrease in yield, with **2a** obtained in 75% yield and **3a** in 17% (Table 1, entry 5). Notably, lowering the catalyst loading from 10 mol% to 5 mol% at 80 °C for 1 hour maintained excellent conversion and product selectivity (Table 1, entry 6). However, a further reduction to 1 mol% resulted in a marked decrease in selectivity, yielding **2a** in 39% and **3a** in 52% (Table 1, entry 7). A control experiment without the catalyst confirmed the essential role of Sc(OTf)_3_, as no product formation was observed (Table 1, entry 8). Furthermore, running the reaction under air had no adverse effect on its efficacy, with **2a** obtained in 96% yield (Table 1, entry 9).

**Table 1:** Optimization of reaction conditions for the deoxygenation of 1‐phenylethanol 1a^[^ ^a^ ^]^

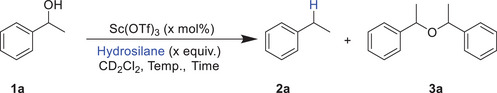							
Entry	Sc(OTf)_3_ (mol%)	Hydrosilane (equiv.)	Temp. (°C)	Time (h)	Conversion %^[^ ^b^ ^]^	2a %^[^ ^b^ ^]^	3a %^[^ ^b^ ^]^
1	Sc(OTf)_3_ (10)	Et_3_SiH (2)	60	4	>98	>98	0
2	Sc(OTf)_3_ (10)	Et_3_SiH (1.1)	60	4	>98	98	2
3	Sc(OTf)_3_ (10)	Et_3_SiH (1.1)	60	1	71	6	65
4	Sc(OTf)_3_ (10)	Et_3_SiH (1.1)	80	1	>98	98	2
5	Sc(OTf)_3_ (10)	Et_3_SiH (1.1)	80	0.5	94	75	17
6	Sc(OTf)_3_ (5)	Et_3_SiH (1.1)	80	1	>98	98	2
7	Sc(OTf)_3_ (1)	Et_3_SiH (1.1)	80	1	95	39	52
8	None	Et_3_SiH (1.1)	80	1	0	0	0
9^[^ ^c^ ^]^	Sc(OTf)_3_ (5)	Et_3_SiH (1.1)	80	1	>98	96	4
10	Sc(OTf)_3_ (5)	Et_2_SiH_2_ (1.1)	80	1	>98	>98	0
11	Sc(OTf)_3_ (5)	PhSiH_3_ (1.1)	80	1	>98	81	19
12	Sc(OTf)_3_ (5)	PhMe_2_SiH (1.1)	80	1	>98	95	6
13	Sc(OTf)_3_ (5)	Ph_2_SiH_2_ (1.1)	80	1	>98	89	11
14	Sc(OTf)_3_ (5)	PMHS (1.1)	80	1	>98	93	3
15	Sc(OTf)_3_ (5)	TMDS (1.1)	80	1	>98	>98	0
16	Sc(OTf)_3_ (5)	TMDS (1.1)	80	30	>98	>98	0
17	Sc(OTf)_3_ (5)	TMDS (0.5)	80	1	>98	59	40

^[a]^ General conditions: **1a** (0.1 mmol, 12 µL), Hydrosilane (x mmol, x µL), Sc(OTf)_3_ (x mol%), and CD_2_Cl_2_ (0.5 mL),

^[b]^ Conversions and yields are determined by ^1^H NMR with mesitylene as the internal standard,

^[c]^ Reaction was performed under air atmosphere.

Employing the optimized reaction conditions (Table 1, entry 6), we explored alternative catalysts, solvents, and hydrosilanes to identify the optimal system for deoxygenation. Screening a range of metal ion catalysts, (Table S1, S5, SI), revealed Sc(OTf)_3_ as the most effective, achieving 98% yield of the target product **2a**. Among the lanthanide triflate catalysts tested, Ce(OTf)_3_ exhibited moderate activity, delivering **2a** in 63% yield, alongside 36% of the ether product **3a**. In contrast, La(OTf)_3_ showed no catalytic activity, while other catalysts such as Sm(OTf)_3_, Eu(OTf)_3_, and Nd(OTf)_3_ predominantly produced the ether product **3a**, with only trace amounts of the ethylbenzene **2a** and the dehydrated product **4a**. Additionally, Yb(OTf)_3_ resulted in a mixture comprising 58% of the ether intermediate **3a** and 34% of styrene **4a**. Interestingly, Fe(OTf)_3_ which possesses higher Lewis acidity than Sc(OTf)_3_, furnished a complex mixture of three different products (**2a**, 33%; **3a**, 38%; and **4a**, 29%), showing reduced selectivity relative to Sc(OTf)_3_. Furthermore, when FeCl_3_ was tested, almost no catalytic activity was observed, producing only 6% of the ether product **3a**. The formation of the dehydrated product **4a** aligns with a previous study by Repo et al.,^[^ ^39^ ^]^ which demonstrated that alcohols can be converted into their corresponding alkenes using highly oxophilic and Lewis acidic metal triflates. Catalysts such as Hf(OTf)_4_ and Fe(OTf)_3_ were identified as highly efficient in such reaction, whereas Sc(OTf)_3_, with its comparatively lower acidity, was deemed inactive in that context. These results suggest that neither the strongest nor the weakest Lewis acids optimize the system's efficiency; rather, an appropriate balance of Lewis acidity is essential for identifying the ideal catalyst.

With the optimized catalyst in hand, we evaluated various solvents, finding that solvent selection was crucial for the reaction's success. Dichloromethane was uniquely effective, while solvents such as toluene, acetonitrile, and chloroform primarily formed the ether product **3a**, and tetrahydrofuran was entirely inactive (Table S2, S6, Supporting Information).

The effect of hydrosilane was also investigated. Interestingly, different aryl and alkylsilanes such as Et_3_SiH, Et_2_SiH_2_, tetramethyldisiloxane (TMDS), Ph_2_SiH_2_, Ph_3_SiH, and polymethylhydrosiloxane (PMHS), were all exhibited high efficiency, providing full conversions with excellent yields (>81%) (Table 1, entries 10–15). Moreover, the silylation of the alcohols was never observed, indicating a preferential activation of the C─O bond compared to the O─H bond in the C─O─H moiety.

**Scheme 2:**
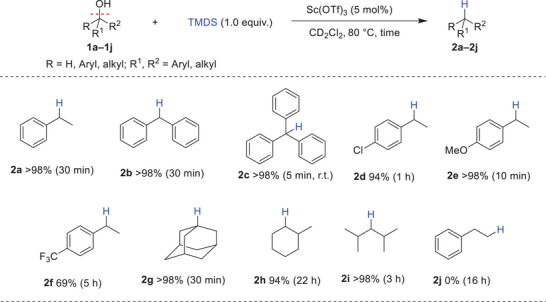
Substrate scope for the deoxygenation of alcohols. Reaction conditions : 1 (0.1 mmol), TMDS (0.1 mmol, 1.0 equiv.), Sc(OTf)_3_ (5 mol%), and CD_2_Cl_2_ (0.5 mL), Conversions and yields are determined by ^1^H NMR with mesitylene as the internal standard.

We selected TMDS for further studies as it is a readily available and cheap hydrosilane.^[^ ^40^ ^]^ Notably, the reaction time could be reduced to just 30 minutes without compromising efficiency (Table 1, entry 16). However, using only 0.5 equiv. of TMDS led to a decreased yield of the desired product **2a** (59%), accompanied by the formation of 40% of the ether product **3a** (Table 1, entry 17). Under the optimized conditions (TMDS 1.0 equiv., Sc(OTf)_3_ 5 mol%, 80 °C, in CD_2_Cl_2_), the substrate scope was investigated (Scheme 2). Secondary and tertiary benzylic alcohols (**1a**–**1d**) underwent smooth deoxygenation, yielding their corresponding alkanes in excellent yields (>94%). Substrate bearing electron‐donating group 1‐(4‐methoxyphenyl)ethan‐1‐ol **1e**, showed enhanced reactivity, delivering 1‐ethyl‐4‐methoxybenzene **2e** quantitatively within 10 minutes. In contrast, electron‐withdrawing groups slowed the reaction, as observed with 1‐(4‐(trifluoromethyl)phenyl)ethanol **1f**, that required 5 hours to achieve 59% yield of **2f**. Aliphatic alcohols **1g** and **1i** were efficiently converted to their respective products **2g** and **2i** in yields exceeding 98%. However, 2‐methylcyclohexanol **1h** required longer reaction time (22 hours) to achieve 94% of **2h**. Notably, primary alcohol **1j**, remained unreactive even after extending the reaction time to 16 hours. Furthermore, we showcased the synthetic utility of this approach by scaling the reaction to a 1 mmol scale. Diphenylmethane **2b** was isolated in 96% yield with Et_3_SiH and 89% yield when using TMDS (S7, SI).

### 2.1 Catalytic Deoxygenation of Ketones

**Scheme 3:**
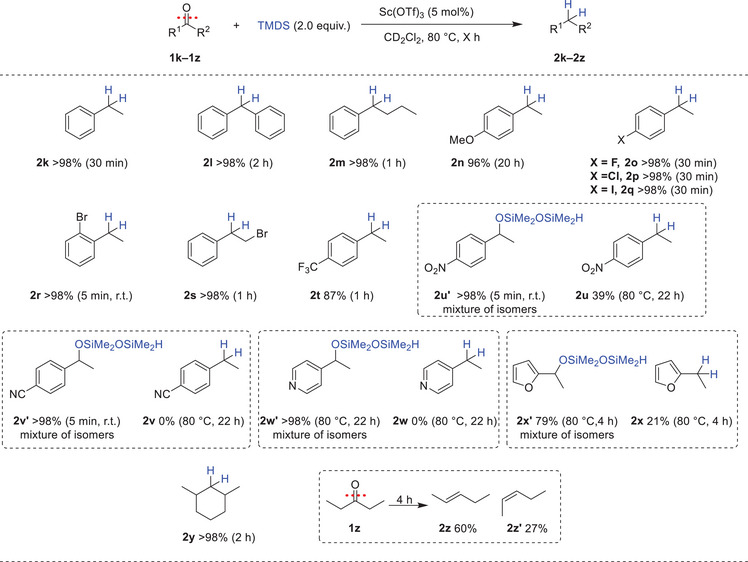
Substrate scope for the deoxygenation of ketones. Reaction conditions: 1 (0.1 mmol), TMDS (0.2 mmol, 2.0 equiv.), Sc(OTf)_3_ 5 mol%, and CD_2_Cl_2_ (0.5 mL), Conversions and yields are determined by ^1^H NMR with mesitylene as the internal standard.

**Scheme 4:**
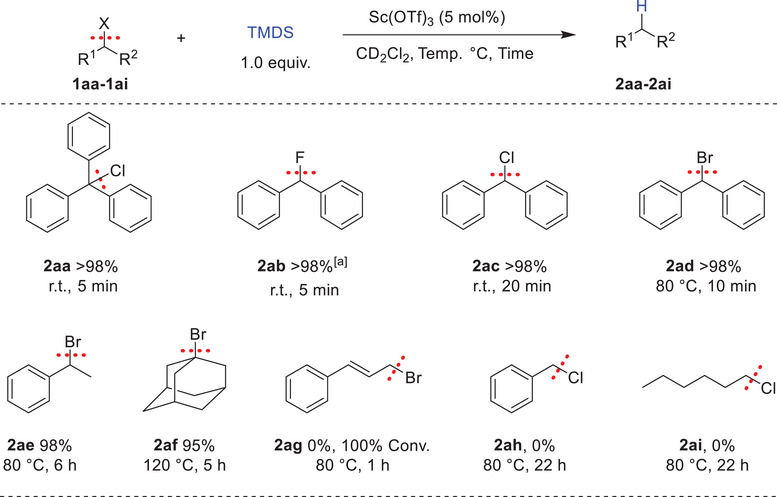
Substrate scope for the hydrodehalogenation of alkyl halides. Reaction conditions: 1 (0.1 mmol), TMDS (0.1 mmol, 1.0 equiv.), Sc(OTf)_3_ (5 mol%), and CD_2_Cl_2_ (0.5 mL), Conversions and yields are determined by ^1^H NMR with mesitylene as the internal standard. [a] 1.0 equiv. of Et_3_SiH was used instead of TMDS.

Catalytic reduction of ketones using hydrosilanes as hydride donors can yield diverse products. The predominant pathway, hydrosilylation reduction, leads to the formation of silyl ethers,^[^ ^41^, ^42^, ^43^, ^44^, ^45^, ^46^ ^]^ whereas a less common deoxygenation process generates the corresponding alkane.^[^ ^23^, ^47^, ^48^ ^]^ The Lewis acid‐catalyzed deoxygenation of alcohols approach has often been adapted for the deoxygenation of ketones. For instance, both the BCF/hydrosilane^[^ ^34^, ^49^, ^50^ ^]^ and InCl_3_/hydrochlorosilane^[^ ^38^ ^]^ systems have been employed in the defunctionalization of ketones to their corresponding methylene derivatives. Consequently, we extended this methodology to the deoxygenation of ketones (Scheme 3). Having optimized the reaction conditions (Table S3, S8, SI), Ketones (**1k**–**1z**) were treated with 5 mol% of Sc(OTf)_3_ and 2.0 equiv. of TMDS in CD_2_Cl_2_ at 80 °C. Under these conditions, acetophenone **1k**, benzophenone **1l**, and butyrophenone **1m** were quantitatively converted to their corresponding alkanes (**2k**–**2m**). Halo‐substituted aryl ketones (**1o**–**1r**) were successfully reduced to methylene compounds (**2o**–**2r**) without affecting the aryl halide functionalities. Similarly, the alkyl halide group in **1s** remained intact, affording (2‐bromoethyl)benzene **2s** in 98% yield. Interestingly, the electronic effects observed with ketones differed from those with alcohols. The electron‐rich *para*‐methoxyacetophenone **1n** required 20 hours to yield the desired product **2n** in 96% yield, whereas the electron‐deficient *para*‐(trifluoromethyl)acetophenone **1t** was deoxygenated in just 1 hour to afford **2t** in 87% yield. This inverse trend suggests distinct mechanistic pathways or rate‐determining steps between ketone and alcohol deoxygenation. In the case of ketones bearing potentially coordinating groups (**1u**–**1x**), the formation of the silyl ethers was dominated. *Para*‐nitroacetophenone **1u** was fully converted instantly at room temperature to the corresponding silyl ether **2u'** and yielded 39% of the alkane product **2u** after heating at 80 °C for 22 hours. In the same manner, the *para*‐cyano acetophenone **1v** and 4‐acetyl pyridine **1w** were also quantitatively converted to the corresponding silyl ethers (**1v'** and **1w'**) and no formation of the alkane products was observed even by continuing to heat the reaction mixture at 80 °C for 22 hours. 2‐acetyl furan **1x** yielded a mixture of the silyl ether **2x'** 79% and 21% of the deoxygenated product **2x** in 4 hours. Additionally, 2,6‐dimethylcyclohexan‐1‐one **1y** was smoothly converted into 1,3‐dimethylcyclohexane **2y** in excellent yield >98%. In contrast, the aliphatic ketone 3‐pentenone (**1z**) underwent a reductive dehydration process, yielding a mixture of alkene isomers (**2z**, trans 60%, and **2z'** cis 27%) rather than the expected alkane ((Scheme 4)).

### 2.2 Hydrodehalogenation of Alkyl Halides

Developing a catalytic system that is highly efficient for activating C─O bonds has prompted us to assess its reactivity with C–X derivatives, where X represents a halogen. Hydrodehalogenation, which replaces a halogen atom with a hydrogen, is a highly effective strategy for neutralizing hazardous organohalides. Methods employing boron Lewis acids in combination with hydrosilanes have been utilized for cleaving carbon–halogen bonds.^[^ ^51^, ^52^, ^53^, ^54^ ^]^ Thus the potential of our system for hydrodehalogenation of alkyl halides was evaluated. Using 5 mol% of Sc(OTf)_3_, and 1.0 equiv. of TMDS, secondary and tertiary organo(chlorides, fluorides, and bromides) (**1aa–1ad**) displayed excellent reactivity, achieving complete conversion with yields exceeding 98% (Scheme 4). Additionally, 1‐phenylbromoethane **1ae** and 1‐bromoadamantane **1af** were hydrodehalogenated in high yields (98% and 95%, respectively) under longer reaction times and elevated temperatures. However, cinnamyl bromide **1ag** was completely consumed but did not yield the expected product, instead forming unidentified byproducts. Meanwhile, benzyl chloride **1ah** and aliphatic 1‐chlorohexane **1ai** showed no conversion to the corresponding hydrocarbons, even under harsher reaction conditions.

### 2.3 Chemoselectivity

Having in hand a catalytic system capable of reducing various chemical bonds, including C═O, C─O, and C─X, we wished to explore its potential for other selective reductions, specifically investigating whether it could preferentially cleave σ bonds (C─O) in the presence of more reactive (C═O) double bonds.

**Scheme 5:**
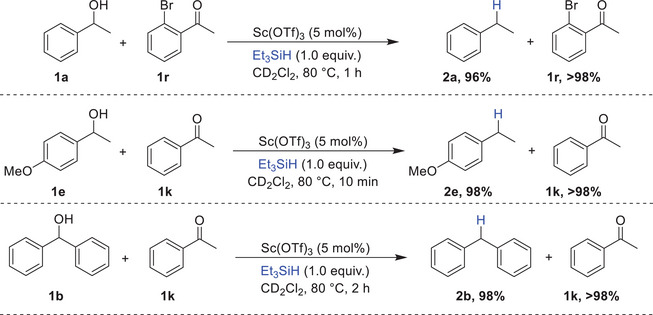
Chemoselective reduction of alcohols in the presence of ketones catalyzed by Sc(OTf)_3._

Our initial investigations focused on assessing the ability of the Sc(OTf)_3_/Et_3_SiH system to selectively reduce alcohols in the presence of ketones. Under optimized conditions and using only one equivalent of Et_3_SiH, mixing 1‐phenylethanol **1a** with 1‐(2‐bromophenyl) ethan‐1‐one **1r** resulted in the full conversion of alcohol **1a** into the corresponding alkane **2a** in an excellent yield of 96 %, while ketone **1r** was quantitatively recovered. Similarly, 1‐(4‐methoxyphenyl)ethan‐1‐ol **1e** and diphenylmethanol **1b** were selectively reduced to their respective alkanes, **2e** (98 %) and **2b** (98 %), in the presence of acetophenone **1k**, which remained fully untouched. (Scheme 5).

We then conducted a comparative study against the established BCF/Et_3_SiH and InCl_3_/Ph_2_ClSiH protocols (Table 2). Reactions involving 1‐phenylethanol **1a** in the presence of 1‐(2‐bromophenyl)ethan‐1‐one **1r** and 10 mol% of BCF, using either 1 or 2 equiv. of Et_3_SiH, yielded a complex mixture of silyl ether products **2a′** and **2r′** along with ether products **3a** and **3a′**. However, only 5% yield of the desired alkane **2a** was obtained when 2 equiv. of Et_3_SiH were used. A similar lack of selectivity was observed with the InCl_3_/ Ph_2_ClSiH system. With 1 equiv. of Ph_2_ClSiH, 1‐phenylethanol **1a** was converted to the silyl ether product **2a″** in 75% yield, with only 10% of the desired alkane **2a**, while 1‐(2‐bromophenyl)ethan‐1‐one **1r** remained unaffected. Increasing to 2 equiv. of Ph_2_ClSiH led to complete conversion of **1a** to ethylbenzene (**2a**, 69%) accompanied by unidentified byproducts, while ketone **1r** was fully converted into its silyl ether **2r″** (89%), with a minor formation of alkane **2r** (11%).

**Table 2:** Chemoselectivity studies (Alcohols versus Ketones), with Sc(OTf)_3_/Et_3_SiH, BCF/Et_3_SiH, and InCl_3_/Ph_2_ClSiH systems.

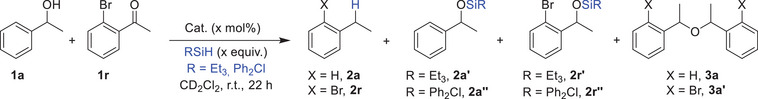						
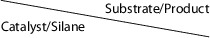	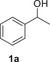	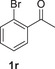	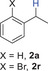	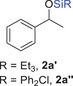	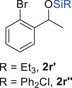	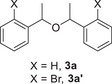
Sc(OTf)_3_ (5 mol%) Et_3_SiH (1.0 equiv.)^[^ ^a^ ^]^	‐	>98%	**2a** (96%)	‐	‐	‐
BCF (10 mol%) Et_3_SiH (1.0 equiv.)	‐	56%	‐	**2a′** (64%)	**2r′** (36%)	**3a** (36%) **3a′** (8%)
BCF (10 mol%) Et_3_SiH (2.0 equiv.)	‐	20%	**2a** (5%)	**2a′** (25%)	**2r′** (50%)	**3a** (69%) **3a′** (30%)
InCl_3_ (10 mol%) Ph_2_ClSiH (1.0 equiv.)	‐	>98%	**2a** (10%)	**2a″** (75%)	‐	**3a** (15%)
InCl_3_ (10 mol%) Ph_2_ClSiH (2.0 equiv.)	‐	‐	**2a** (69%) **2r** (11%)	‐	**2r″** (89%)	‐

^[a]^ The reaction mixture was heated for 1 hour at 80 °C.

These findings highlight the high chemoselectivity of our system, which prioritizes σ bonds C─O bond cleavage over π(C═O) or O─H bonds, with the use of only one equivalent of hydrosilane. This is in contrast to existing catalytic systems such as BCF/hydrosilane or InCl_3_/hydrochlorosilane, who necessitate the formation of silyl ether intermediates, with BCF generating H_2_^[^ ^32^, ^55^ ^]^ and InCl_3_ producing HCl^[^ ^30^ ^]^ as by‐products, thereby requiring an excess of hydrosilane to achieve comparable selectivities. These efficiency and selectivity make it a valuable tool for targeted reductions in synthetic chemistry.

Leveraging the inactivity of our system toward primary alcohols, we sought to explore the possibility of selectively reducing secondary alcohols in the presence of primary alcohols. Such regioselective deoxygenation represents a critical transformation in the valorization of alcohol‐rich feedstocks for high‐value chemical synthesis.^[^ ^56^, ^57^, ^58^ ^]^ Morandi and co‐workers reported a catalytic strategy for the regioselective deoxygenation of terminal 1,2‐diols at the primary position, enabling the synthesis of 2‐alkanols, using BCF as a catalyst and a combination of Ph_2_SiH_2_ and Et_3_SiH as reductants.^[^ ^59^ ^]^

**Scheme 6:**
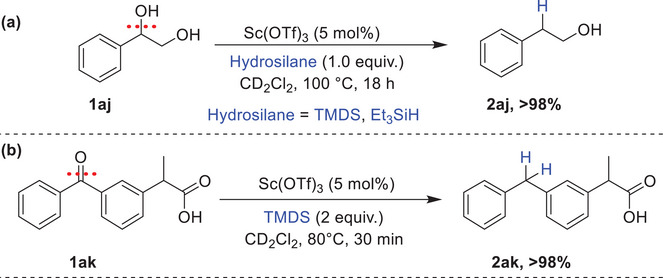
Regioselective a) and chemoselective b) deoxygenation catalyzed by Sc(OTf)_3._

Gratifyingly, reacting 1 equiv. of either TMDS or Et₃SiH with 1‐phenyl‐1,2‐ethanediol (**1aj**) resulted in highly regioselective monodeoxygenation at the secondary alcohol position, and affording the desired product **2aj** in an excellent 98% yield after heating the reaction mixture at 100 °C for 24 hours (Scheme 6a). Additionally, ketoprofen **1ak** underwent selective and efficient reduction using 2 equiv. of TMDS under mild conditions (80 °C for 30 minutes), leaving the carboxylic acid functionality unaltered (Scheme 6b). This selective methodology highlights its broad utility and potential for application in complex molecular transformations.

### 2.4 Mechanism of the Reaction

**Scheme 7:**
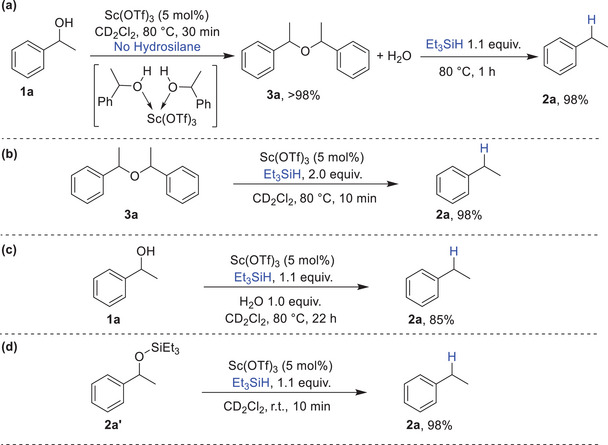
Control experiments for alcohol deoxygenation. a) sequential reaction, b) ether deoxygenation and cleavage, c) deoxygenation in the presence of water, d) deoxygenation of the silylated alcohol.

**Scheme 8:**
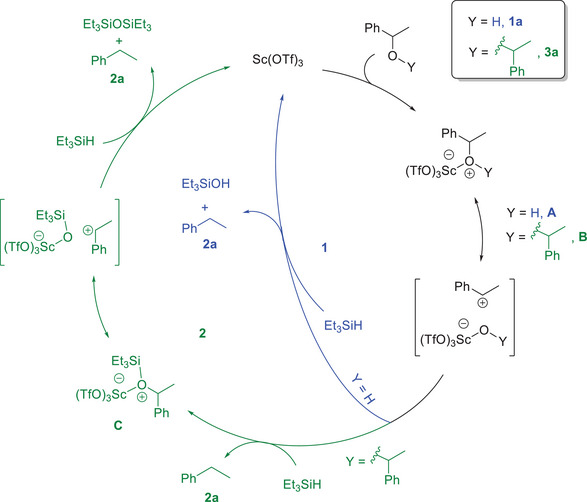
Proposed mechanism for Sc(OTf)_3_ catalyzed deoxygenation of alcohol.

To gain insights into the mechanism of the deoxygenation of alcohols, a series of control experiments were conducted, as illustrated in Scheme 7. In the absence of an hydrosilane, the reaction of 1‐phenylethanol **1a** with 5 mol% of Sc(OTf)_3_ resulted in complete conversion to the ether product **3a**, accompanied by the generation of water. Upon addition of 1.1 equiv. of Et_3_SiH to the same reaction mixture, **3a** was fully converted into the alkylated product **2a**. Such a dehydrative etherification of alcohols has been reported in the presence of various Lewis acids.^[^ ^60^, ^61^, ^62^, ^63^, ^64^, ^65^, ^66^, ^67^ ^]^ Interestingly, upon addition of 1 equiv. of water under optimal reaction conditions, 85% of ethylbenzene **2a** was obtained after 22 hours at 80 °C, indicating the scandium stability in aqueous environments, although the reaction was slowed. This behavior contrasts with that of BCF, which tends to form hydroxide adducts in the presence of water, thereby destroying its Lewis acidity and halting catalytic activity.^[^ ^68^ ^]^ Additionally, when we started the reaction with the ether intermediate **3a** in the absence of water, it was fully converted to the desired product **2a** within just 10 minutes at 80 °C. To investigate the hypothesized involvement of a silylated alcohol intermediate **C** (Scheme 8), the reaction was initiated with the silyl ether **2a′** which was fully converted to the desired product **2a** within 10 minutes at room temperature.

Based on these experimental findings, we propose a mechanism for the scandium‐catalyzed deoxygenation of alcohols as illustrated in Scheme 8. The observed lack of reduction in primary alcohols and the enhanced reaction efficiency with electron‐rich substrates suggest that the reaction proceeds through an intermediate that behaves like a carbocation. The proposed mechanism initiates with either 1‐phenylethanol **1a** or the ether intermediate **3a**, which, in the presence of Sc(OTf)_3_, could form an oxonium complex (**A** or **B**). These intermediates subsequently dissociate to generate the corresponding carbocation. In pathway **1**, the carbocation undergoes reduction by hydrosilane, yielding the hydrocarbon product **2a**, silanol, and regenerating the scandium catalyst. Similarly, in pathway **2**, the addition of Et_3_SiH induces hydride transfer to the carbocation, producing the alkane product **2a** alongside an oxonium complex **C**. The oxonium species **C** can dissociate again into a carbocation, which reacts with another molecule of hydrosilane to afford the desired product **2a** and hexaethyldisiloxane, then latter was isolated and confirmed via GC/MS analysis, while restoring the scandium catalyst to complete the catalytic cycle.

In the case of hydrodehalogenation, we propose that the mechanism proceeds via a pathway analogous to that of alcohol deoxygenation. This involves the generation of a carbocation intermediate through halide abstraction, followed by hydride transfer from the hydrosilane to the carbocation, ultimately yielding the desired product along with the corresponding halosilane byproduct (S22‐S26, Supporting Information). In contrast, the deoxygenation of ketones is well established to proceed via the formation of a silyl ether intermediate, which undergoes further reduction in the presence of an additional equivalent of hydrosilane.^[^ ^42^, ^50^ ^]^

## 3 Conclusion

We developed a highly efficient and simple catalytic system that exhibit excellent chemoselectivity, preferentially reducing σ bonds (C─O) over more reactive π (C═O) or O─H bonds, utilizing only one equivalent of hydrosilane. This approach enabled us to reduce alcohols in the presence of ketones, to monodeoxygenate secondary alcohols in a regioselective manner and in the presence of primary alcohol, and to selectively reduce a ketone in the presence of acid‐functional group. Additionally, secondary and tertiary alcohols were effectively transformed into their corresponding hydrocarbons, with primary alcohols remaining inert, unlike BCF system, which in general selectively deoxygenates primary alcohols. Furthermore, all hydrosilanes tested in this study performed effectively in the deoxygenation process, setting this method apart from other catalytic systems that often require specific types of hydrosilanes. Beyond alcohol deoxygenation, the system also showed high efficiency in the deoxygenation of ketones and the hydrodehalogenation of alkyl halides, highlighting its versatility and broad applicability.

## 4 Experimental Section

### General Procedure for the deoxygenation of alcohols

A 2.5 mL J. Young NMR tube in a glovebox was charged with alcohol (0.1 mmol), TMDS (1.0 mmol, 1.0 equiv.), Sc(OTf)_3_ (5 mol%), CD_2_Cl_2_ (0.5 mL), and mesitylene (5 µL) as internal standard. The tube was sealed and brought out of the glovebox, and the mixture was then stirred at 80 °C for the required time. Yields were determined by ^1^H NMR integration versus mesitylene.

### General procedure for the deoxygenation of ketones

In a 2.5 mL *J*. Young NMR tube in a glovebox, ketone 1 (0.1 mmol, 1.0 equiv.) and TMDS (0.2 mmol, 2.0 equiv.), and mesitylene (5 µL) were added to a solution of Sc(OTf)_3_ (5 mol%) in deuterated dichloromethane (0.5 mL). The tube was sealed and brought out of the glove box, and the solution was then heated at 80 °C for the required time. The reaction progress was monitored by ^1^H NMR spectroscopy. Yields were determined by ^1^H NMR integration versus mesitylene as an internal standard.

## Supporting Information

The authors have cited additional references within the Supporting Information.^[^ ^62^, ^69^, ^70^, ^71^, ^72^, ^73^, ^74^, ^75^, ^76^, ^77^, ^78^, ^79^, ^80^, ^81^, ^82^, ^83^, ^84^, ^85^, ^86^, ^87^ ^]^

## Conflict of Interests

The authors declare no conflict of interest.

## Supporting information



Supporting Information

## Data Availability

The data that support the findings of this study are available in the supplementary material of this article.
