# Supplementary material for: Scandium‐Catalyzed Highly Selective Deoxygenation of Alcohols by Using Hydrosilanes as Reductants
Source: Chemistry. 2025 Jun 30;31(40):e202501596. doi: 10.1002/chem.202501596 (PMC12272014; doi:10.1002/chem.202501596)
Supplement: Supplementary file 1 — Supporting Information [file CHEM-31-e202501596-s001.pdf]

Supplementary Information for

Scandium-Catalyzed Highly Selective Deoxygenation of Alcohols by Using Hydrosilanes as Reductants

Ruqaya Buhaibeh, Neethu Thyagarajan, Guillaume Bousrez, Louis Monsigny, Thibault Cantat, Emmanuel Nicolas*

Université Paris-Saclay, CEA, CNRS, NIMBE, 91191 Gif-sur-Yvette, France.

E-mail: emmanuel.nicolas@cea.fr

Table of Contents

1 General considerations S4
2 Deoxygenation of alcohols S5
2.1 General procedure : Optimization of the reaction parameters S5
2.2 General procedure : Catalyst screening S5
2.3 General procedure : Solvent screening S5
2.4 General procedure : Hydrosilane screening S6
2.5 General Procedure for the deoxygenation of alcohols S6
2.6 Procedure for 1 mmol scale of deoxygenation reaction using Et_3SiH S7
2.7 Procedure for 1 mmol scale of deoxygenation reaction using TMDS S7
3 Deoxygenation of ketones S8
3.1 General procedure : Optimization of the reaction parameters S8
3.2 General procedure for the deoxygenation of ketones S8
4 Hydrodehalogenation of alkyl halides S9
4.1 General procedure for the hydrodehalogenation of alkyl halides using TMDS S9
4.2 General procedure for the hydrodehalogenation of alkyl halides using E_3SiH S9
5 Chemoselectivity S10
5.1 General procedure for the chemoselectivity study: alcohols *vs* ketones S10
5.2 General procedure for the chemoselectivity study: BCF/Et_3SiH system S12
5.3 General procedure for the chemoselectivity study: $InCl_3$/Ph_2ClSiH system S14
5.4 Regioselective deoxygenation of internal 1,2-diol (1aj) at the secondary positionS16
5.5 Chemoselectivite deoxygenation of ketoprofen 1ak S17
6 Mechanistic Study S18
6.1 Control experiments for alcohol deoxygenation S18
6.1.1 Reaction in the absence of hydrosilane S18
6.1.2 Preparation of (oxybis(ethane-1,1-diyl))dibenzene 3a S19
6.1.3 Reaction starting from the ether 3a S19
6.1.4 Performing the reaction in the presence of H_2O S20
6.1.5 Reaction starting from the silyl ether 2a′ S21
6.2 Formation of halosilane: hydrodehalogenation of alkyl halides 1ab–1ad using E_3SiH
7 Characterization of products S27
7.1 Alkanes obtained by alcohols deoxygenation S27
7.1.1 ^1H and ^{13}C NMR of the isolated diphenylmethane (2b)[7] S28
7.1.2 ^1H and ^{13}C NMR of the isolated (oxybis(ethane-1,1-diyl))dibenzene 3a[12] S29
7.2 Alkanes obtained by ketones deoxygenation S29

7.3 Alkanes obtained by Hydrodehalogenation of alkyl halides using TMDS S31

7.4 Alkanes obtained by Hydrodehalogenation of alkyl halides 1ab–1ad using Et_3SiH S32

7.5 NMR data for the regio-chemoselectivite deoxygenation of 1aj and 1ak S33

7.6 Silyl ethers obtained by hydrosilylation of ketones (1u, 1v, and 1w) S33

8 References S35

9 Spectra S36

9.1 ^1H and ^{13}C NMR Spectra of alkanes obtained by alcohols deoxygenation S36

9.1.1 ^1H and ^{13}C NMR spectra of the isolated diphenylmethane (2b) using Et_3SiH as hydrosilane S46

9.1.2 ^1H and ^{13}C NMR spectra of the isolated diphenylmethane (2b) using TMDS as hydrosilane S47

9.1.3 ^1H and ^{13}C NMR spectra of the isolated hexaethyldisiloxane........................... S48

9.1.4 ^1H and ^{13}C NMR spectra of the isolated (oxybis(ethane-1,1-diyl))dibenzene 3a S49

9.2 ^1H and ^{13}C NMR Spectra of alkanes obtained by Ketones deoxygenation............ S50

9.3 ^1H and ^{13}C NMR Spectra of alkanes obtained by Hydrodehalogenation of alkyl halides using TMDS S63

9.4 ^1H and ^{13}C NMR Spectra of alkanes obtained by Hydrodehalogenation of alkyl halides 1ab–1ad using Et_3SiH... S68

9.5 ^1H and ^{13}C NMR Spectra of 2aj and 2ak... S71

9.6 ^1H and ^{13}C NMR Spectra of silyl ethers.. S73

1 General considerations

Unless otherwise stated, all reactions were performed in a recirculating *mBraun LabMaster DP* inert atmosphere (Ar) glove box. NMR spectra were recorded on a *Bruker Avance Neo* 400 MHz spectrometer. Chemical shifts were reported as ppm downfield from residual solvent peaks. Deuterated solvents were dried and stored under molecular sieves. 4 Å molecular sieves (Sigma-Aldrich) were dried under dynamic vacuum at 250 °C for 48 h prior to use. Analytical thin layer chromatography was performed on silica gel 60 F254 TLC plates (Merck). Visualization was achieved by exposure to ultraviolet light (254 nm). Purification: Flash chromatography was carried out with SiO_2 (particle size 20-60 µm). Reagents were purchased from commercial suppliers, stored in the glovebox, and used as received. 1-(4-Methoxyphenyl)ethanol **1e**[1], organohalides **1ab**,[2] **1ac**,[1] **1ad**,[3] and triethyl(1-phenylethoxy)silane **2a'**[4] were prepared according to literature procedures. All other reagents were purchased from Sigma Aldrich and used without further purification.

2 Deoxygenation of alcohols

2.1 General procedure : Optimization of the reaction parameters

A 2.5 mL J. Young NMR tube in a glovebox was charged with 1-phenylethanol **1a** (12 µL, 0.1 mmol), Et_3SiH (x equiv.), $Sc(OTf)_3$ (x mol%), CD_2Cl_2 (0.5 mL), and mesitylene (5 µL) as internal standard. The tube was sealed, brought out of the glove box, and the mixture was then stirred at the specified temperature for the required time. Yields were determined by ^1H NMR integration versus mesitylene.

2.2 General procedure : Catalyst screening

A 2.5 mL J. Young NMR tube in a glovebox was charged with 1-phenylethanol **1a** (12 µL, 0.1 mmol), Et_3SiH (12.7 mg, 1.1 mmol, 1.1 equiv.), catalyst (5 mol%), CD_2Cl_2 (0.5 mL), and mesitylene (5 µL) as internal standard. The tube was sealed, brought out of the glove box, and the mixture was then stirred at 80 °C for 1 h. Yields were determined by ^1H NMR integration versus mesitylene.

Table S1. Catalyst optimization for the deoxygenation of 1-phenylethanol ***1a***

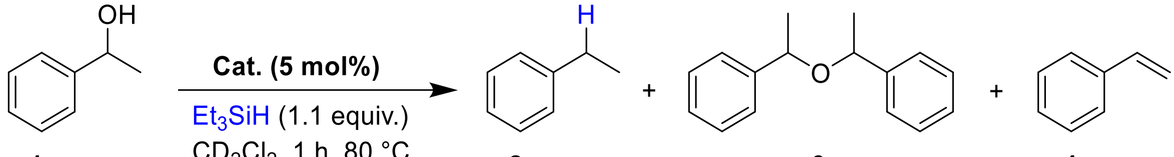

Entry	Cat. 5 mol%	Conversion %	NMR yield of 2a %	NMR yield of 3a %	NMR yield of 4a %
1	$Sc(OTf)_3$	>98	98	2	0
2	$La(OTf)_3$	0	0	0	0
3	$Ce(OTf)_3$	>98	63	36	1
4	$Sm(OTf)_3$	26	0	23	3
5	$Eu(OTf)_3$	61	5	53	3
6	$Yb(OTf)_3$	97	5	58	34
7	$Nd(OTf)_3$	83	5	75	3
8	$Fe(OTf)_3$	>98	33	38	29
9	$FeCl_3$	6	0	6	0

2.3 General procedure : Solvent screening

A 2.5 mL J. Young NMR tube in a glovebox was charged with 1-phenylethanol **1a** (12 µL, 0.1 mmol), Et_3SiH (12.7 mg, 1.1 mmol, 1.1 equiv.), $Sc(OTf)_3$ (5 mol%), solvent (0.5 mL), and mesitylene (5 µL) as internal standard. The tube was sealed, brought out of the glove box, and

the mixture was then stirred at 80 °C for 1 h. Yields were determined by ^{1}H NMR integration versus mesitylene.

*Table S2. Solvent optimization for the deoxygenation of 1-phenylethanol **1a***

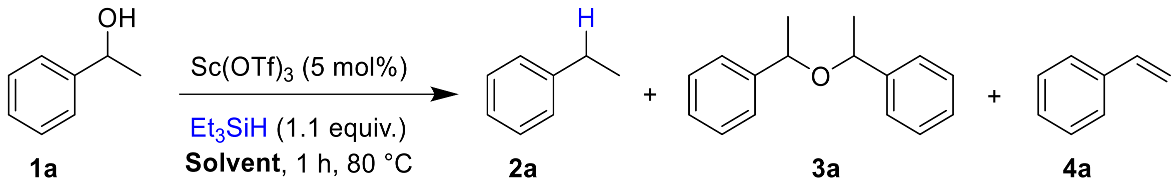

**Entry**	**Solvent**	**Conversion %**	**NMR yield of 2a %**	**NMR yield of 3a %**	**NMR yield of 4a %**
1	THF*d*8	6	0	6	0
2	Tol *d*8	44	2	42	0
3	CD_3CN	81	3	59	3
4	$CDCl_3$	88	6	80	3

2.4 General procedure : Hydrosilane screening

A 2.5 mL J. Young NMR tube in a glovebox was charged with 1-phenylethanol **1a** (12 µL, 0.1 mmol), hydrosilane (1.1 mmol, 1.1 equiv.), $Sc(OTf)_3$ (5 mol%), CD_2Cl_2 (0.5 mL), and mesitylene (5 µL) as internal standard. The tube was sealed, brought out of the glove box, and the mixture was then stirred at 80 °C for 1 h. Yields were determined by ^{1}H NMR integration versus mesitylene.

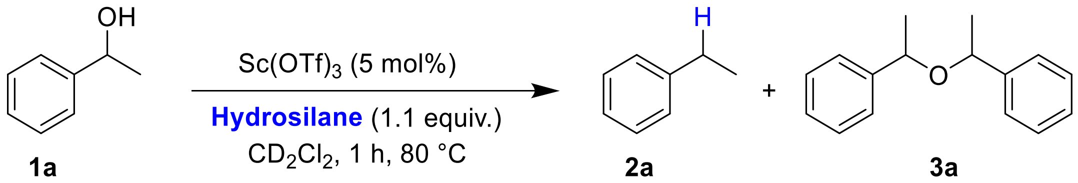

2.5 General Procedure for the deoxygenation of alcohols

A 2.5 mL J. Young NMR tube in a glovebox was charged with alcohol (0.1 mmol), TMDS (1.0 mmol, 1.0 equiv.), $Sc(OTf)_3$ (5 mol%), CD_2Cl_2 (0.5 mL), and mesitylene (5 µL) as internal standard. The tube was sealed and brought out of the glovebox, and the mixture was then stirred at 80 °C for the required time. Yields were determined by ^{1}H NMR integration versus mesitylene.

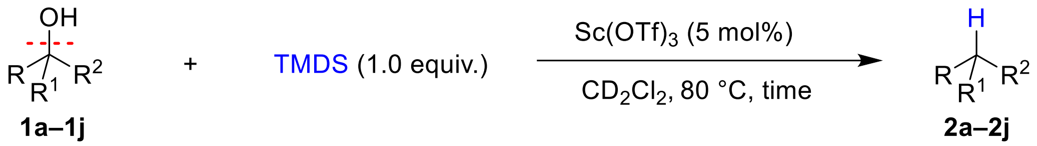

R = H, Aryl, alkyl; R^1, R^2 = Aryl, alkyl

2.6 Procedure for 1 mmol scale of deoxygenation reaction using Et_3SiH

In a glovebox, a 20 mL Schlenk tube was charged with **1a** (1 mmol, 184 mg), Et_3SiH (176 µL), $Sc(OTf)_3$ (25 mg, 5 mol%), and non-deuterated dichloromethane (5 mL). The mixture was then stirred for 2 h at 80 °C. The solvent was evaporated and the crude residue was purified by column chromatography (SiO_2, mixture of cyclohexane/ethyl acetate as eluent 9:1) to afford the desired product as a colorless liquid (161 mg, 96%).

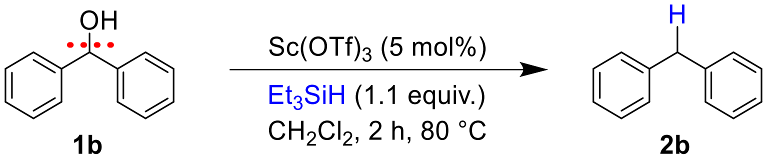

2.7 Procedure for 1 mmol scale of deoxygenation reaction using TMDS

In a glovebox, a 20 mL Schlenk tube was charged with **1a** (1 mmol, 184 mg), TMDS (190 µL), $Sc(OTf)_3$ (25 mg, 5 mol%), and non-deuterated dichloromethane (5 mL). The mixture was then stirred for 1 h at 80 °C. The solvent was evaporated and the crude residue was purified by column chromatography (SiO_2, mixture of cyclohexane/ethyl acetate as eluent 9:1) to afford the desired product as a colorless liquid (150 mg, 89%).

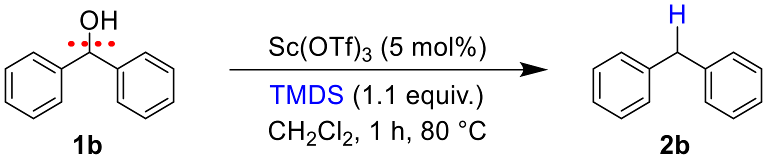

3 Deoxygenation of ketones

3.1 General procedure : Optimization of the reaction parameters

A 2.5 mL J. Young NMR tube in a glovebox was charged with acetophenone **1k** (11.7 µL, 0.1 mmol), hydrosilane (x equiv.), $Sc(OTf)_3$ (x mol%), CD_2Cl_2 (0.5 mL), and mesitylene (5 µL) as internal standard. The tube was sealed, brought out of the glove box, and the mixture was then stirred at the specified temperature for the required time. Yields were determined by ^1H NMR integration versus mesitylene.

*Table S3. Optimization of the reaction conditions for the deoxygenation of acetophenone **1k***

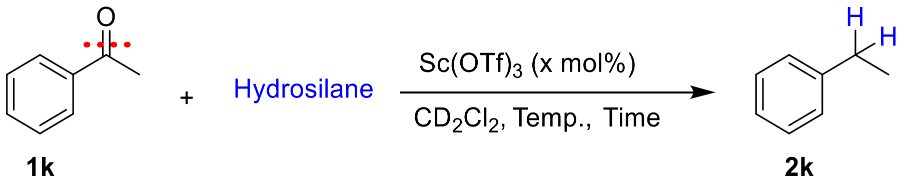

Entry	$Sc(OTf)_3$ mol%	Hydrosilane (equiv.)	Temp. (°C)	Time	Conv. %	NMR yield of 2k %
1	10	Et_3SiH (2)	120	22 h	40	34
2	10	Et_2SiH_2 (2)	120	22 h	>98	81
3	10	TMDS (2)	120	1 h	>98	>98
4	10	TMDS (1.1)	120	1 h	>98	74
5	5	TMDS (2)	120	1 h	>98	>98
6	5	TMDS (2)	80	1 h	>98	>98
7	5	TMDS (2)	80	30 min	>98	>98
8	5	TMDS (2)	60	4 h	>98	>98

3.2 General procedure for the deoxygenation of ketones

In a 2.5 mL *J.* Young NMR tube in a glovebox, ketone 1 (0.1 mmol, 1.0 equiv.) and TMDS (0.2 mmol, 2.0 equiv.), and mesitylene (5 µL) were added to a solution of $Sc(OTf)_3$ (5 mol%) in deuterated dichloromethane (0.5 mL). The tube was sealed, brought out of the glove box and the solution was then heated at 80 °C for the required time. The reaction progress was monitored by ^1H NMR spectroscopy. Yields were determined by ^1H NMR integration versus mesitylene as an internal standard.

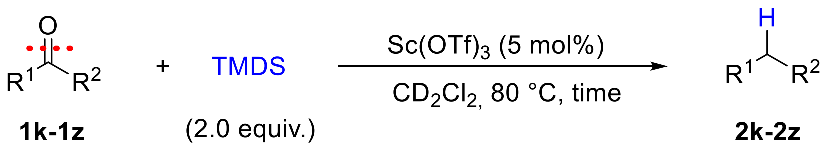

4 Hydrodehalogenation of alkyl halides

4.1 General procedure for the hydrodehalogenation of alkyl halides using TMDS

In a 2.5 mL *J.* Young NMR tube in a glovebox, alkyl halide **1** (0.1 mmol, 1.0 equiv.) and TMDS (0.1 mmol, 1.0 equiv.) and mesitylene (5 µL) were added to a solution of $Sc(OTf)_3$ (5 mol%) in deuterated dichloromethane (0.5 mL). The tube was sealed, brought out of the glove box and the solution was then heated at the required temperature for the specified time. The reaction progress was monitored by ^1H NMR spectroscopy. Yields were determined by ^1H NMR integration versus mesitylene as an internal standard.

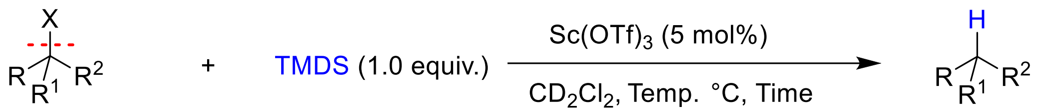

1aa-1ai **2aa-2ai**

R = H, Aryl, alkyl; R^1, R^2 = Aryl, alkyl
X = F, Cl, Br

4.2 General procedure for the hydrodehalogenation of alkyl halides using E3SiH

In a 2.5 mL *J.* Young NMR tube in a glovebox, alkyl halide **1** (0.1 mmol, 1.0 equiv.) and Et_3SiH (0.11 mmol, 1.1 equiv.) and mesitylene (5 µL) were added to a solution of $Sc(OTf)_3$ (5 mol%) in deuterated dichloromethane (0.5 mL). The tube was sealed, brought out of the glove box and the solution was then heated at the required temperature for the specified time. The reaction progress was monitored by ^1H NMR spectroscopy. Yields were determined by ^1H NMR integration versus mesitylene as an internal standard.

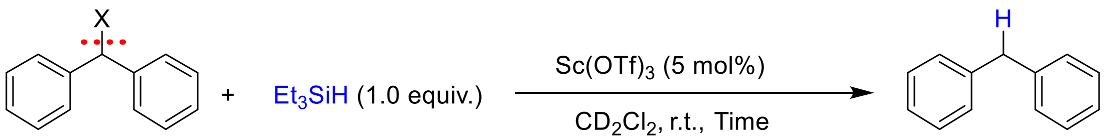

1ab-1ad **2ab-2ad**

X = F, Cl, Br

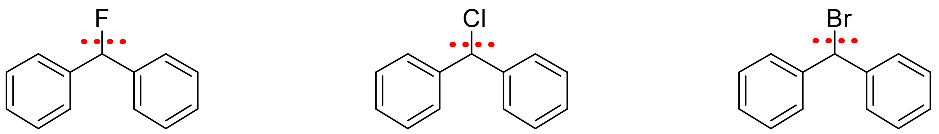

2ab >98%
5 min

2ac >98%
5 min

2ad >98%
20 min

5 Chemoselectivity

5.1 General procedure for the chemoselectivity study: alcohols *vs* ketones

In a 2.5 mL *J.* Young NMR tube in a glovebox, ketone (0.1 mmol, 1.0 equiv.), alcohol (0.1 mmol, 1.0 equiv.), Et_3SiH (0.1 mmol, 1.0 equiv.) and mesitylene (5 µL) were added to a solution of $Sc(OTf)_3$ (5 mol%) in deuterated dichloromethane (0.5 mL). The tube was sealed, brought out of the glove box and the solution was then heated at 80 °C for the required time. The reaction progress was monitored by ^1H NMR spectroscopy. Yields were determined by ^1H NMR integration versus mesitylene as an internal standard.

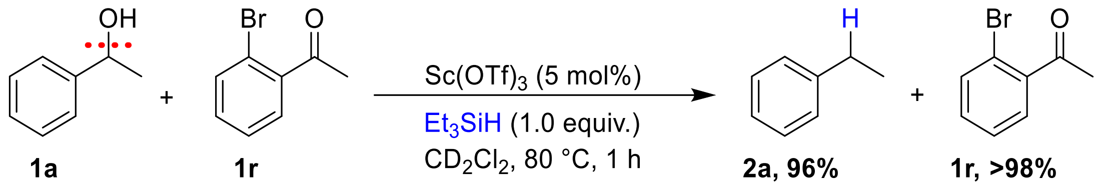

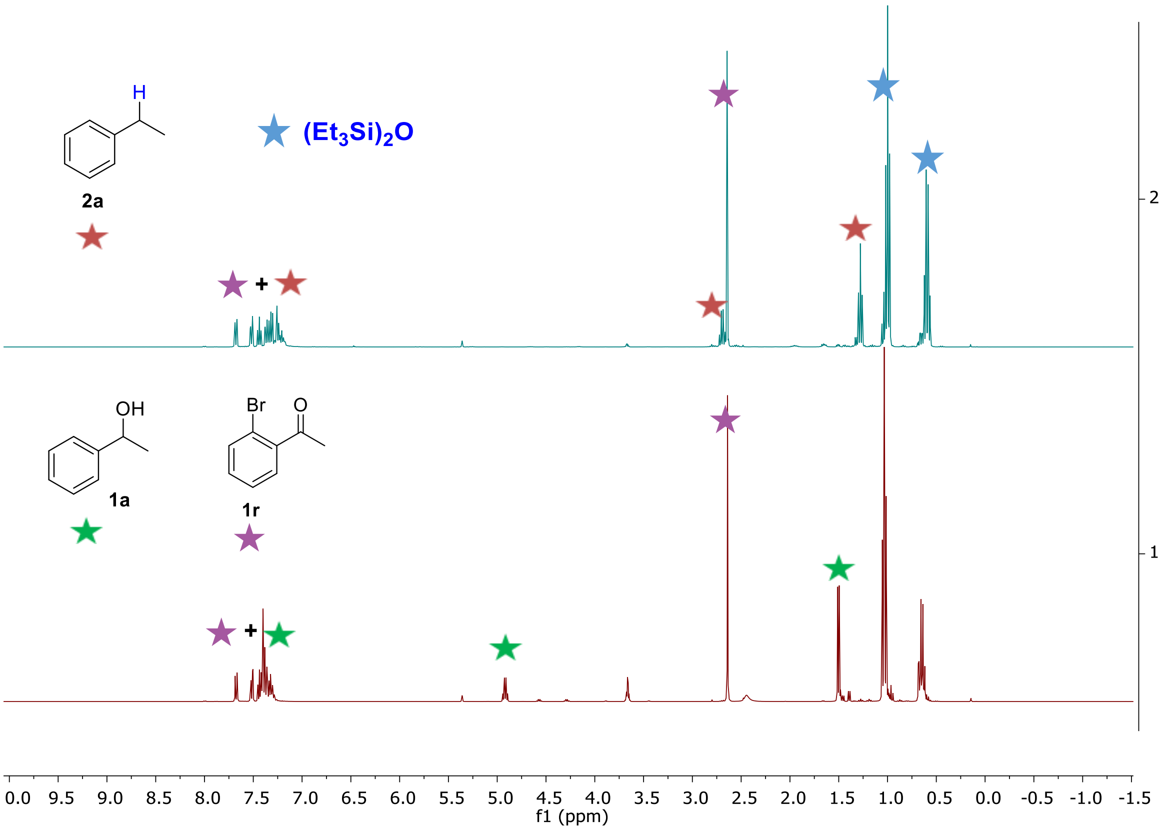

*Figure S1. ^1H NMR of the crude reaction mixture of the chemoselective deoxygenation of.alcohol **1a** vs ketone **1r**. (**Bottom**) : The crude reaction mixture at r.t., time t=0. (**Top**) : The reaction progress after heating at 80 °C for 1 h.*

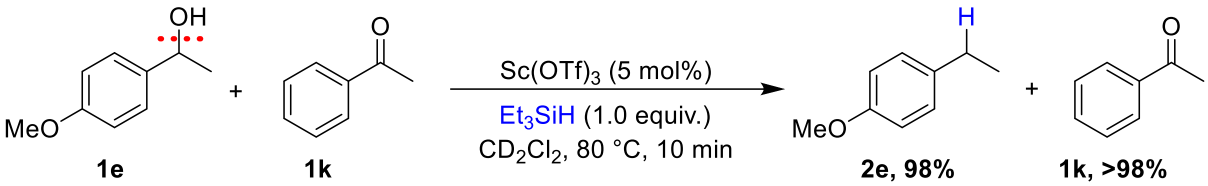

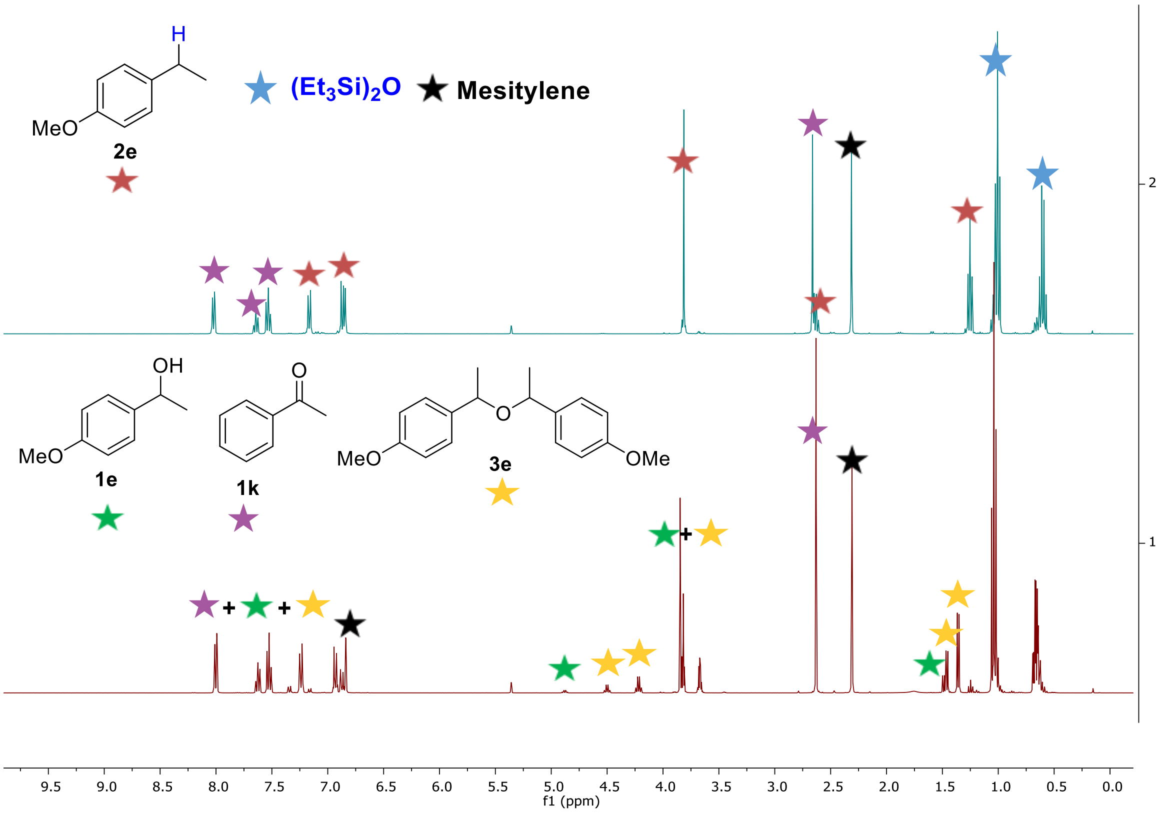

Figure S2. ^{1}*H NMR of the crude reaction mixture of the chemoselective deoxygenation of.alcohol* ***1e*** *vs ketone* ***1k****. (**Bottom**) : The crude reaction mixture at r.t., time t=0, (89% of* ***1e*** *was converted to the ether intermediate* ***3e****). (**Top**) : The reaction progress after heating at 80 °C for 10 min.*

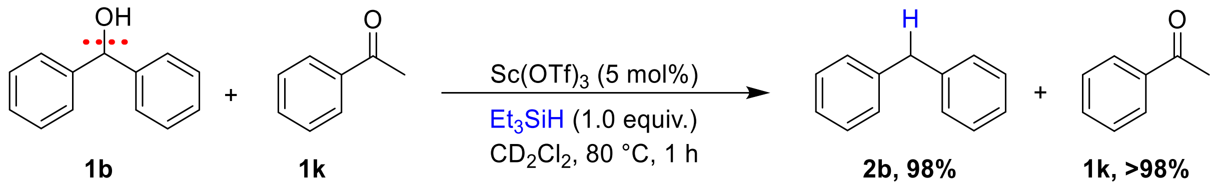

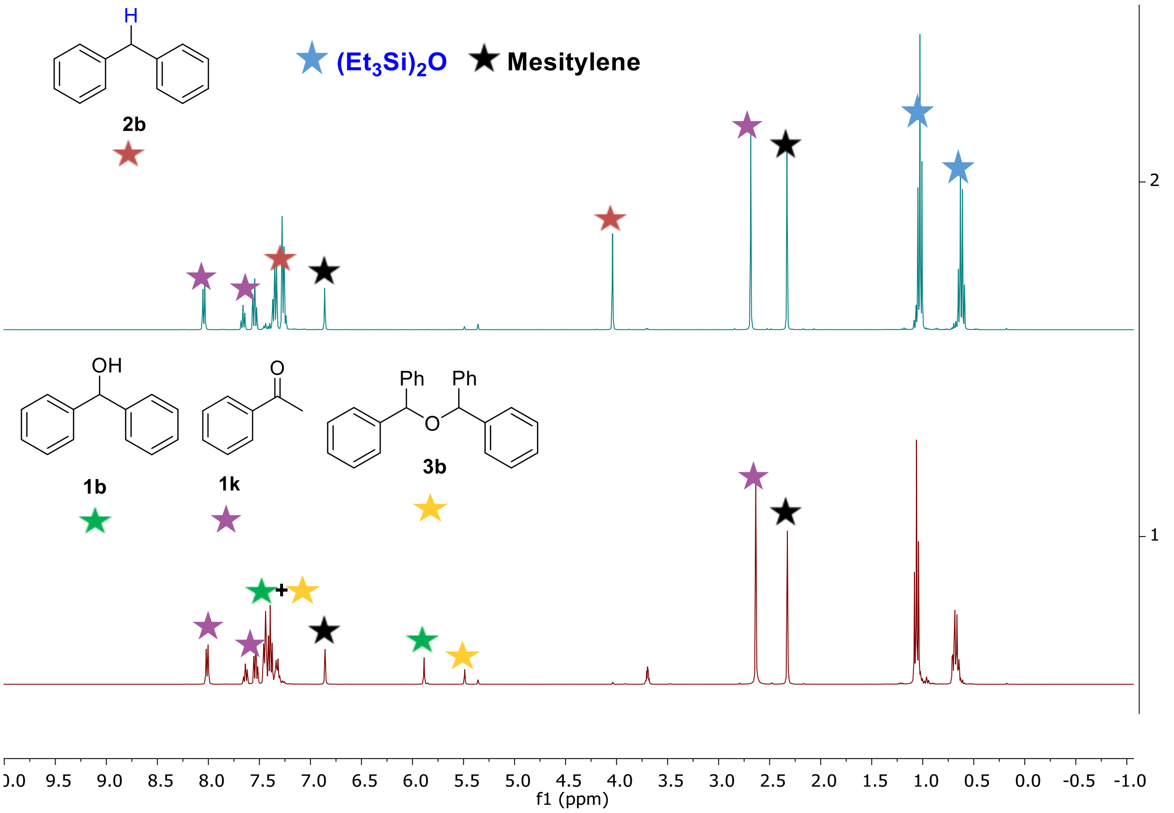

Figure S3. [1]*H NMR of the crude reaction mixture of the chemoselective deoxygenation of.alcohol* ***1b*** *vs ketone* ***1k****.* ***(Bottom)*** *: The crude reaction mixture at r.t. time t=0, (35% of* ***1b*** *was converted to the ether intermediate* ***3b****).* ***(Top)*** *: The reaction progress after heating at 80 °C for 1 h.*

5.2 General procedure for the chemoselectivity study: BCF/Et_3SiH system

In a 2.5 mL *J.* Young NMR tube in a glovebox, 1-phenylethanol (0.1 mmol, 1.0 equiv.), 1-(2-bromophenyl)ethan-1-one (0.1 mmol, 1.0 equiv.), Et_3SiH (*x* equiv.), and mesitylene (5 µL) were added to a solution of $B(C_6F_5)_3$ (10 mol%) in deuterated dichloromethane (0.5 mL). The tube was sealed, brought out of the glove box and the solution was stirred at room temperature for 22 h. The reaction progress was monitored by ^{1}H NMR spectroscopy. Yields were determined by ^{1}H NMR integration versus mesitylene as an internal standard.

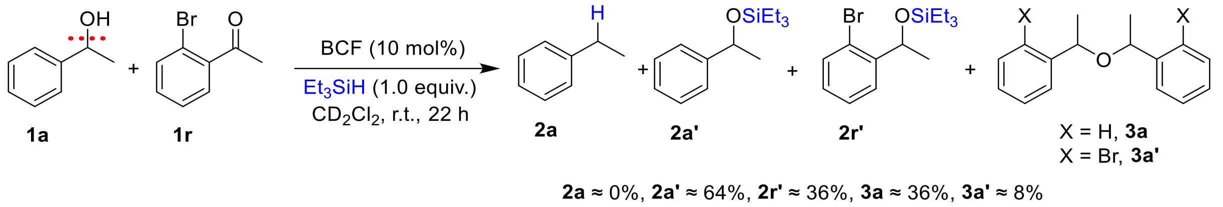

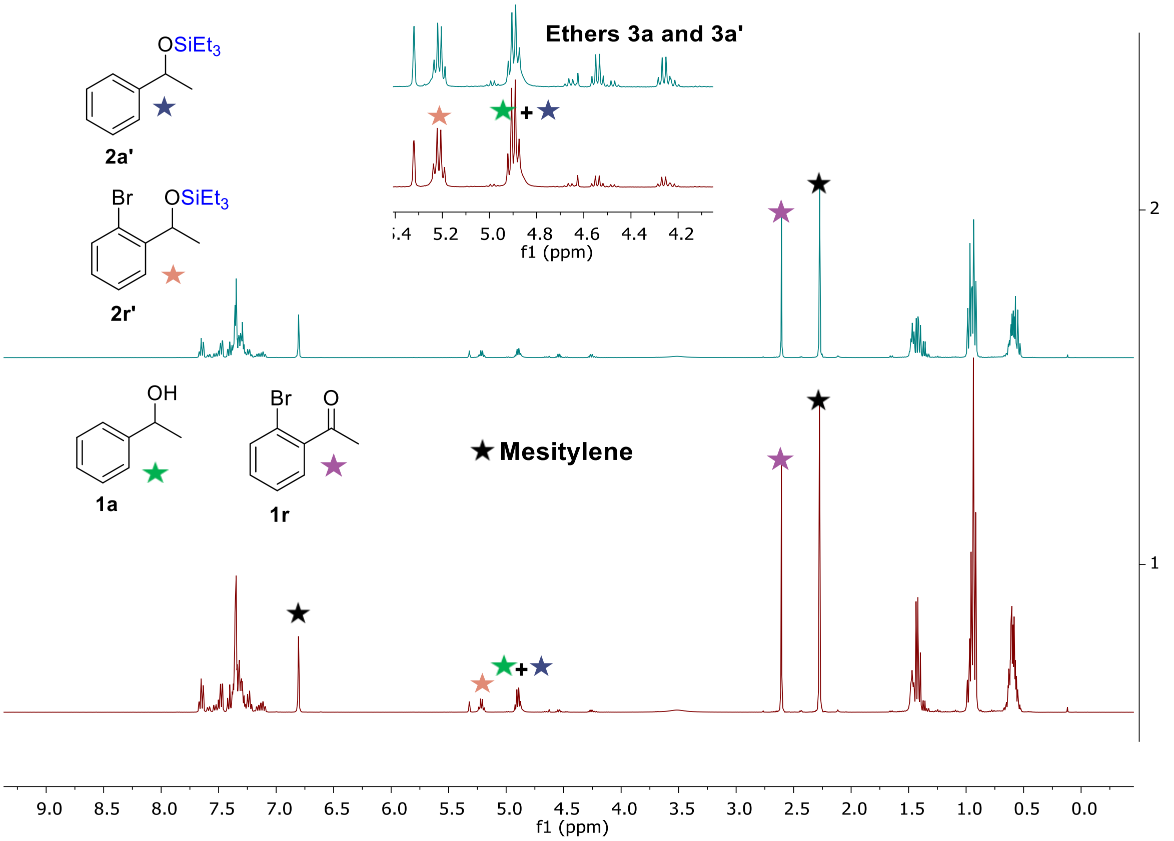

Figure S4. [1]*H NMR of the crude reaction mixture of the chemoselective deoxygenation of.alcohol* ***1a*** *vs ketone* ***1r*** *using BCF (10 mol%)/Et₃SiH (1.0 equiv.) system.* ***(Bottom)*** *: The crude reaction mixture at r.t., time t=0.* ***(Top)*** *: The reaction progress after 22 h at r.t.*

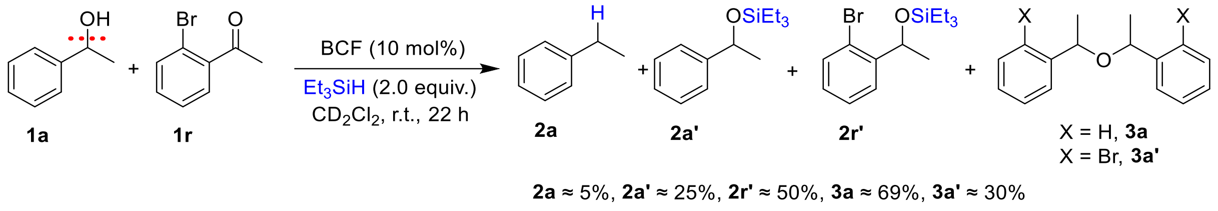

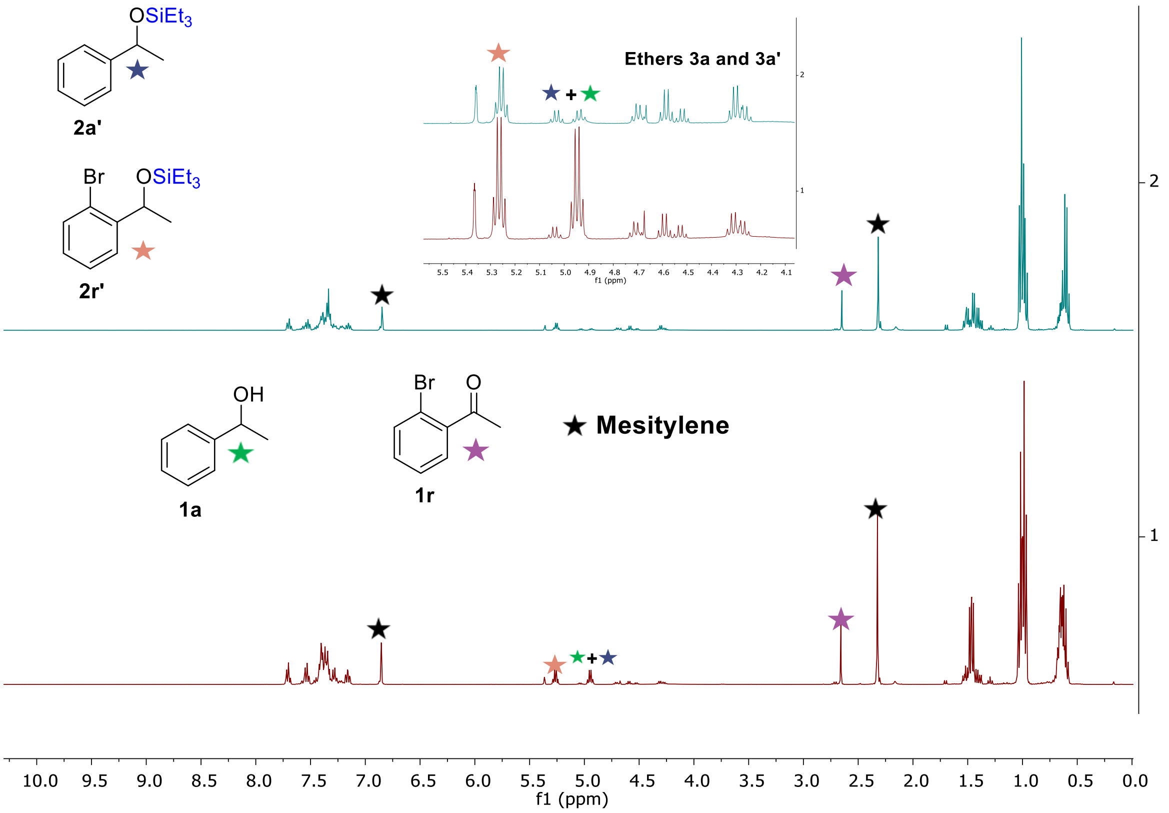

*Figure S5. ^{1}H NMR of the crude reaction mixture of the chemoselective deoxygenation of.alcohol **1a** vs ketone **1r** using BCF (10 mol%)/Et₃SiH (2.0 equiv.) system. (**Bottom**) : The crude reaction mixture at r.t., time t=0. (**Top**) : The reaction progress after 22 h at r.t.*

5.3 General procedure for the chemoselectivity study: $InCl_3$/Ph_2ClSiH system

In a 2.5 mL *J.* Young NMR tube in a glovebox, 1-phenylethanol (0.1 mmol, 1.0 equiv.), 1-(2-bromophenyl)ethan-1-one (0.1 mmol, 1.0 equiv.), Ph_2ClSiH (*x* equiv.) and mesitylene (5 µL) were added on to a solution of $InCl_3$ (10 mol%) in deuterated dichloromethane (0.5 mL). The tube was sealed, brought out of the glove box and the solution was stirred at room temperature for 22 h. The reaction progress was monitored by ^{1}H NMR spectroscopy. Yields were determined by ^{1}H NMR integration versus mesitylene as an internal standard.

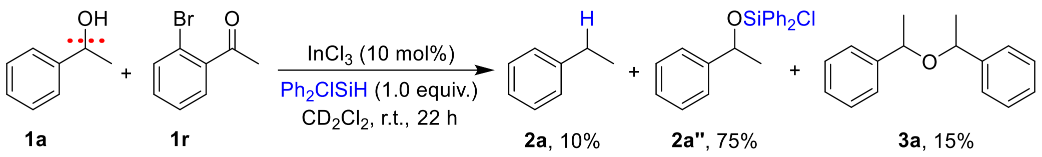

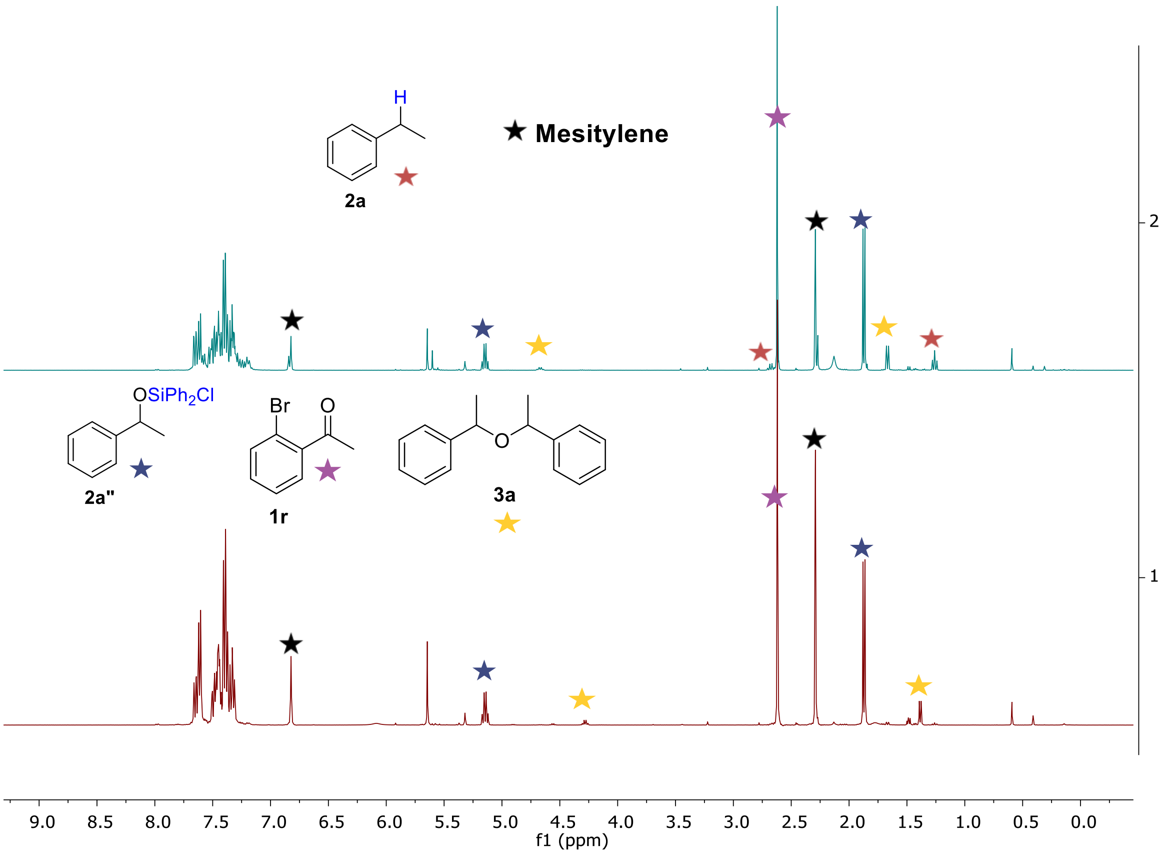

*Figure S6. ^{1}H NMR of the crude reaction mixture of the chemoselective deoxygenation of.alcohol **1a** vs ketone **1r** using $InCl_3$ (10 mol%)/Ph_2SiClH (1.0 equiv.) system. (**Bottom**) : The crude reaction mixture at r.t., time t=0. (**Top**) : The reaction progress after 22 h at r.t.*

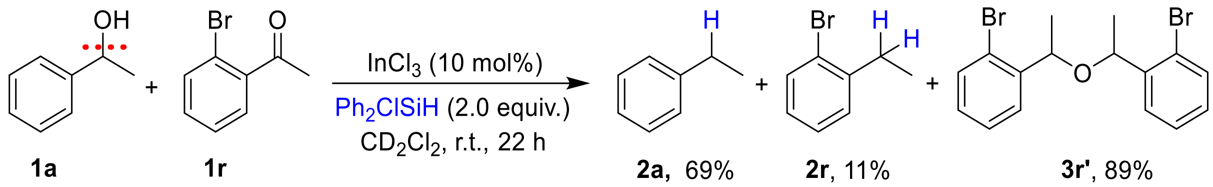

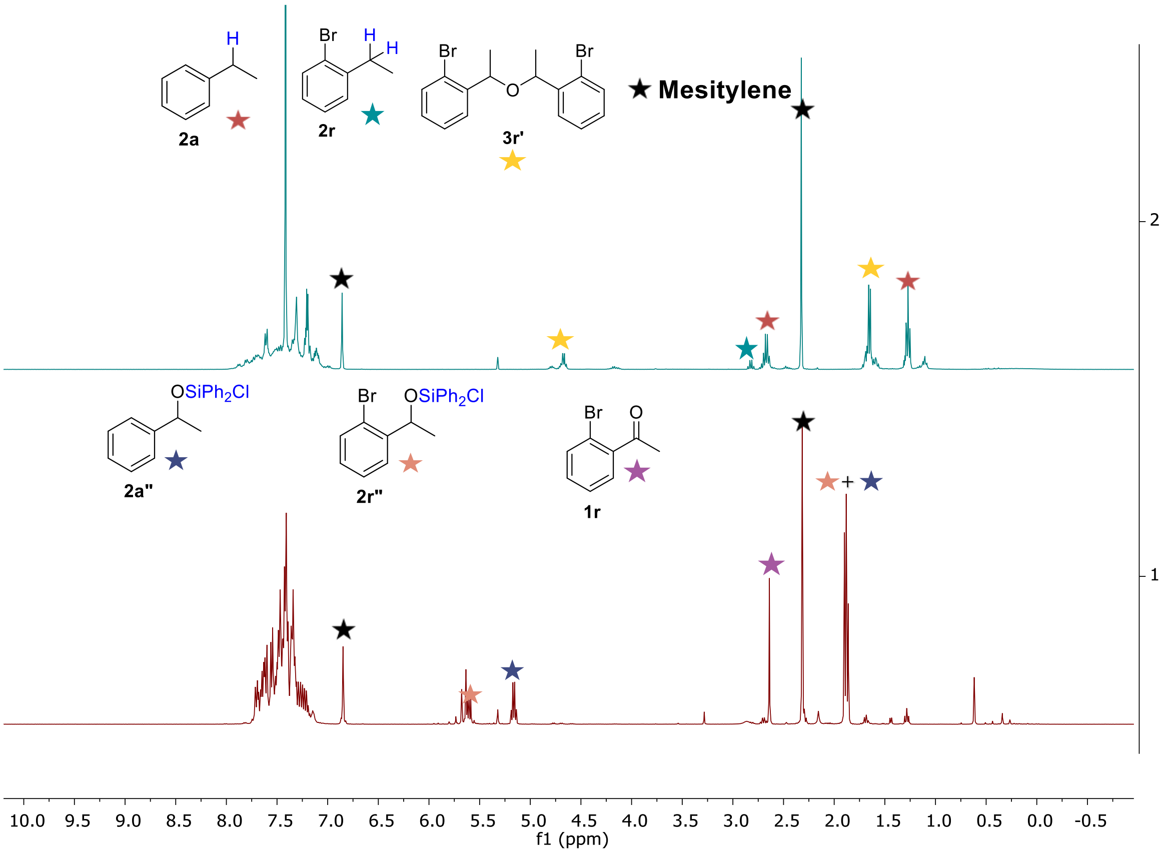

*Figure S7. ^{1}H NMR of the crude reaction mixture of the chemoselective deoxygenation of.alcohol **1a** vs ketone **1r** using $InCl_3$ (10 mol%)/Ph_2SiClH (2.0 equiv.) system. (**Bottom**) : The crude reaction mixture at r.t., time t=0. (**Top**) : The reaction progress after 22 h at r.t.*

5.4 Regioselective deoxygenation of internal 1,2-diol (1aj) at the secondary position

In a 2.5 mL *J*. Young NMR tube in a glovebox, diol **1aj** (0.1 mmol, 1.0 equiv.) and Et_3SiH (0.1 mmol, 1.0 equiv.) and mesitylene (5 µL) were added on to a solution of $Sc(OTf)_3$ (5 mol%) in deuterated dichloromethane (0.5 mL). The tube was sealed, brought out of the glove box and the solution was then heated at 100 °C for 18 h. The reaction progress was monitored by ^{1}H NMR spectroscopy. Yields were determined by ^{1}H NMR integration versus mesitylene as an internal standard.

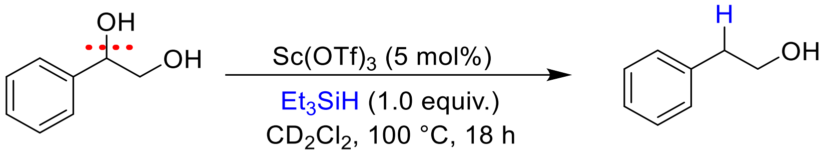

1aj **2aj, >98%**

5.5 Chemoselectivite deoxygenation of ketoprofen 1ak

In a 2.5 mL *J*. Young NMR tube in a glovebox, ketoprofen **1ak** (0.1 mmol, 1.0 equiv.) and TMDS (0.2 mmol, 2.0 equiv.) and mesitylene (5 µL) were added on to a solution of $Sc(OTf)_3$ (5 mol%) in deuterated dichloromethane (0.5 mL). The tube was sealed, brought out of the glove box and the solution was then heated at 80 °C for 30 minutes. The reaction progress was monitored by ^{1}H NMR spectroscopy. Yields were determined by ^{1}H NMR integration versus mesitylene as an internal standard.

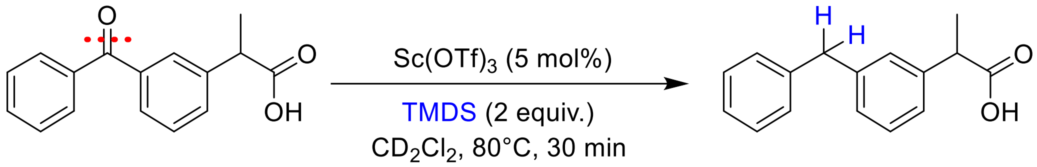

1ak **2ak, >98%**

6 Mechanistic Study

6.1 Control experiments for alcohol deoxygenation

6.1.1 Reaction in the absence of hydrosilane

In a 2.5 mL J. Young NMR tube in a glove box, 1-phenylethanol **1a** (12 mg, 0.1 mmol, 1.0 equiv.) and mesitylene (5 µL) were added on to a solution of $Sc(OTf)_3$ (2.5 mg, 5 mol%) in deuterated dichloromethane (0.5 mL). The tube was sealed, brought out of the glove box and the solution was then heated at 80 °C for 30 minutes, **1a** was completely converted to **3a**. To the crude reaction mixture, (17.6 µL, 0.11 mmol, 1.1 equiv.) of Et_3SiH was added and heated at 80 °C for 1 h, leading to the full conversion of **3a** to **2a**. The reaction progress was monitored by ^1H NMR spectroscopy. Yields were determined by ^1H NMR integration versus mesitylene as an internal standard.

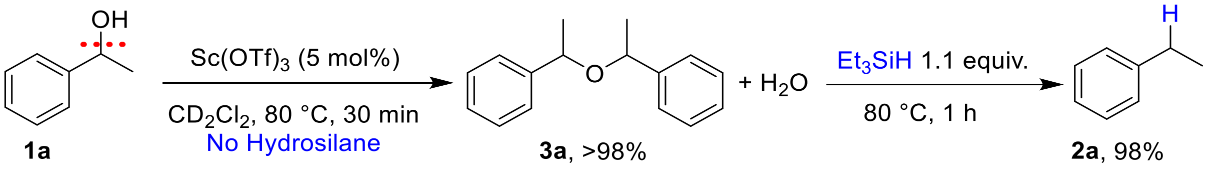

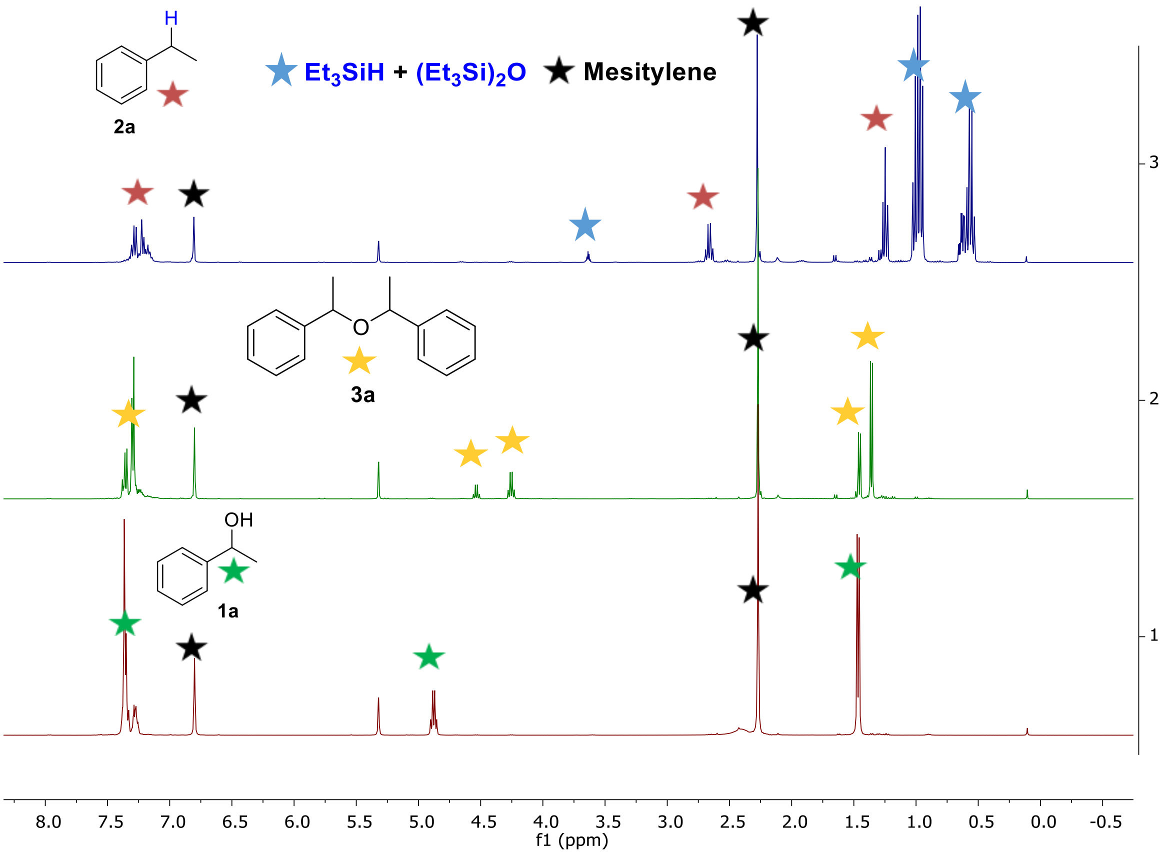

*Figure S8. ^1H NMR of the crude reaction mixture. (**Bottom**) : Reacting alcohol **1a** with (5 mol%) $Sc(OTf)_3$, and (5 µL) mesitylene in CD_2Cl_2 without hydrosilane at r.t., time t=0. (**Middle**) : The reaction progress after heating at 80 °C for 30 min (**1a** converted to **3a**). (**Top**) : The spectra obtained after adding (1.1 equiv.) of Et_3SiH and heated at 80 °C for 1 h (**3a** converted to **2a**).*

6.1.2 Preparation of (oxybis(ethane-1,1-diyl))dibenzene 3a

A 20 mL Schlenk tube in a glovebox was charged with 1-phenylethanol **1a** (1.0 mmol, 122 mg), $Sc(OTf)_3$ (5 mol%, 25 mg), and dichloromethane (5.0 mL). The Schlenk tube was sealed, brought out of the glove box, and the mixture was then stirred at 80 °C for 1 h. The mixture was then filtered through Celite and dried with $MgSO_4$. The solvent was removed under vacuum to afford **3a** quantitatively as a mixture of 2 isomers.

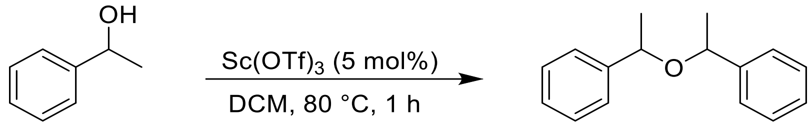

1a **3a**

6.1.3 Reaction starting from the ether 3a

In a 2.5 mL *J.* Young NMR tube in a glovebox, ether **3a** (22.6 mg, 0.1 mmol, 1.0 equiv.), Et_3SiH (32 µL, 0.2 mmol, 2.0 equiv), and mesitylene (5 µL) were added on to a solution of $Sc(OTf)_3$ (2.5 mg, 5 mol%) in deuterated dichloromethane (0.5 mL). The tube was sealed, brought out of the glove box and the solution was then heated at 80 °C for 10 min, resulting in the full conversion of **3a** to **2a**. The reaction progress was monitored by ^{1}H NMR spectroscopy. Yields were determined by ^{1}H NMR integration versus mesitylene as an internal standard.

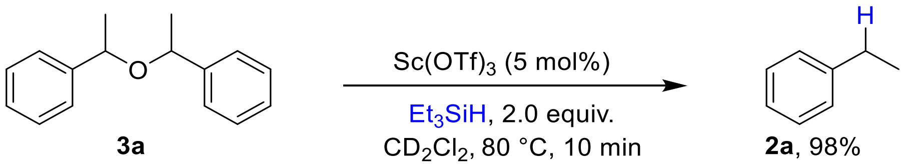

3a **2a**, 98%

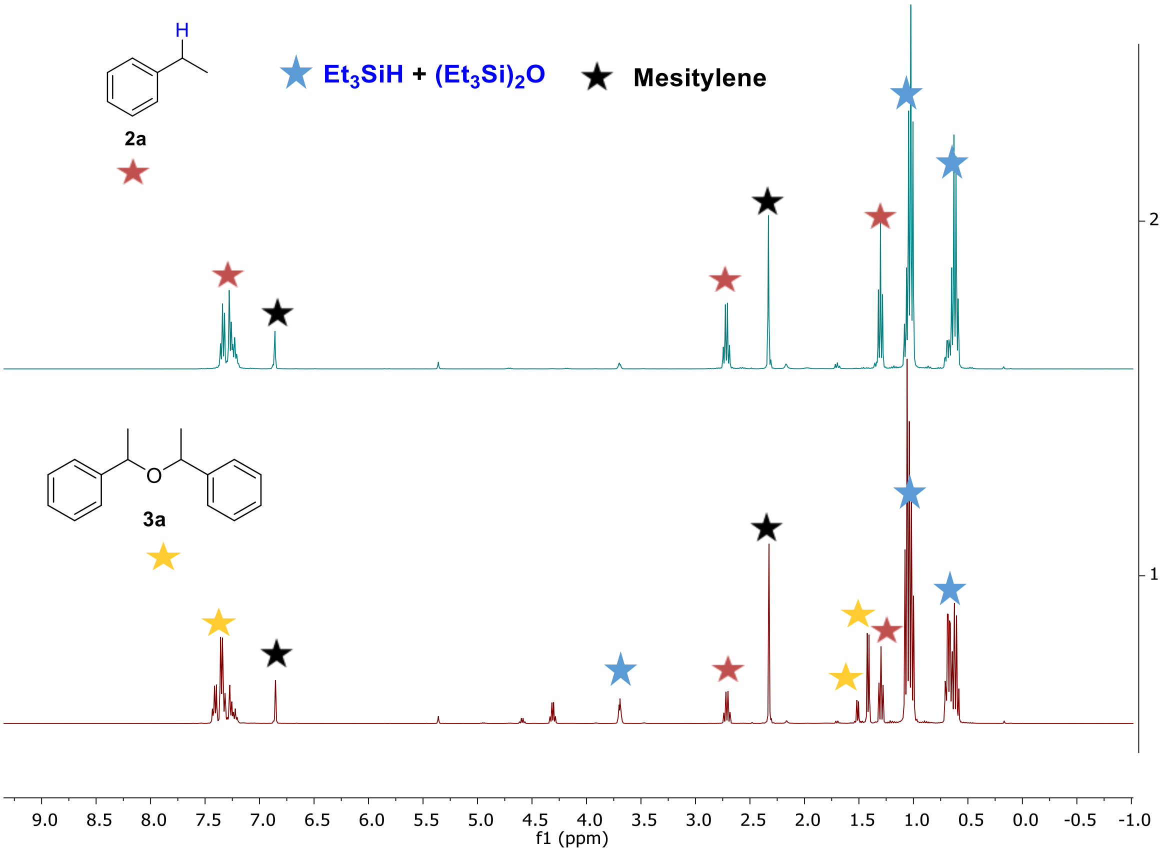

*Figure S9. ^{1}H NMR of the crude reaction mixture. (**Bottom**) : Reacting ether **3a** with (5 mol%) Sc(OTf)$_3$, (2.0 equiv.) Et_3SiH, and (5 µL) mesitylene in CD_2Cl_2 at r.t., time t=0. (**Top**) : The reaction progress after heating at 80 °C for 10 min.*

6.1.4 Performing the reaction in the presence of H_2O

In a 2.5 mL J. Young NMR tube in a glove box, 1-phenylethanol **1a** (12 mg, 0.1 mmol, 1.0 equiv.), Et_3SiH (17.6 µL, 0.11 mmol, 1.1 equiv), H_2O (1.8µL, 1.0 equiv.), and mesitylene (5 µL) were added on to a solution of Sc(OTf)$_3$ (2.5 mg, 5 mol%) in deuterated dichloromethane (0.5 mL). The tube was sealed, brought out of the glove box and the solution was then heated at 80 °C for 22 h, **1a** was converted to **2a** in 85% yield. The reaction progress was monitored by ^1H NMR spectroscopy. Yields were determined by ^1H NMR integration versus mesitylene as an internal standard.

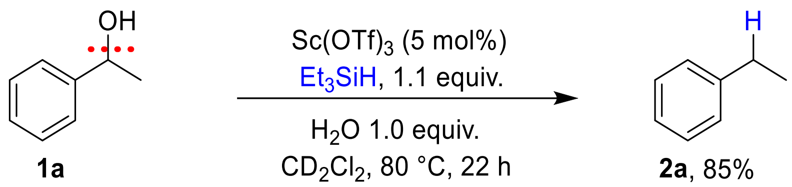

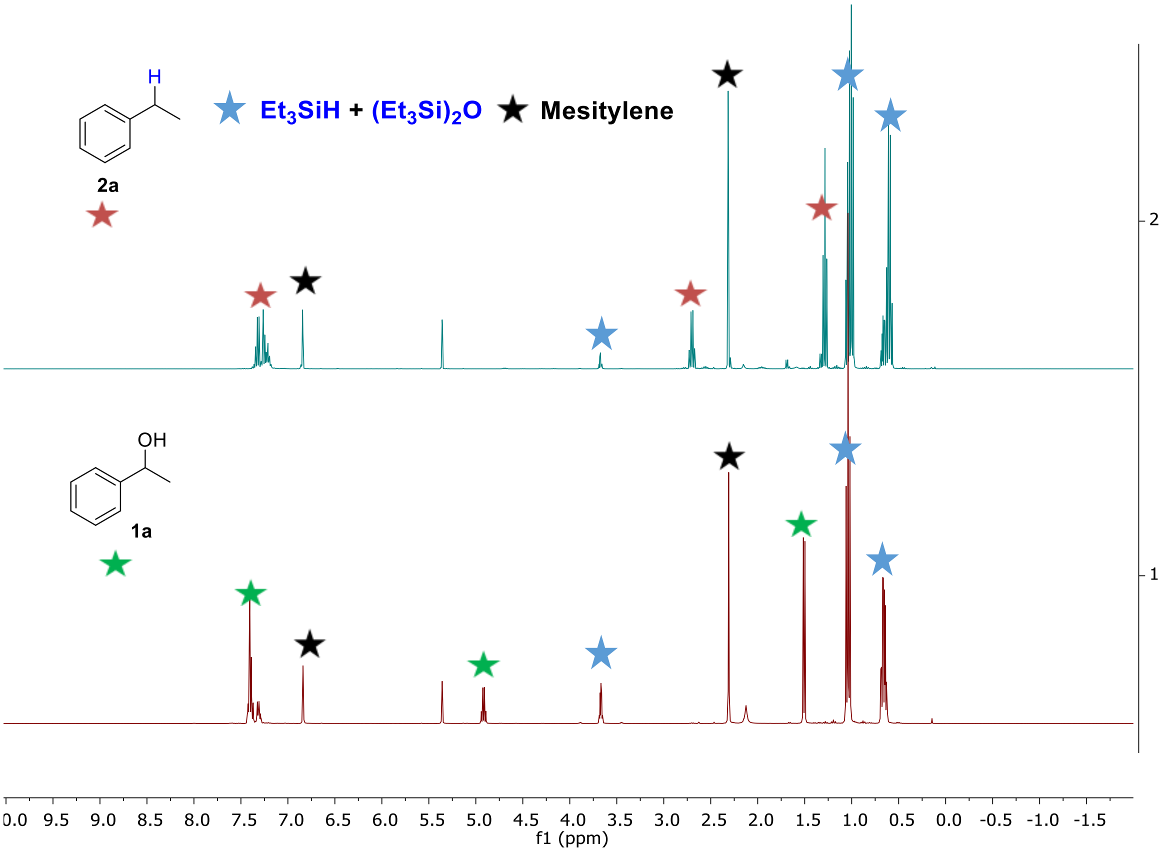

*Figure S10. ^{1}H NMR of the crude reaction mixture. (**Bottom**) : Reacting alcohol **1a** with (5 mol%) Sc(OTf)$_3$, (1.1 equiv.) Et$_3$SiH, (1.0 equiv.) of H$_2$O, and (5 µL) mesitylene in CD$_2$Cl$_2$ at r.t., time t=0. (**Top**) : The reaction progress after heating at 80 °C for 22 h.*

6.1.5 Reaction starting from the silyl ether 2a′

In a 2.5 mL *J*. Young NMR tube in a glovebox, silyl ether **2a′** (23.6 mg, 0.1 mmol, 1.0 equiv.), Et$_3$SiH (17.6 µL, 0.11 mmol, 1.1 equiv), and mesitylene (5 µL) were added on to a solution of Sc(OTf)$_3$ (2.5 mg, 5 mol%) in deuterated dichloromethane (0.5 mL). The tube was sealed, brought out of the glove box. **2a′** was converted instantly at r.t. into 80% of **2a** and 18% of **3a**. The solution was then stirred at room temperature for 10 min to give **2a** quantitatively. The reaction progress was monitored by ^{1}H NMR spectroscopy. Yields were determined by ^{1}H NMR integration versus mesitylene as an internal standard.

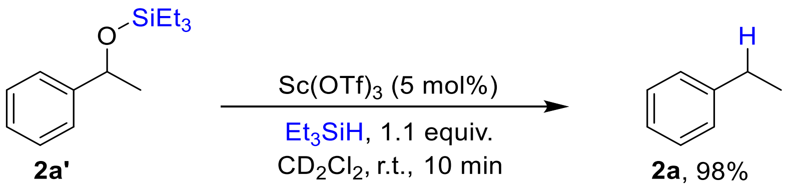

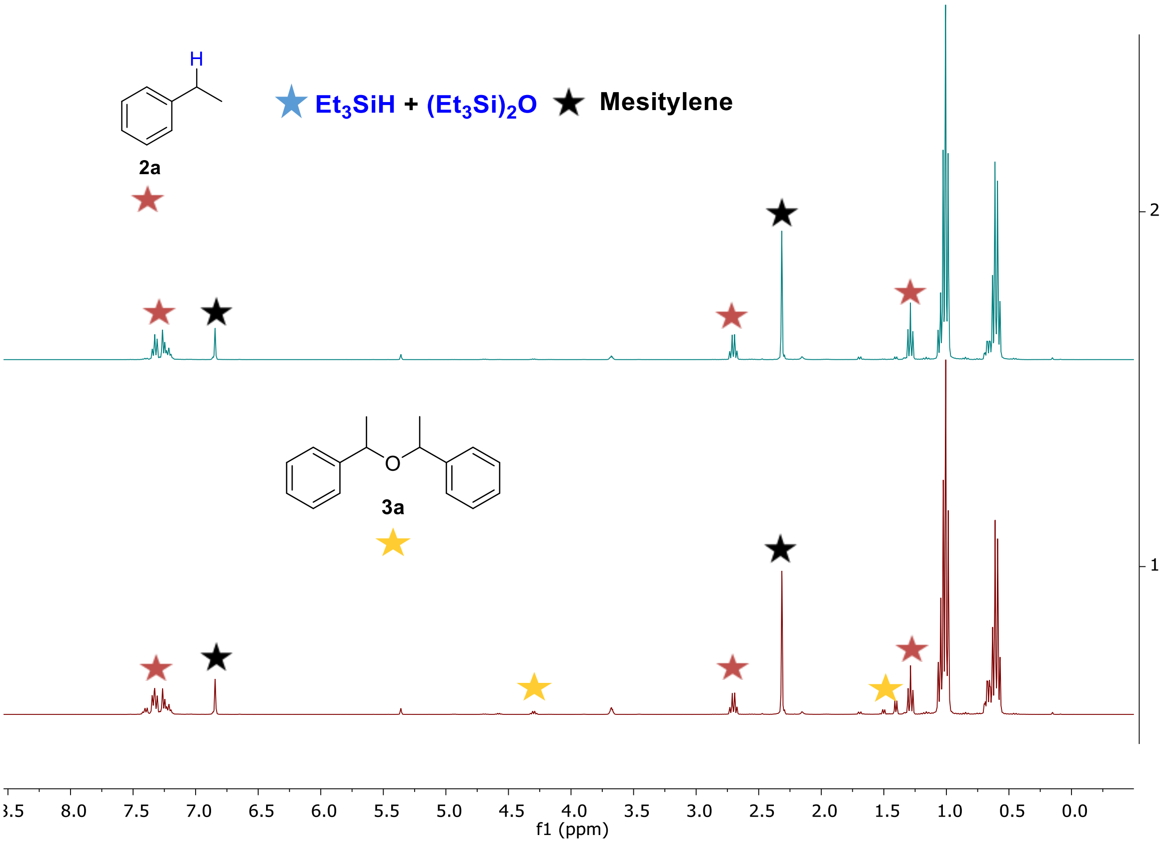

Figure S11. ^{1}*H NMR of the crude reaction mixture. (**Bottom**) : Reacting silylether **2a'** with (5 mol%) Sc(OTf)*$_3$*, (1.1 equiv.) Et*$_3$*SiH, and (5 µL) mesitylene in CD*$_2$*Cl*$_2$ *at r.t.at time t=0. (**Top**) : The reaction progress after 10 min at r.t..*

6.2 Formation of halosilane: hydrodehalogenation of alkyl halides 1ab–1ad using E$_3$SiH

In a 2.5 mL *J.* Young NMR tube in a glovebox, alkyl halide **1ab–1ad** (0.1 mmol, 1.0 equiv.) and Et$_3$SiH (0.11 mmol, 1.1 equiv.) and mesitylene (5 µL) were added to a solution of Sc(OTf)$_3$ (5 mol%) in deuterated dichloromethane (0.5 mL). The tube was sealed, brought out of the glove box and the solution was then heated at the required temperature for the specified time. The reaction progress was monitored by ^{1}H NMR spectroscopy. Yields were determined by ^{1}H NMR integration versus mesitylene as an internal standard.

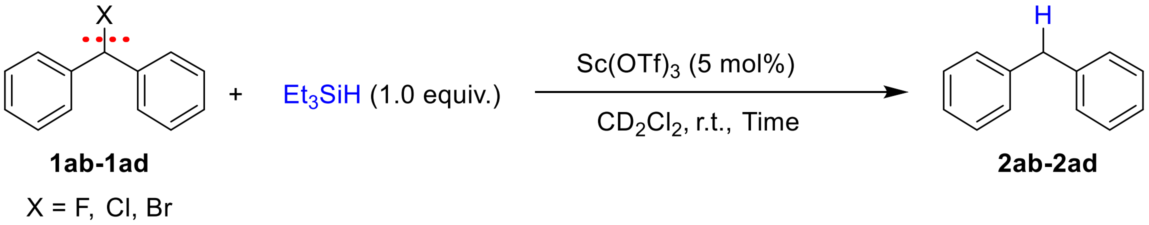

Hydrodehalogenation of substrates **1ab–1ad** using Et$_3$SiH enabled the identification of the dehalogenation byproducts. Analysis of the crude reaction mixtures by NMR revealed the presence of Et$_3$SiF, Et$_3$SiCl, and Et$_3$SiBr.

Et$_3$SiF[5]: **^{1}H NMR** (400 MHz, CD$_2$Cl$_2$) δ 1.02 (t, J = 8.0 Hz, 9H), 0.76 – 0.67 (m, 6H).

^{13}C NMR (101 MHz, CD$_2$Cl$_2$) δ 6.28 ($^3J_{C\text{-}F}$ = 2.1 Hz), 5.22 ($^2J_{C\text{-}F}$ = 14.0 Hz).

^{19}F NMR (376 MHz, CD$_2$Cl$_2$) δ -175.89.

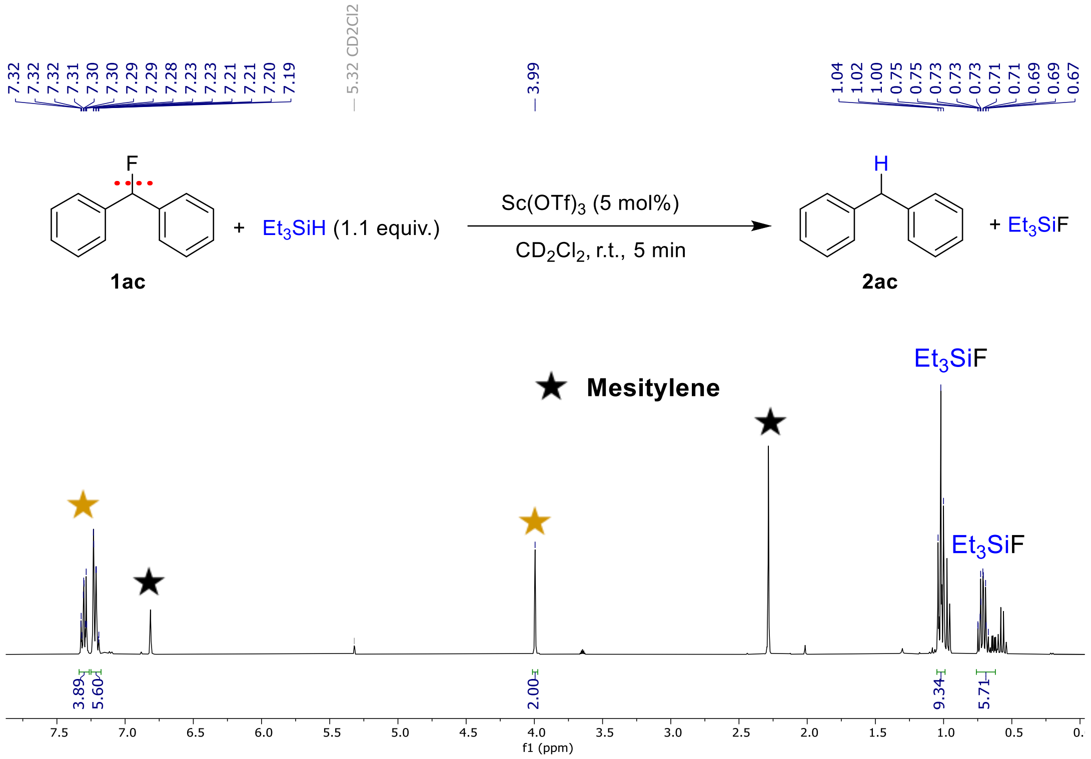

*Figure S12. ¹H NMR spectrum of the crude reaction mixture of **Et₃SiF**.*

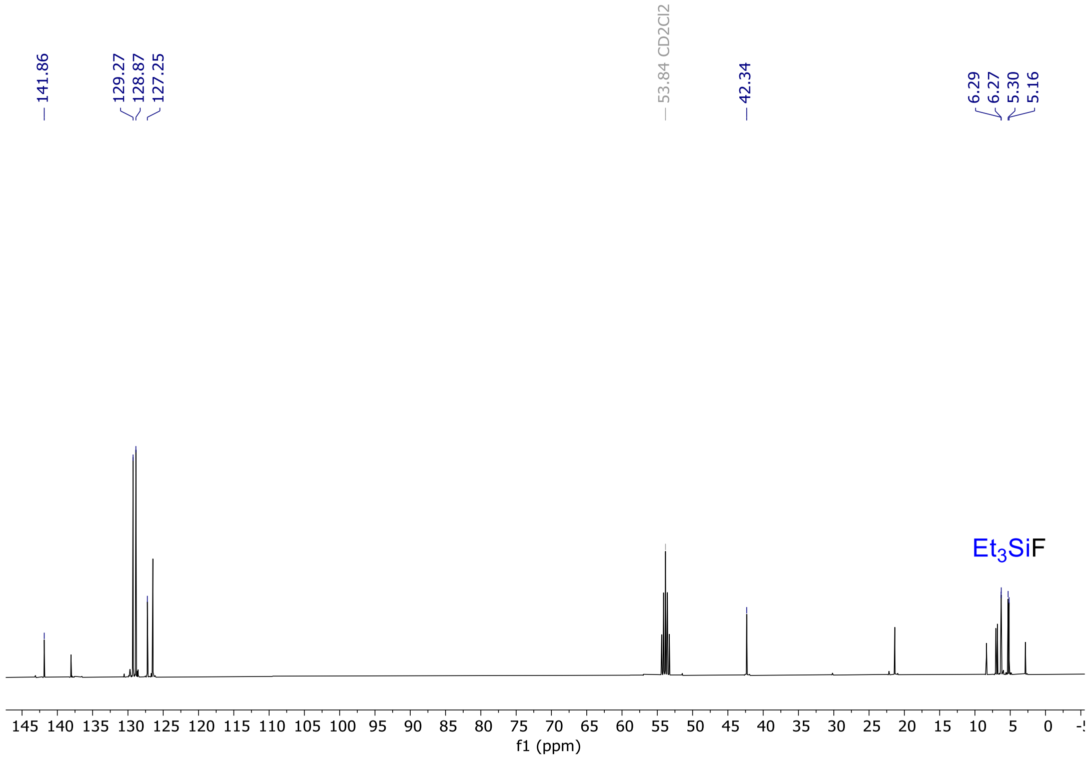

*Figure S13. ¹³C{¹H} NMR spectrum of the crude reaction mixture of **Et₃SiF**.*

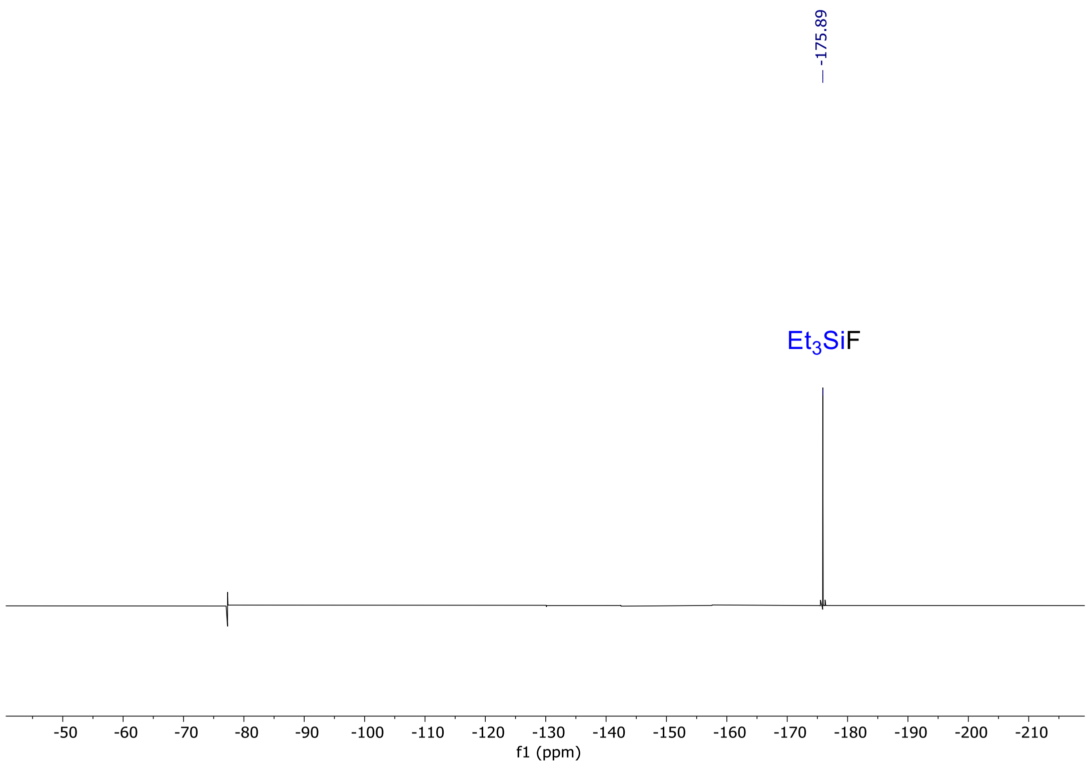

Figure S14. *$^{19}F\{^1H\}$ NMR spectrum of the crude reaction mixture of* ***Et_3SiF****.*

Et_3SiCl[6]: **^{1}H NMR** (400 MHz, CD_2Cl_2) δ 1.05 (t, J = 7.7 Hz, 9H), 0.85 (q, J = 7.9 Hz, 6H). **^{13}C NMR** (101 MHz, CD_2Cl_2) δ 7.66, 6.82.

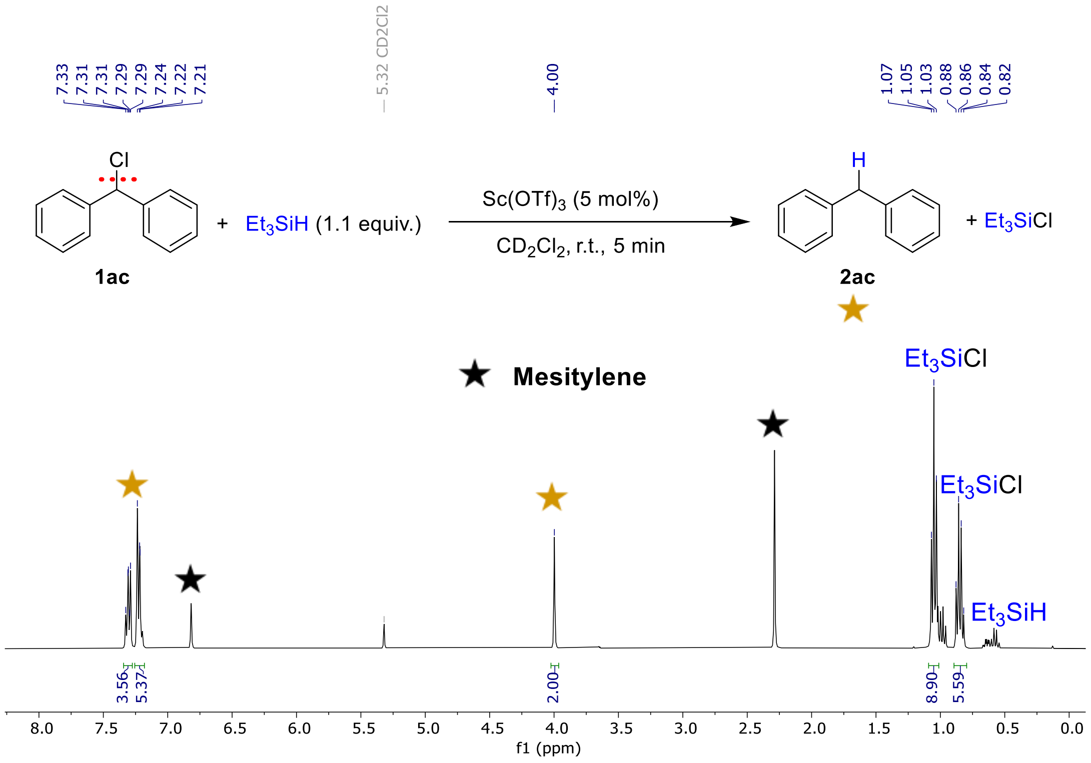

Figure S15. *^1H NMR spectrum of the crude reaction mixture of* ***Et_3SiCl****.*

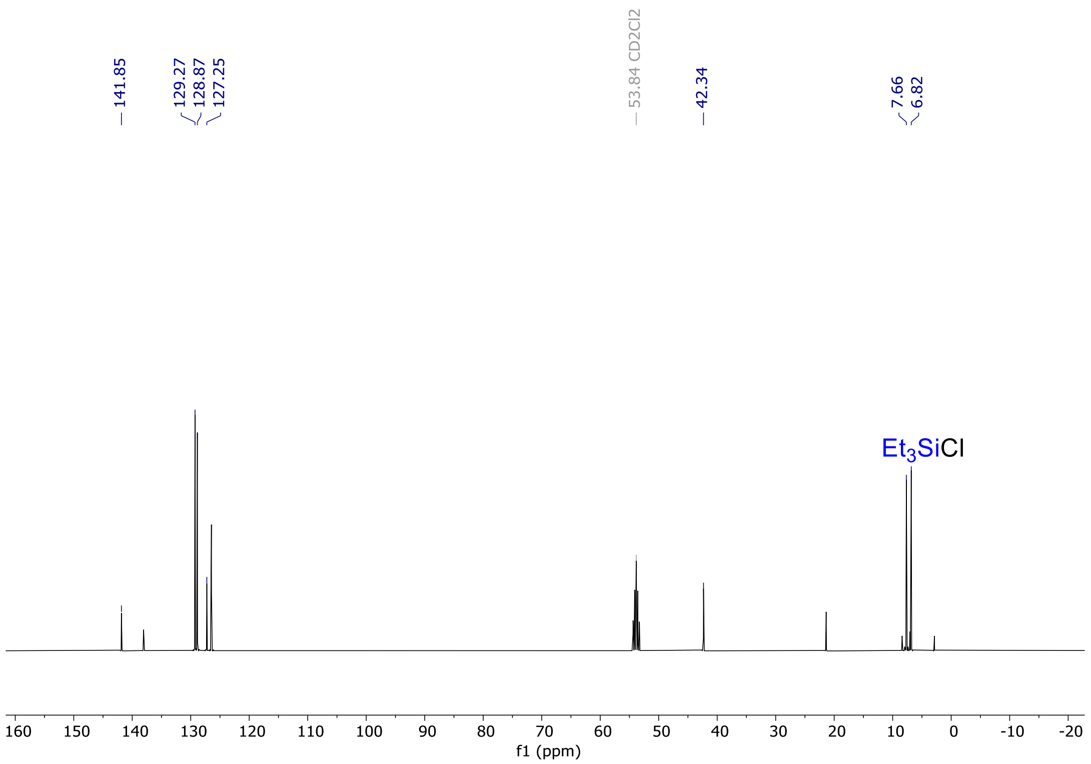

Figure S16. $^{13}C\{^{1}H\}$ NMR spectrum of the crude reaction mixture of ***Et_3SiCl****.*

Et_3SiBr[6]: **^{1}H NMR** (400 MHz, CD_2Cl_2) δ 11.08 – 1.02 (m, 9H), 0.94 (q, J = 7.2, 6.8 Hz, 6H).

^{13}C NMR (101 MHz, CD_2Cl_2) δ 8.21, 7.29.

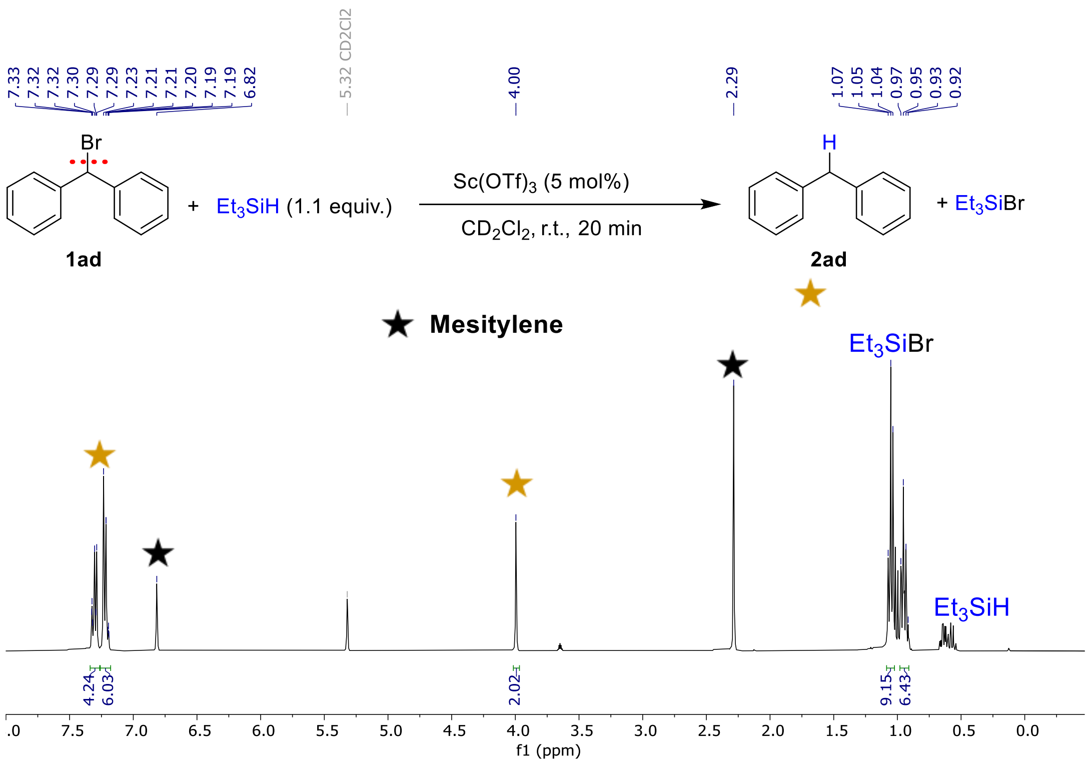

Figure S17. ^{1}H NMR spectrum of the crude reaction mixture of ***Et_3SiBr****.*

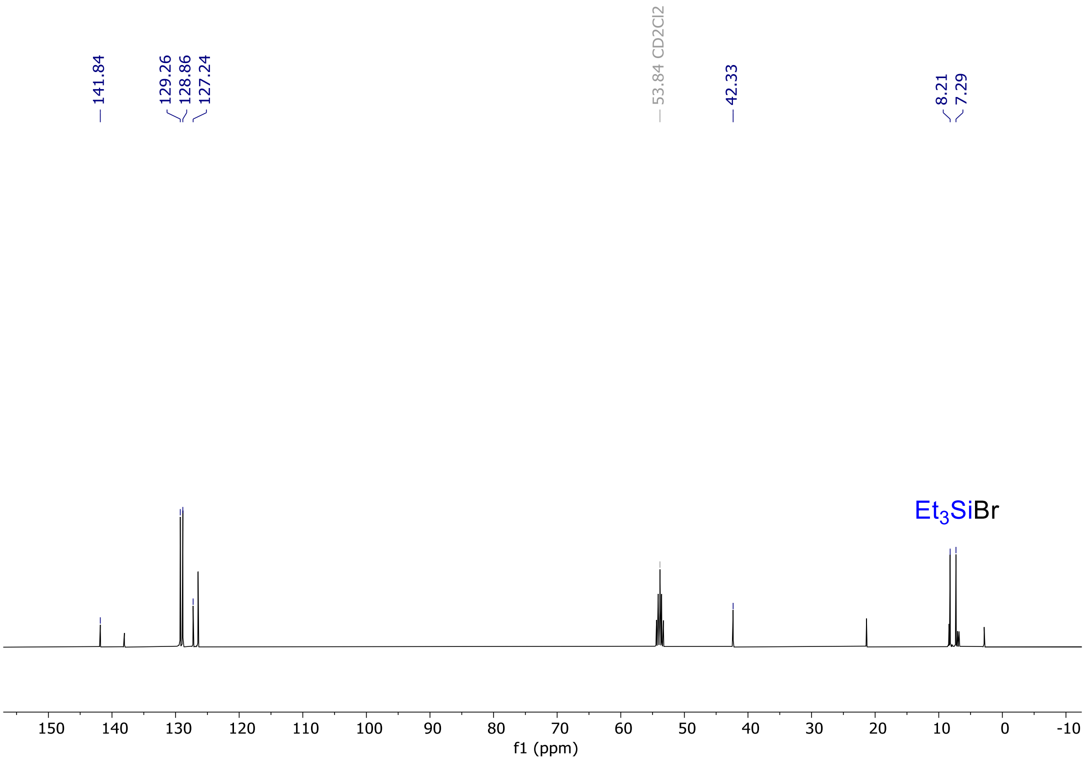

Figure S18. *$^{13}C\{^{1}H\}$ NMR spectrum of the crude reaction mixture of* ***Et_3SiBr***.

7 Characterization of products

All the data presented below were obtained from the crude reaction mixture unless otherwise noted.

7.1 Alkanes obtained by alcohols deoxygenation

Ethylbenzene (2a)[7]

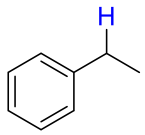

2a (30 min, >98%)

^{1}H NMR (400 MHz, CD_2Cl_2) δ 7.31 – 7.27 (m, 2H), 7.24 – 7.12 (m, 3H), 2.66 (q, *J* = 7.6 Hz, 2H), 1.25 (t, *J* = 7.6 Hz, 3H).

^{13}C NMR (101 MHz, CD_2Cl_2) δ 145.00, 128.85, 128.42, 126.12, 29.44, 16.12.

Diphenylmethane (2b)[7]

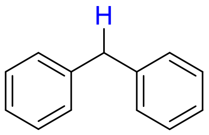

2b (30 min, >98%)

^{1}H NMR (400 MHz, CD_2Cl_2) δ 7.32 – 7.28 (m, 4H), 7.25 – 7.17 (m, 6H), 3.99 (s, 2H).

^{13}C NMR (101 MHz, CD_2Cl_2) δ 142.00, 129.42, 129.02, 127.39, 42.48.

Triphenylmethane (2c)[8]

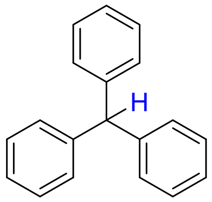

2c (5 min, r.t., >98%)

^{1}H NMR (400 MHz, CD_2Cl_2) δ 7.36 – 7.29 (m, 6H), 7.28 – 7.21 (m, 3H), 7.19 – 7.13 (m, 6H), 5.58 (s, 1H).

^{13}C NMR (101 MHz, CD_2Cl_2) δ 144.60, 129.96, 128.90, 127.41, 57.46.

1-Chloro-4-ethylbenzene (2d)[7]

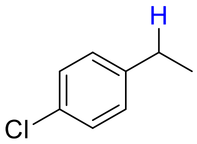

2d (1 h, 94%)

^{1}H NMR (400 MHz, CD_2Cl_2) δ 7.29 – 7.23 (m, 2H), 7.19 – 7.12 (m, 2H), 2.63 (q, *J* = 7.6 Hz, 2H), 1.22 (t, *J* = 7.6 Hz, 3H).

^{13}C NMR (101 MHz, CD_2Cl_2) δ 143.55, 131.68, 129.89, 128.86, 28.81, 15.92.

1-Ethyl-4-methoxybenzene (2e)[7]

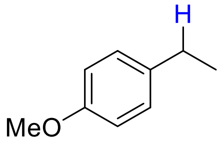

2e (10 min, >98%)

^{1}H NMR (400 MHz, CD_2Cl_2) δ 7.12 (d, *J* = 8.6 Hz, 2H), 6.85 – 6.78 (m, 3H), 3.77 (s, 3H), 2.60 (q, *J* = 7.6 Hz, 2H), 1.21 (t, *J* = 7.6 Hz, 3H).

^{13}C NMR (101 MHz, CD_2Cl_2) δ 158.37, 138.24, 129.26, 114.25, 55.75, 28.54, 16.36.

1-Ethyl-4-(trifluoromethyl)benzene (2f)[7]

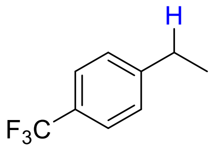

2f (5 h, 59%)

^{1}H NMR (400 MHz, CD_2Cl_2) δ 7.55 (d, *J* = 7.7 Hz, 2H), 7.34 (d, *J* = 7.9 Hz, 2H), 2.72 (q, *J* = 7.7 Hz, 2H), 1.26 (t, *J* = 7.7 Hz, 3H).

^{13}C NMR (101 MHz, CD_2Cl_2) δ 149.31, 128.86, 125.74 (q, *J* = 3.8 Hz), 29.34, 15.70.

^{19}F NMR (376 MHz, CD_2Cl_2) δ -62.56.

Adamantane (2g)[8]

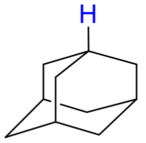

2g (30 min, >98%)

^{1}H NMR (400 MHz, CD_2Cl_2) δ 1.87 (br s, 4H), 1.78 – 1.76 (m, 12H).

^{13}C NMR (101 MHz, CD_2Cl_2) δ 38.35, 29.13.

Methylcyclohexane (2h)[9]

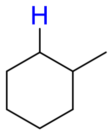

2h (22 h, 94%)

^{1}H NMR (400 MHz, CD_2Cl_2) δ 1.73 – 1.58 (m, 5H), 1.44 – 1.04 (m, 4H), 0.96 – 0.79 (m, 5H).

^{13}C NMR (101 MHz, CD_2Cl_2) δ 36.04, 33.37, 27.08, 26.96, 23.28.

2,4-Dimethylpentane (2i)[10]

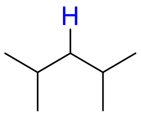

2i (3 h, 98%)

^{1}H NMR (400 MHz, CD_2Cl_2) δ 1.62 (dh, *J* = 13.2, 6.6 Hz, 2H), 1.04 (t, *J* = 7.2 Hz, 2H), 0.86 (d, *J* = 6.6 Hz, 12H).

^{13}C NMR (101 MHz, CD_2Cl_2) δ 49.28, 26.09, 23.18.

7.1.1 ^{1}H and ^{13}C NMR of the isolated diphenylmethane (2b)[7]

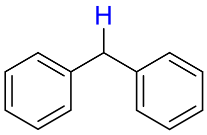

2b

^{1}H NMR (400 MHz, CD_2Cl_2) δ 7.32 (dd, *J* = 8.3, 6.8 Hz, 4H), 7.27 – 7.18 (m, 6H), 4.01 (s, 2H).

^{13}C NMR (101 MHz, CD_2Cl_2) δ 141.99, 129.41, 129.01, 126.61, 42.47.

1,1,1,3,3,3-hexaethyldisiloxane[11]

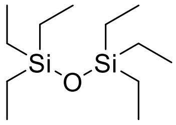

^{1}H NMR (400 MHz, CD_2Cl_2) δ 0.94 (t, *J* = 7.9 Hz, 18H), 0.53 (q, *J* = 7.9 Hz, 12H).

^{13}C NMR (101 MHz, CD_2Cl_2) δ 7.15, 6.93.

GC-MS: retention time (tR) = 7.9 min; m/z 217 (M-Et).

7.1.2 ^{1}H and ^{13}C NMR of the isolated (oxybis(ethane-1,1-diyl))dibenzene 3a[12]

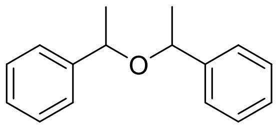

3a (>98%)
Mixture of two isomers 70:10

^{1}H NMR (400 MHz, CD_2Cl_2) for the minor isomer; δ 7.42 – 7.21 (m, 10H), 4.55 (q, *J* = 6.4 Hz, 2H), 1.47 (d, *J* = 6.4 Hz, 6H); for the major isomer δ 7.42 – 7.21 (m, 10H), 4.27 (q, *J* = 6.5 Hz, 2H), 1.37 (d, *J* = 6.5 Hz, 6H).

^{13}C NMR (101 MHz, CD_2Cl_2) for the minor isomer; δ 144.98, 128.73, 127.64, 126.74, 74.87, 23.33. For the major isomer; δ 144.89, 128.94, 127.83, 126.83, 75.18, 25.02.

7.2 Alkanes obtained by ketones deoxygenation

Ethylbenzene (2k)[7]

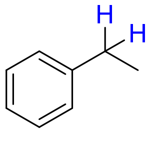

2k (30 min, >98%)

^{1}H NMR (400 MHz, CD_2Cl_2) δ 7.31 – 7.27 (m, 2H), 7.24 – 7.14 (m, 3H), 2.66 (q, *J* = 7.6 Hz, 2H), 1.25 (t, *J* = 7.6 Hz, 3H).

^{13}C NMR (101 MHz, CD_2Cl_2) δ 145.02, 128.87, 128.44, 127.43, 29.47, 16.14.

Diphenylmethane (2l)[7]

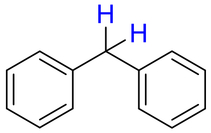

2l (2 h, >98%)

^{1}H NMR (400 MHz, CD_2Cl_2) δ 7.34 – 7.27 (m, 4H), 7.25 – 7.19 (m, 6H), 4.00 (s, 2H).

^{13}C NMR (101 MHz, CD_2Cl_2) δ 142.04, 129.46, 129.06, 127.44, 42.53.

Butylbenzene (2m)[13]

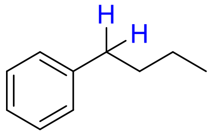

2m (1 h, >98%)

^{1}H NMR (400 MHz, CD_2Cl_2) δ 7.31 – 7.24 (m, 2H), 7.23 – 7.14 (m, 3H), 2.63 (t, *J* = 7.6 Hz, 2H), 1.66 – 1.56 (m, 2H), 1.38 (h, *J* = 7.3 Hz, 2H), 0.95 (t, *J* = 7.4 Hz, 3H).

^{13}C NMR (101 MHz, CD_2Cl_2) δ 143.68, 129.01, 128.79, 126.12, 36.25, 34.42, 23.03, 14.36.

1-Ethyl-4-methoxybenzene (2n)[7]

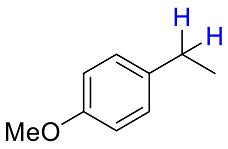

2n (20 h, 96%)

^{1}H NMR (400 MHz, CD_2Cl_2) δ 7.12 (d, *J* = 8.6 Hz, 2H), 6.83 (d, *J* = 8.7 Hz, 2H), 3.78 (s, 3H), 2.60 (q, *J* = 7.6 Hz, 2H), 1.21 (t, *J* = 7.6 Hz, 3H).

^{13}C NMR (101 MHz, CD_2Cl_2) δ 158.37, 137.04, 129.27, 114.25, 55.75, 28.55, 16.38.

1-Ethyl-4-fluorobenzene (2o)[7]

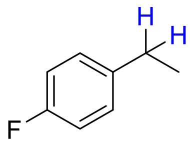

2o (30 min, >98%)

^{1}H NMR (400 MHz, CD_2Cl_2) δ 7.17 (dd, J = 8.5, 5.6 Hz, 2H), 7.03 – 6.94 (m, 2H), 2.63 (q, J = 7.6 Hz, 2H), 1.22 (t, J = 7.6 Hz, 3H).

^{13}C NMR (101 MHz, CD_2Cl_2) δ 160.58 (d, $^1J_{FC}$ = 241.3 Hz), 140.71 (d, $^4J_{FC}$ = 3.2 Hz), 129.77 (d, $^3J_{FC}$ = 7.8 Hz), 115.35 (d, $^2J_{FC}$ = 21.05 Hz), 28.68, 16.22.

^{19}F NMR (376 MHz, CD_2Cl_2) δ -119.04.

1-Chloro-4-ethylbenzene (2p)[7]

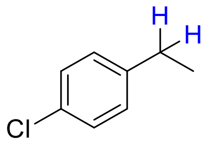

2p (30 min, >98%)

^{1}H NMR (400 MHz, CD_2Cl_2) δ 7.26 (d, J = 8.4 Hz, 2H), 7.16 (d, J = 8.5 Hz, 2H), 2.63 (q, J = 7.6 Hz, 2H), 1.22 (t, J = 7.6 Hz, 3H).

^{13}C NMR (101 MHz, CD_2Cl_2) δ 143.57, 131.72, 129.91, 128.89, 28.84, 15.95.

1-Ethyl-4-iodobenzene (2q)[7]

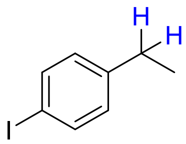

2q, (30 min, >98%)

^{1}H NMR (400 MHz, CD_2Cl_2) δ 7.61 (d, J = 8.3 Hz, 2H), 6.98 (d, J = 8.3 Hz, 2H), 2.60 (q, J = 7.6 Hz, 2H), 1.22 (t, J = 7.6 Hz, 3H).

^{13}C NMR (101 MHz, CD_2Cl_2) δ 144.72, 137.90, 130.69, 90.89, 28.99, 15.84.

1-Bromo-2-ethylbenzene (2r)[14]

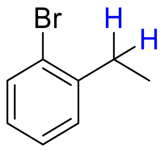

2r (5 min, r.t., >98%)

^{1}H NMR (400 MHz, CD_2Cl_2) δ 7.53 (d, J = 7.9 Hz, 1H), 7.26 (d, J = 3.9 Hz, 2H), 7.10 – 7.02 (m, 1H), 2.77 (q, J = 7.6 Hz, 2H), 1.23 (t, J = 7.6 Hz, 3H).

^{13}C NMR (101 MHz, CD_2Cl_2) δ 144.00, 133.22, 130.20, 128.19, 128.00, 124.77, 29.95, 14.67.

(2-Bromoethyl)benzene (2s)[15]

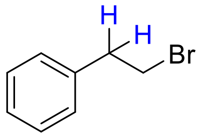

2s (1 h, >98%)

^{1}H NMR (400 MHz, CD_2Cl_2) δ 7.41 – 7.21 (m, 5H), 3.60 (t, J = 7.5 Hz, 2H), 3.17 (t, J = 7.5 Hz, 2H).

^{13}C NMR (101 MHz, CD_2Cl_2) δ 139.69, 129.29, 129.14, 127.44, 39.90, 33.86.

1-Ethyl-4-(trifluoromethyl)benzene (2t)[7]

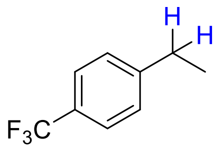

2t (1 h, 87%)

^{1}H NMR (400 MHz, CD_2Cl_2) δ 7.55 (d, J = 8.1 Hz, 2H), 7.34 (d, J = 8.0 Hz, 2H), 2.72 (q, J = 7.6 Hz, 2H), 1.26 (t, J = 7.6 Hz, 3H).

^{13}C NMR (101 MHz, CD_2Cl_2) δ 149.32, 128.87, 125.76 (q, J = 3.8 Hz), 29.36, 15.72.

^{19}F NMR (376 MHz, CD_2Cl_2) δ -62.57.

1-Ethyl-4-nitrobenzene (2u)[16]

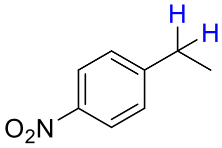

2u (22 h, 39%)

1u was fully converted instantly at r.t. to 17% of **2u** and 83% of the silylated product **2u'**.

^{1}H NMR (400 MHz, CD_2Cl_2) δ 8.13 (d, *J* = 8.7 Hz, 2H), 7.37 (d, *J* = 8.7 Hz, 2H), 2.76 (q, *J* = 7.6 Hz, 2H), 1.27 (t, *J* = 7.6 Hz, 3H).

1,3-Dimethylcyclohexane (2y)[17]

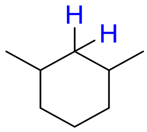

2y (1 h, >98%)

^{1}H NMR (400 MHz, CD_2Cl_2) δ 1.74 – 1.58 (m, 4H), 1.53 – 1.12 (m, 5H), 0.87 (d, *J* = 6.6 Hz, 6H).

^{13}C NMR (101 MHz, CD_2Cl_2) δ 45.10, 35.66, 33.44, 27.09, 23.34.

7.3 Alkanes obtained by Hydrodehalogenation of alkyl halides using TMDS

Triphenylmethane (2aa)[8]

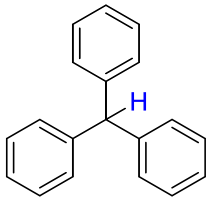

2aa (5 min, r.t., >98%)

^{1}H NMR (400 MHz, CD_2Cl_2) δ 7.34 – 7.30 (m, 6H), 7.27 – 7.23 (m, 3H), 7.18 – 7.16 (m, 6H), 5.59 (s, 1H).

^{13}C NMR (101 MHz, CD_2Cl_2) δ 144.62, 129.98, 128.92, 126.92, 57.48.

Diphenylmethane (2ac)[7]

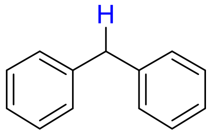

2ac (20 min, r.t., >98%)

^{1}H NMR (400 MHz, CD_2Cl_2) δ 7.33 – 7.29 (m, 4H), 7.26 – 7.18 (m, 6H), 4.00 (s, 2H).

^{13}C NMR (101 MHz, CD_2Cl_2) δ 142.02, 129.44, 129.04, 126.64, 42.51.

Diphenylmethane (2ad)[7]

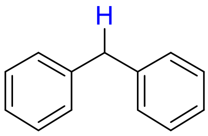

2ad (10 min, 80 °C, >98%)

^{1}H NMR (400 MHz, CD_2Cl_2) δ 7.33 – 7.28 (m, 4H), 7.23 – 7.19 (m, 6H), 4.00 (s, 2H).

^{13}C NMR (101 MHz, CD_2Cl_2) δ 142.01, 129.43, 129.03, 126.63, 42.50.

Ethylbenzene (2ae)[7]

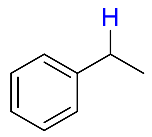

2ae (6 h, 80 °C, 98%)

^{1}H NMR (400 MHz, CD_2Cl_2) δ 7.31 – 7.27 (m, 2H), 7.25 – 7.13 (m, 3H), 2.66 (q, *J* = 7.6 Hz, 2H), 1.25 (t, *J* = 7.6 Hz, 3H).

^{13}C NMR (101 MHz, CD_2Cl_2) δ 145.00, 128.85, 128.42, 126.12, 29.44, 16.12.

Adamantane (2af)[8]

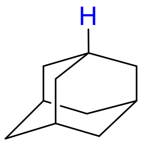

2af (5 h, 120 °C, 95%)

^{1}H NMR (400 MHz, CD_2Cl_2) δ 1.90 – 1.84 (m, 4H), 1.79 – 1.77 (m, 12H).

^{13}C NMR (101 MHz, CD_2Cl_2) δ 38.33, 29.11.

7.4 Alkanes obtained by Hydrodehalogenation of alkyl halides 1ab–1ad using Et_3SiH

Diphenylmethane (2ab)[7]

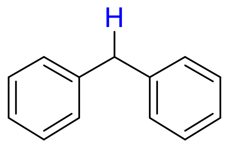

2ab (5 min, r.t., >98%)

^{1}H NMR (400 MHz, CD_2Cl_2) δ 7.35 – 7.26 (m, 4H), 7.26 – 7.15 (m, 6H), 3.99 (s, 2H).

^{13}C NMR (101 MHz, CD_2Cl_2) δ 141.86, 129.27, 128.87, 126.47, 42.34.

Diphenylmethane (2ac)[7]

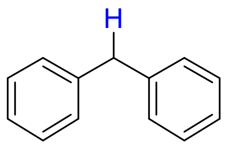

2ac (5 min, r.t., >98%)

^{1}H NMR (400 MHz, CD_2Cl_2) δ 7.35 – 7.27 (m, 4H), 7.27 – 7.16 (m, 6H), 4.00 (s, 2H).

^{13}C NMR (101 MHz, CD_2Cl_2) δ 141.85, 129.27, 128.87, 127.25, 42.34.

Diphenylmethane (2ad)[7]

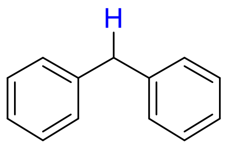

2ad (5 min, 80 °C, >98%)

^{1}H NMR (400 MHz, CD_2Cl_2) δ 7.34 – 7.26 (m, 4H), 7.26 – 7.18 (m, 6H), 4.00 (s, 2H).

^{13}C NMR (101 MHz, CD_2Cl_2) δ 141.84, 129.26, 128.86, 127.24, 42.33.

7.5 NMR data for the regio-chemoselective deoxygenation of 1aj and 1ak

2-phenylethan-1-ol (2aj)[18]

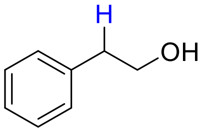

2aj (18 h, 100 °C, >98%)

^{1}H NMR (400 MHz, CD_2Cl_2) δ 7.32 – 7.25 (m, 2H), 7.24 – 7.18 (m, 3H), 3.66 (t, *J* = 7.0 Hz, 2H), 2.87 (t, *J* = 7.0 Hz, 2H).

^{13}C NMR (101 MHz, CD_2Cl_2) δ 139.88, 129.37, 128.67, 126.49, 72.18, 36.70.

2-(3-benzylphenyl)propanoic acid (2ak)[19]

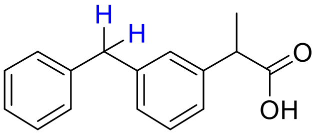

2ak (30 min, 80 °C, >98%)

^{1}H NMR (400 MHz, CD_2Cl_2) δ 11.77 (s, 1H), 7.34 – 7.07 (m, 9H), 3.98 (s, 2H), 3.73 (q, *J* = 7.1 Hz, 1H), 1.47 (d, *J* = 7.2 Hz, 3H).

^{13}C NMR (101 MHz, CD_2Cl_2) δ 182.13, 142.45, 141.49, 140.04, 129.33, 129.30, 128.94, 128.75, 128.56, 126.58, 125.65, 45.83, 42.23, 18.21.

7.6 Silyl ethers obtained by hydrosilylation of ketones (1u, 1v, and 1w)

1,1,3,3-tetramethyl-1-(1-(4-nitrophenyl)ethoxy)disiloxane (2u′)[20]

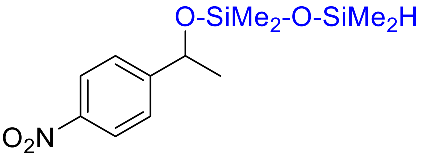

2u' (5 min, r.t., >98%)
mixture of isomers

^{1}H NMR (400 MHz, CD_2Cl_2) δ 8.17 (d, *J* = 8.4 Hz, 2H), 7.53 (d, *J* = 8.6 Hz, 2H), 5.09 (q, *J* = 6.6 Hz, 1H, major isomer), 4.98 (q, *J* = 6.4, Hz, 1H, minor isomer), 4.67 (m, 1H, Si-H of different isomers + unreacted TMDS), 1.46 (d, *J* = 6.5 Hz, 3H), 0.19 (m, 16H, CH_3 of two isomers + reacted and unreacted TMDS), 0.14 (s br, 3H), 0.07 (s br, 3H).

^{13}C NMR (101 MHz, CD_2Cl_2) δ 153.48, 152.24, 128.66, 126.15, 126.12, 123.48, 123.45, 123.40, 71.26, 69.45, 28.81, 26.35, 26.00, 14.82, (0.52 – 0.23), -1.05, -1.16, -1.50, -1.58.

4-(1-((1,1,3,3-tetramethyldisiloxaneyl)oxy)ethyl)benzonitrile (2v′)

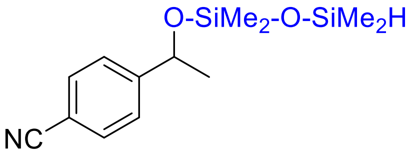

2v' (5 min, r.t., >98%)
mixture of isomers

^{1}H NMR (400 MHz, CD_2Cl_2) δ 7.64 (d, *J* = 8.0 Hz, 2H), 7.48 (d, *J* = 8.0 Hz, 2H), 5.04 (q, *J* = 6.5 Hz, 1H, major isomer), 4.92 (q, *J* = 6.4, Hz, 1H, minor isomer), 4.67 (m, 1H, Si-H of different isomers + unreacted TMDS), 1.44 (d, *J* = 6.5 Hz, 3H), 0.19 (m, 11H, CH_3 of two isomers + reacted and unreacted TMDS), 0.13 (m, 3H), 0.06 (m, 3H).

^{13}C NMR (101 MHz, CD_2Cl_2) δ 152.44, 152.07, 132.94, 132.77, 132.73, 126.73, 126.69, 126.67, 72.02, 70.21, 26.93, 26.57, (1.11 – 0.86), -0.43, -0.55, -0.88, -0.96.

4-(1-((1,1,3,3-tetramethyldisiloxaneyl)oxy)ethyl)pyridine (2w′)

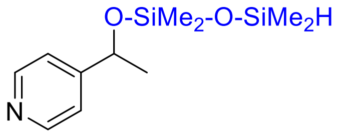

2w' (22 h, 80 °C, >98%)
mixture of two isomers

^{1}H NMR (400 MHz, CD_2Cl_2) δ 8.55 (d, *J* = 6.1 Hz, 2H), 7.37 (d, *J* = 6.3 Hz, 2H), 4.96 (dq, *J* = 47.7, 6.4 Hz, 1H major isomer), 4.90 (q, *J* = 6.7, Hz, 1H, minor isomer), 4.68 (m, 1H, Si-H of different isomers + unreacted TMDS), 1.45 (d, *J* = 6.5 Hz, 3H), 0.19 (m, 15H, CH_3 of two isomers + reacted and unreacted TMDS), 0.15 (m, 3H), 0.09 (m, 3H).

^{13}C NMR (101 MHz, CD_2Cl_2) δ 157.35 major, 149.25 minor, 149.06 minor, 121.48 major, 121.41 minor, 71.28 minor, 69.48 major, 26.51 major, 26.16 minor, 0.90 minor, 0.86 major, -0.43 major, -0.55 major, -0.88 minor, -0.96 minor.

8 References

[1] M. I. Lapuh, G. Cormier, S. Chergui, D. J. Aitken, T. Boddaert, *Org. Lett.* **2022**, *24*, 8375–8380.
[2] F. Leroux, L. Garamszegi, M. Schlosser, *J. Fluor. Chem.* **2002**, *117*, 177–180.
[3] S. Shivprakash, G. C. and Reddy, *Synth. Commun.* **2014**, *44*, 600–609.
[4] S. Keess, A. Simonneau, M. Oestreich, *Organometallics* **2015**, *34*, 790–799.
[5] Y. Gan, W. Xu, Y. Liu, *Org. Lett.* **2019**, *21*, 9652–9657.
[6] T. He, H. F. T. Klare, M. Oestreich, *Nature* **2023**, *623*, 538–543.
[7] R. Sun, H.-Y. Guo, S.-S. Ma, Y.-F. Wang, Z.-K. Yu, B.-H. Xu, *Org. Chem. Front.* **2022**, *9*, 1943–1954.
[8] H. Fang, M. Oestreich, *Angew. Chem. Int. Ed.* **2020**, *59*, 11394–11398.
[9] N. G. Léonard, P. J. Chirik, *ACS Catal.* **2018**, *8*, 342–348.
[10] V. Udayakumar, A. Pandurangan, *RSC Adv.* **2015**, *5*, 78719–78727.
[11] A. Simonneau, J. Friebel, M. Oestreich, *Eur. J. Org. Chem.* **2014**, *10*, 2077–2083.
[12] P. K. Sahoo, S. S. Gawali, C. Gunanathan, *ACS Omega* **2018**, *3*, 124–136.
[13] C.-T. Yang, Z.-Q. Zhang, Y.-C. Liu, L. Liu, *Angew. Chem. Int. Ed.* **2011**, *50*, 3904–3907.
[14] S. P. Cummings, T.-N. Le, G. E. Fernandez, L. G. Quiambao, B. J. Stokes, *J. Am. Chem. Soc.* **2016**, *138*, 6107–6110.
[15] Z. Fu, G. Hao, Y. Fu, D. He, X. Tuo, S. Guo, H. Cai, *Org. Chem. Front.* **2020**, *7*, 590–595.
[16] R. Shen, T. Chen, Y. Zhao, R. Qiu, Y. Zhou, S. Yin, X. Wang, M. Goto, L.-B. Han, *J. Am. Chem. Soc.* **2011**, *133*, 17037–17044.
[17] Z. Liu, C.-D. Wu, *Appl. Catal. Gen.* **2024**, *685*, 119901.
[18] M. Szostak, M. Spain, A. J. Eberhart, D. J. Procter, *J. Org. Chem.* **2014**, *79*, 11988–12003.
[19] J. P. G. Rygus, D. G. Hall, *Nat. Commun.* **2023**, *14*, 2563.
[20] V. V. Zuev, D. A. and de Vekki, *Phosphorus Sulfur Silicon Relat. Elem.* **2005**, *180*, 2071–2083.

9 Spectra

9.1 ^{1}H and ^{13}C NMR Spectra of alkanes obtained by alcohols deoxygenation

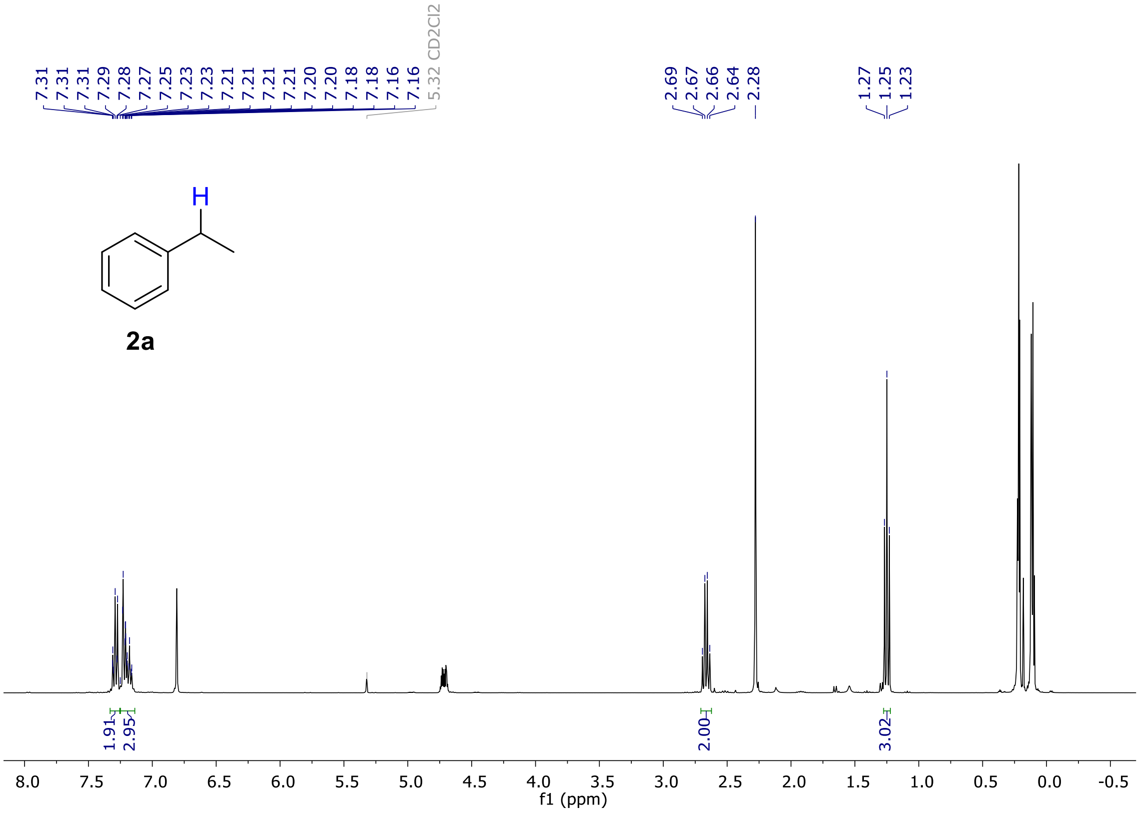

*Figure S19. ^{1}H NMR spectrum of the crude reaction mixture of **2a**.*

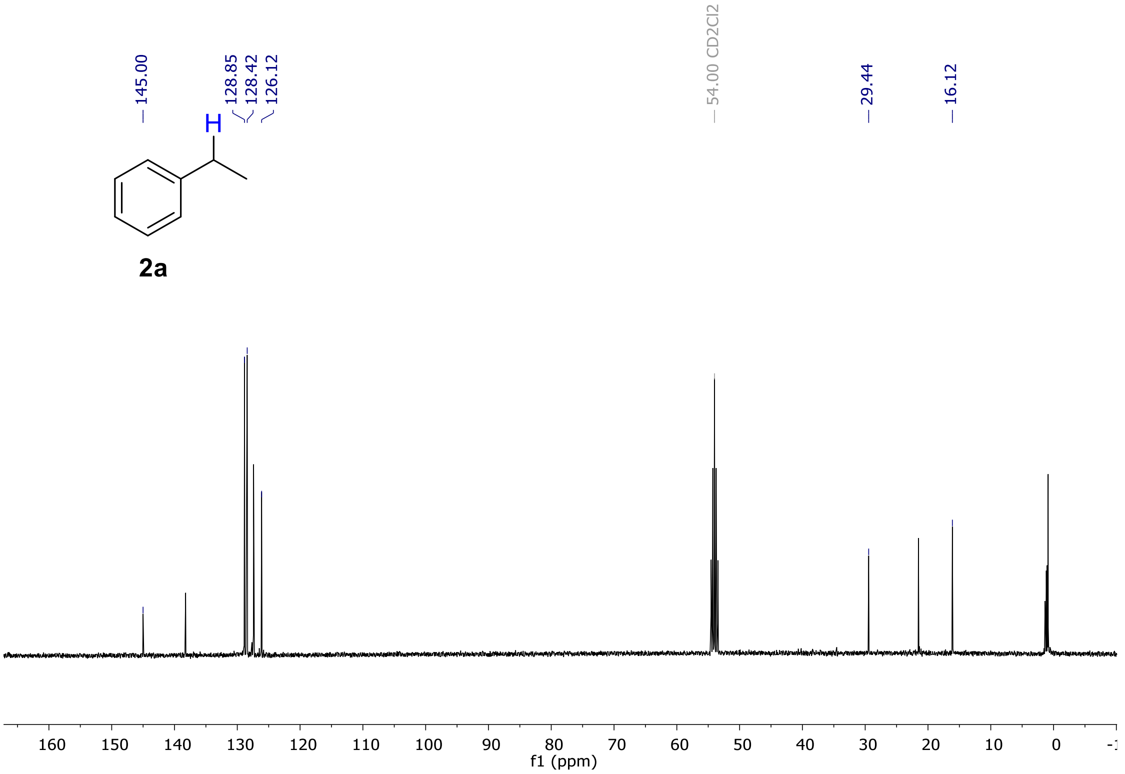

*Figure S20. ^{13}C{^{1}H} NMR spectrum of the crude reaction mixture of **2a**.*

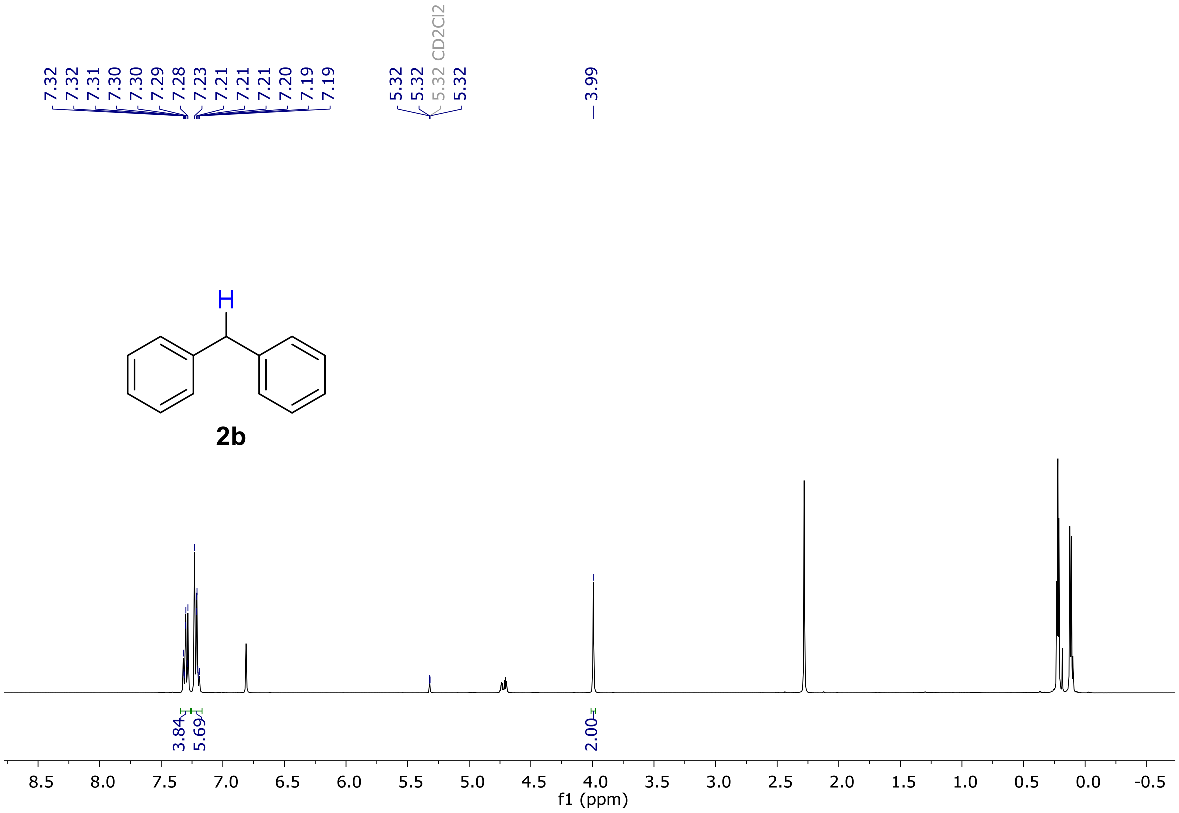

*Figure S21. ^{1}H NMR spectrum of the crude reaction mixture of **2b**.*

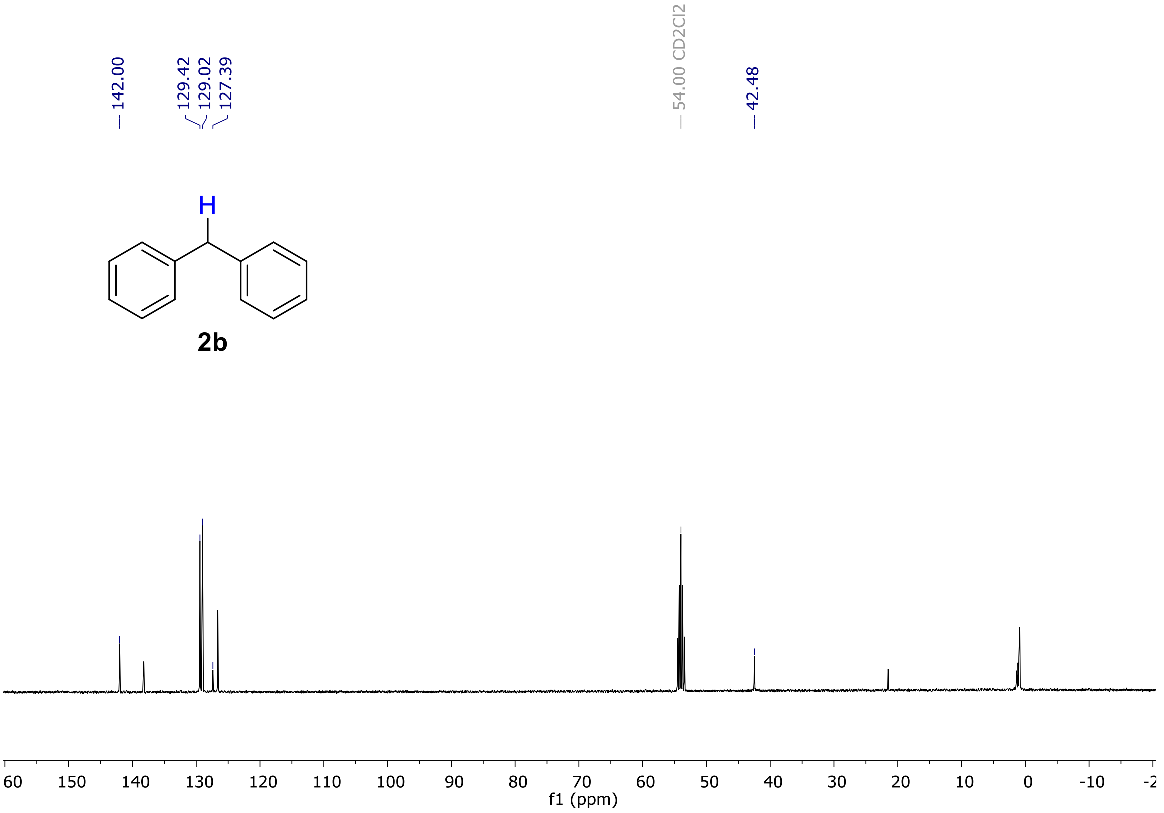

*Figure S22. $^{13}C\{^1H\}$ NMR spectrum of the crude reaction mixture of **2b**.*

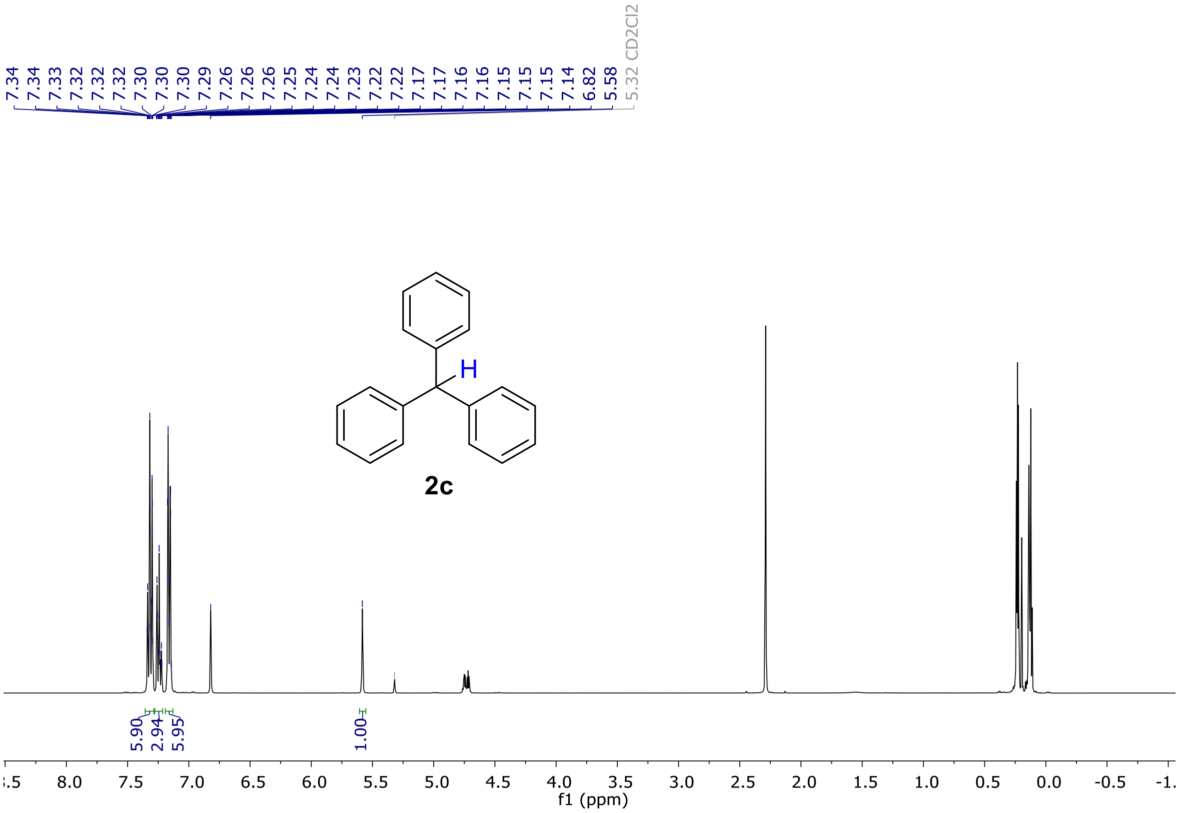

Figure S23. ^{1}H *NMR spectrum of the crude reaction mixture of* ***2c****.*

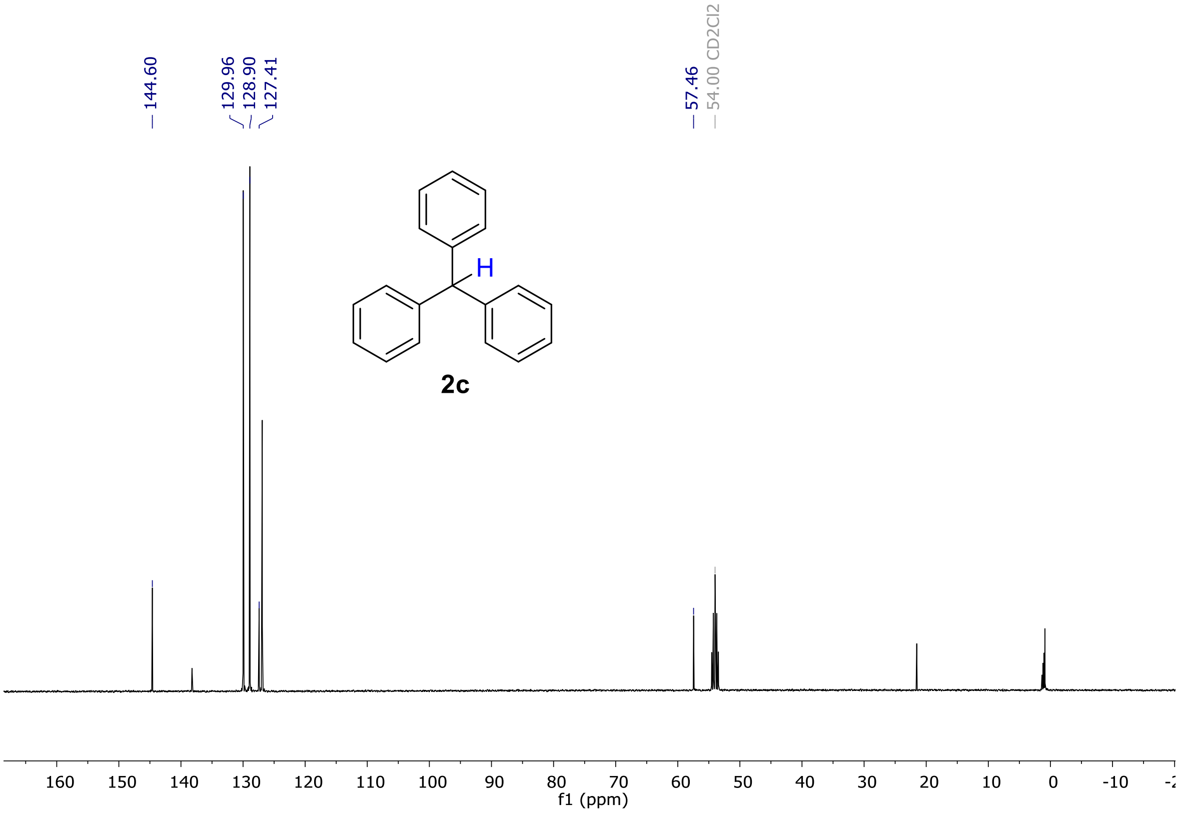

Figure S24. $^{13}C\{^{1}H\}$ *NMR spectrum of the crude reaction mixture of* ***2c****.*

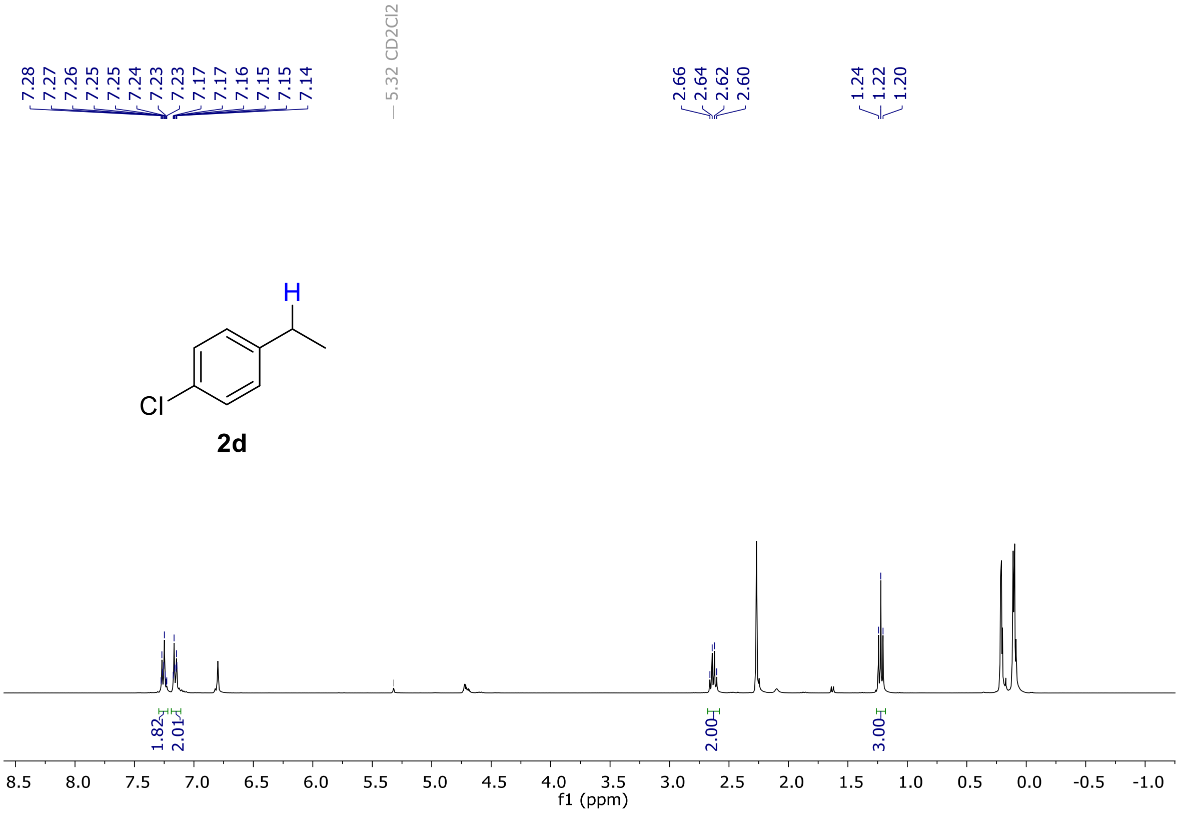

Figure S25. ^{1}H NMR spectrum of the crude reaction mixture of ***2d****.*

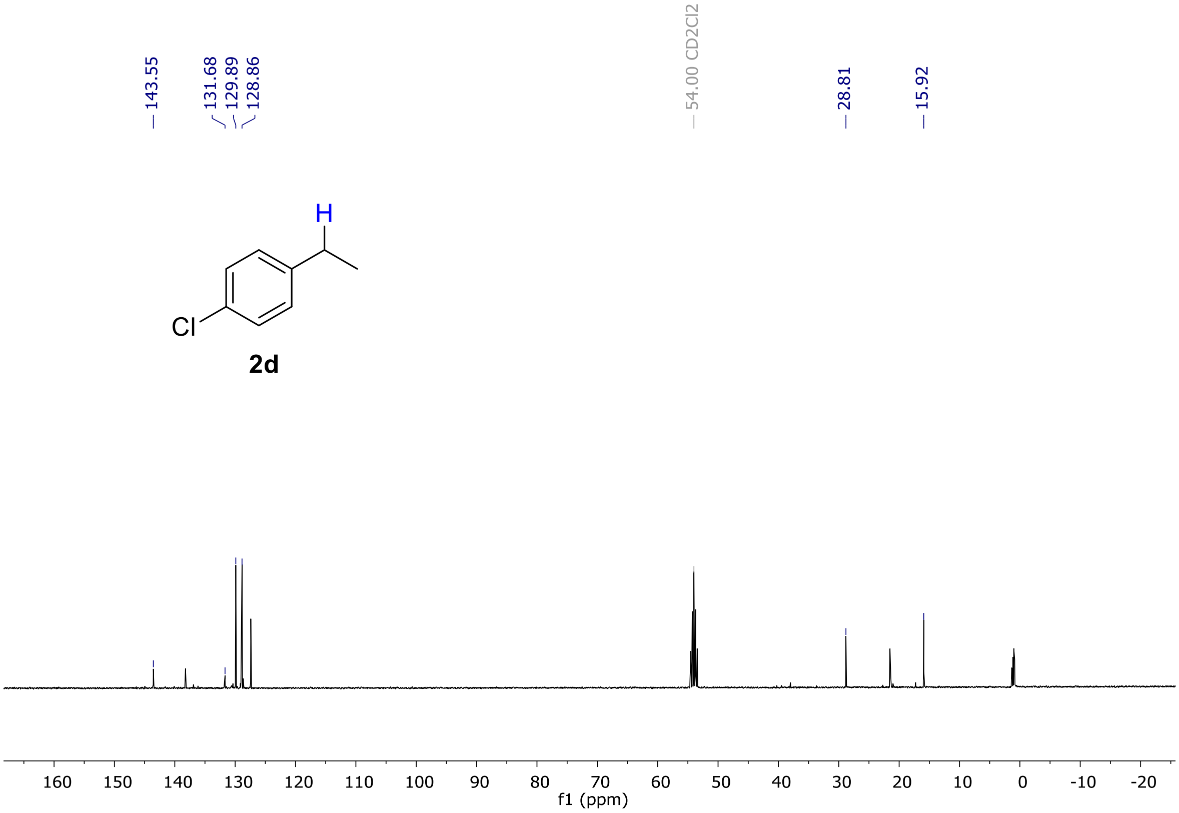

Figure S26. $^{13}C\{^{1}H\}$ NMR spectrum of the crude reaction mixture of ***2d****.*

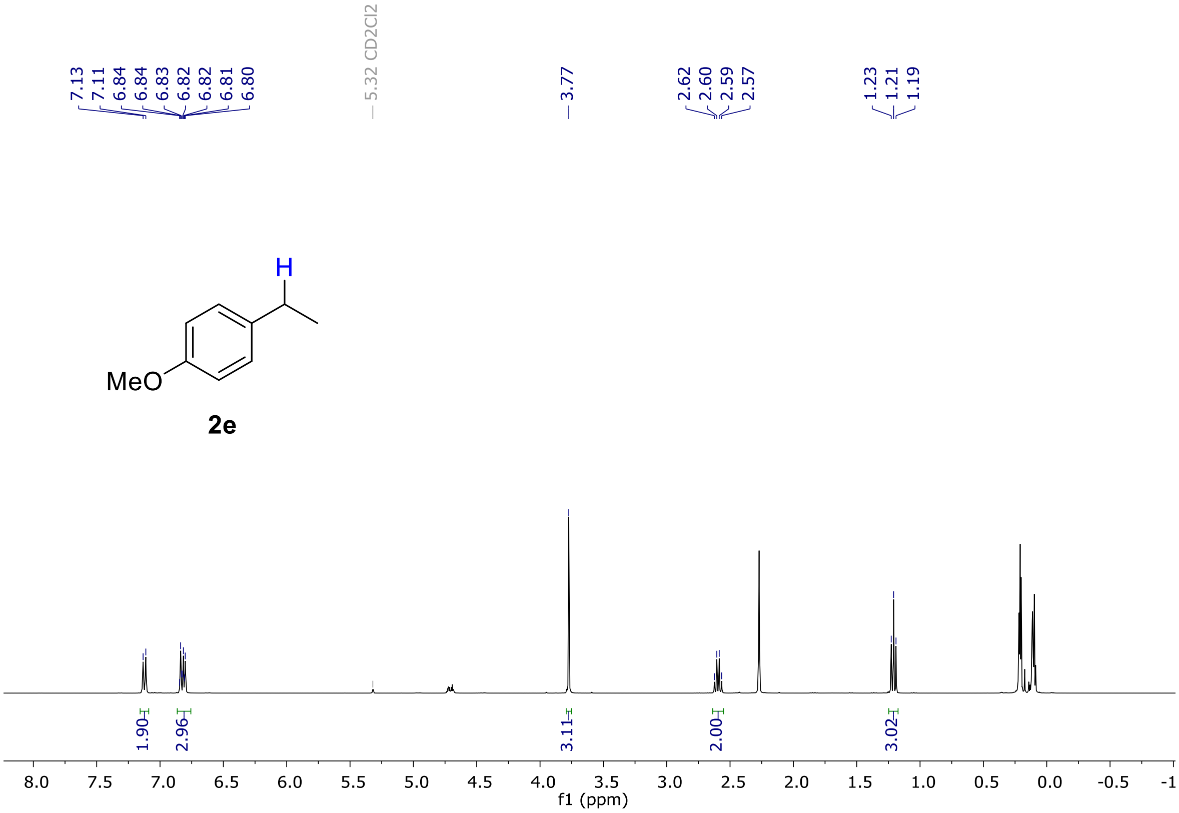

Figure S27. ^{1}H NMR spectrum of the crude reaction mixture of ***2e****.*

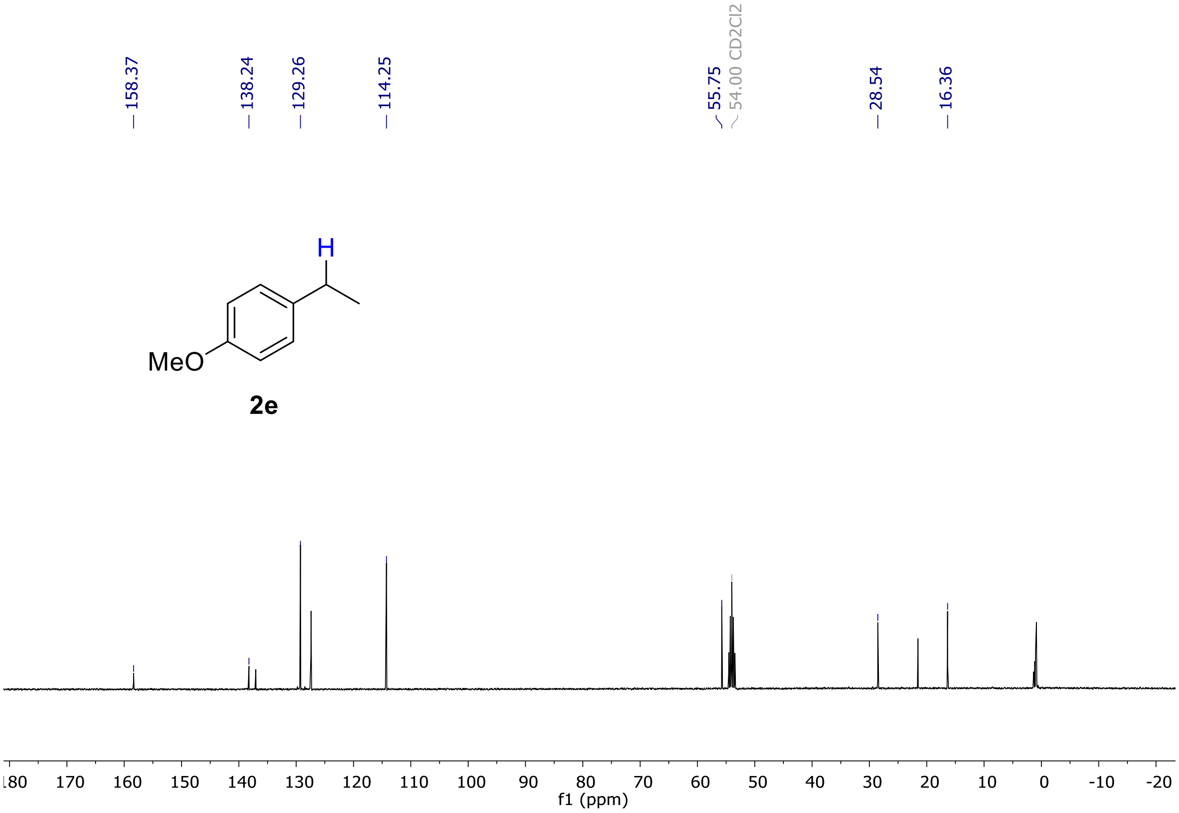

Figure S28. $^{13}C\{^{1}H\}$ NMR spectrum of the crude reaction mixture of ***2e****.*

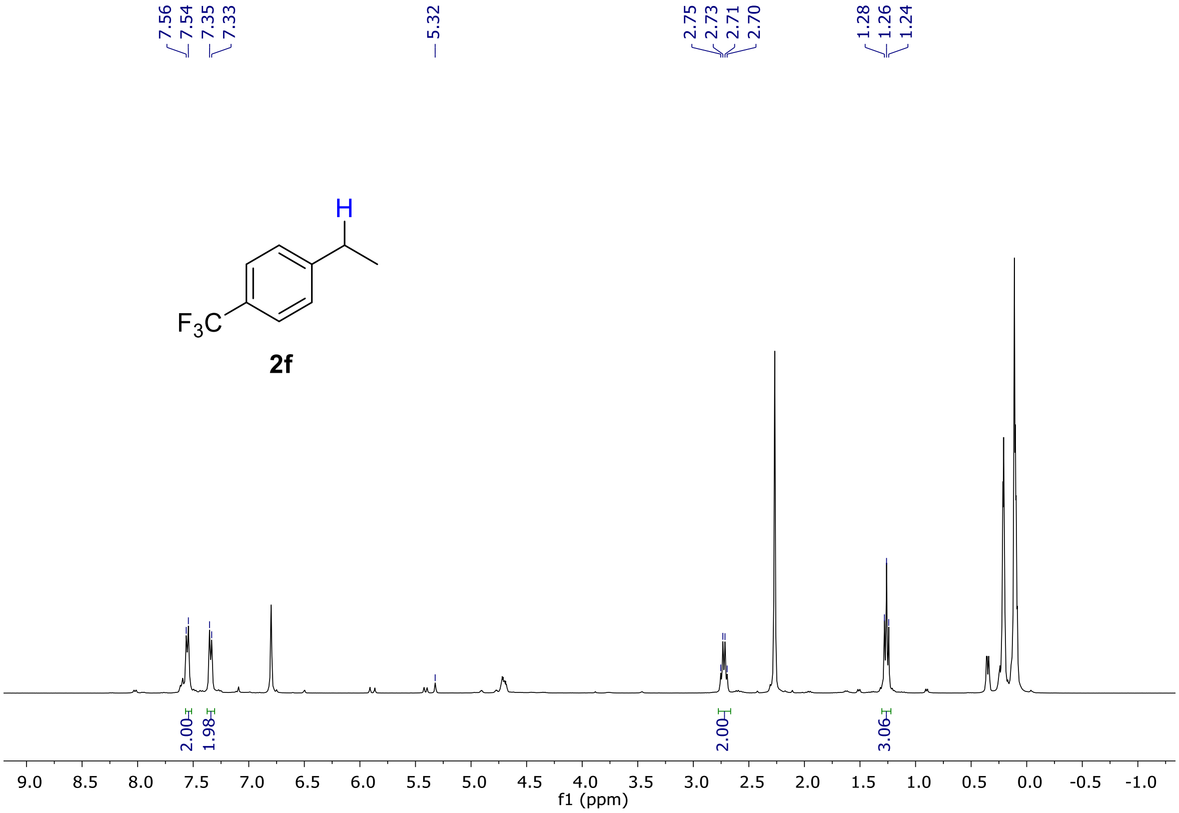

*Figure S29. ^{1}H NMR spectrum of the crude reaction mixture of **2f**.*

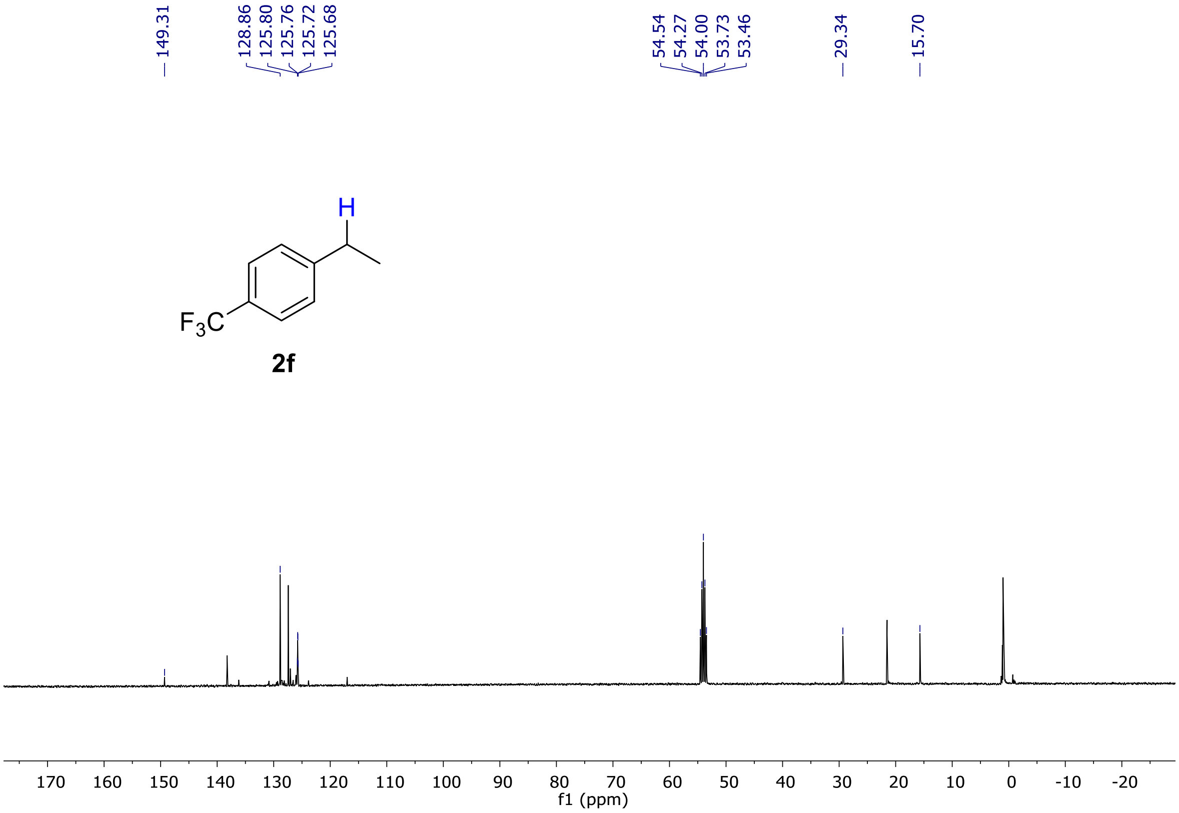

*Figure S30. $^{13}C\{^{1}H\}$ NMR spectrum of the crude reaction mixture of **2f**.*

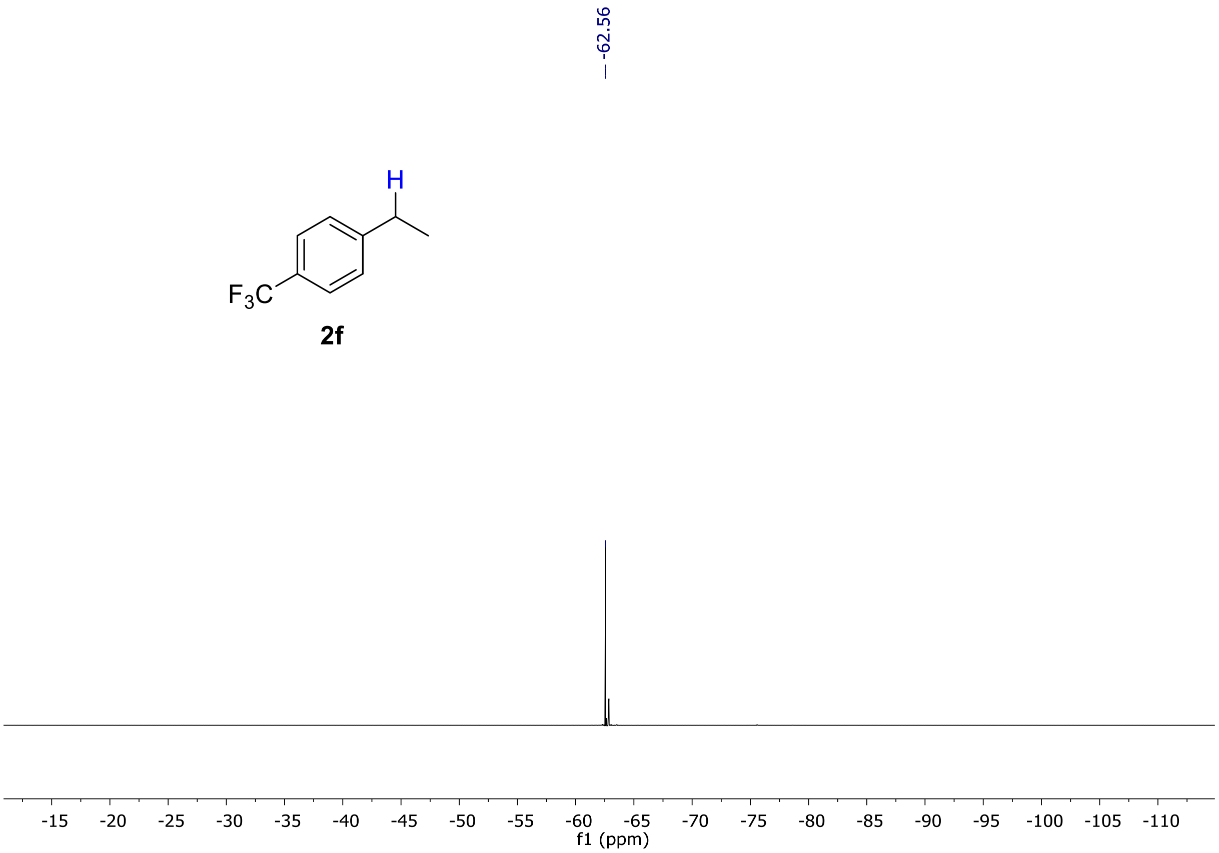

Figure S31. $^{19}F\{^1H\}$ *NMR spectrum of the crude reaction mixture of* ***2f***.

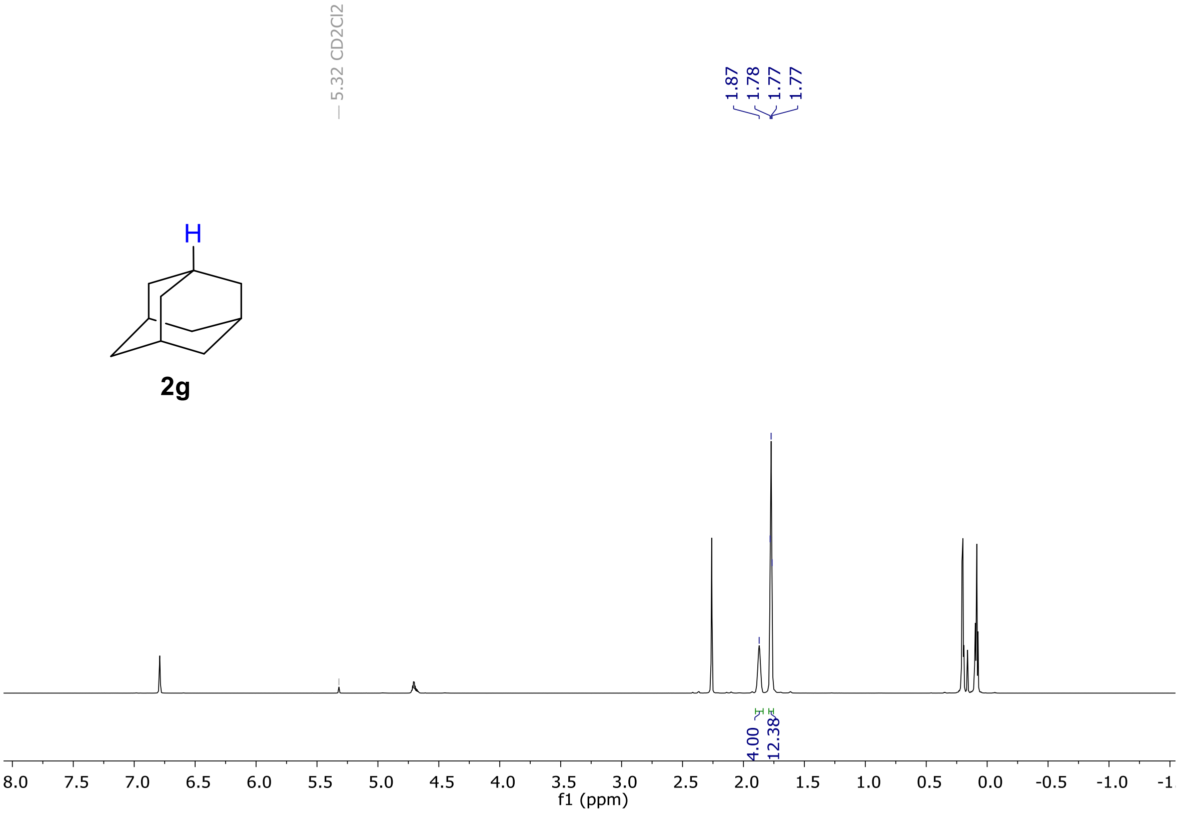

Figure S32. ^{1}H NMR spectrum of the crude reaction mixture of ***2g****.*

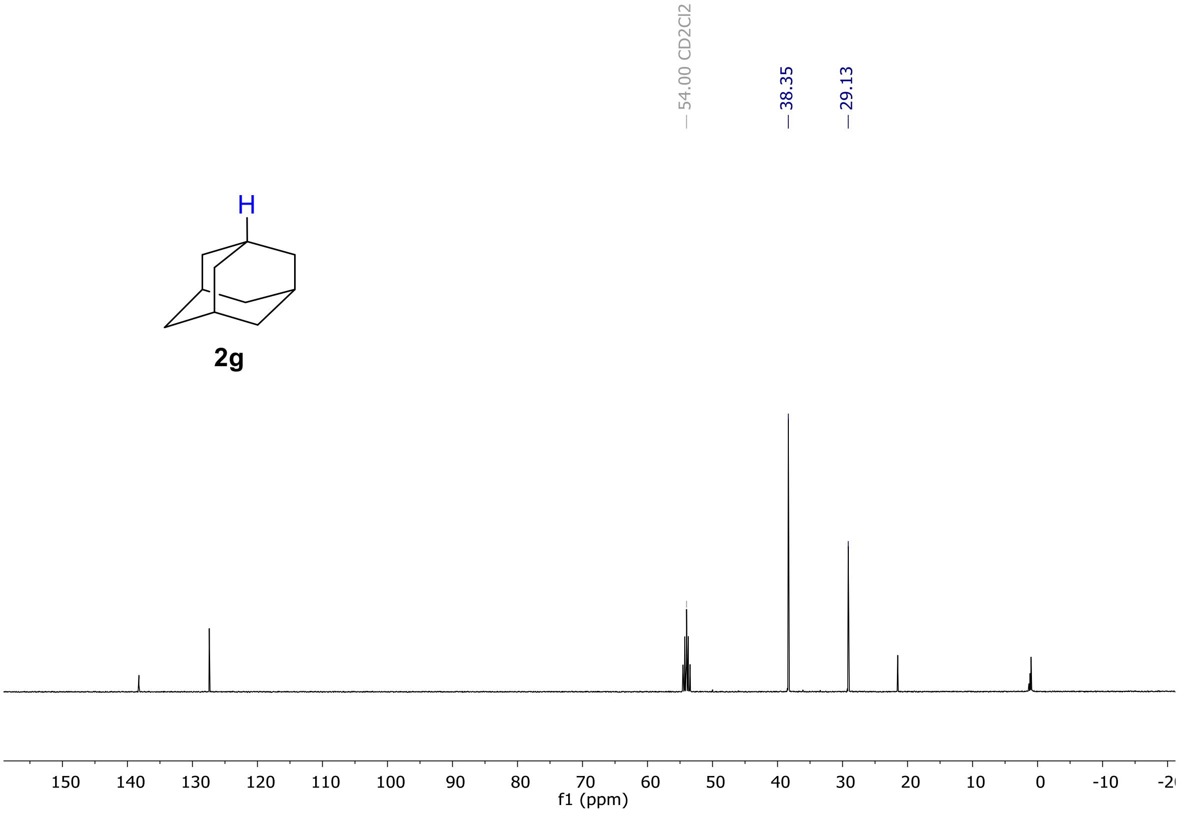

Figure S33. ^{13}C{^{1}H} NMR spectrum of the crude reaction mixture of ***2g****.*

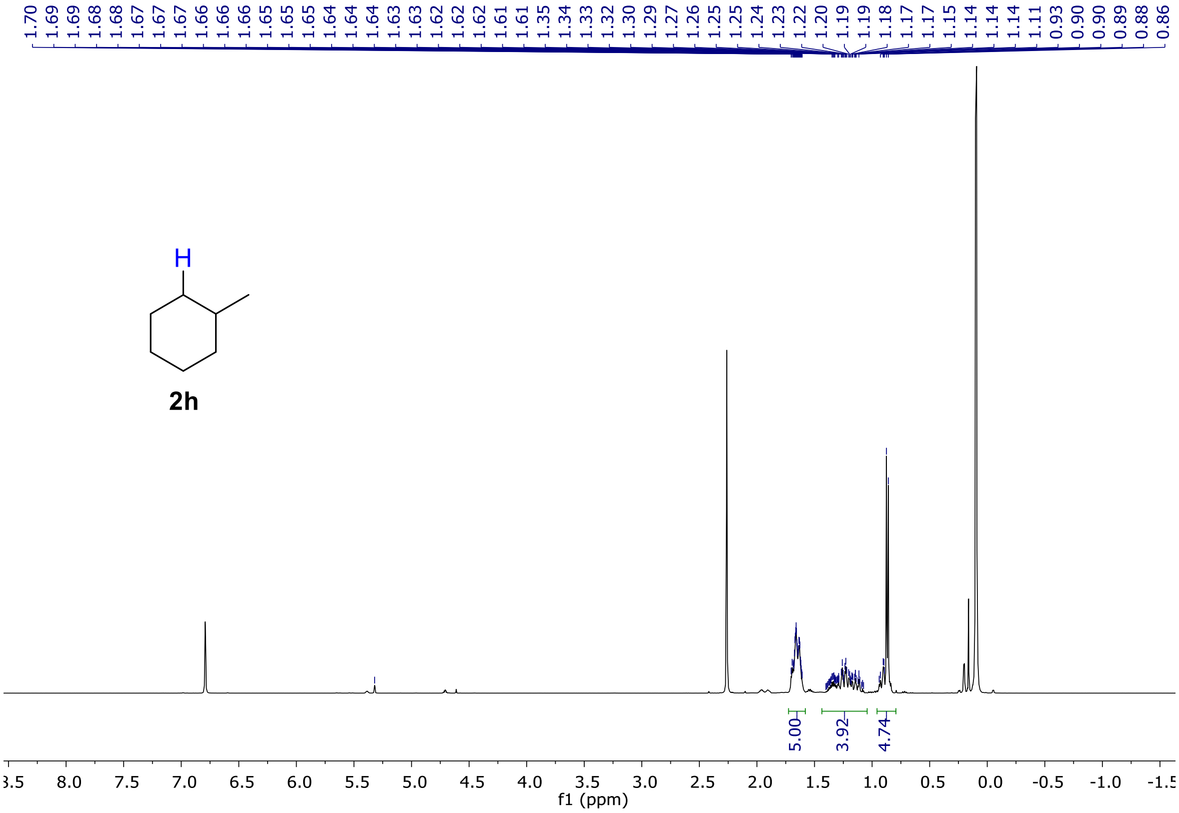

*Figure S34. ^{1}H NMR spectrum of the crude reaction mixture of **2h**.*

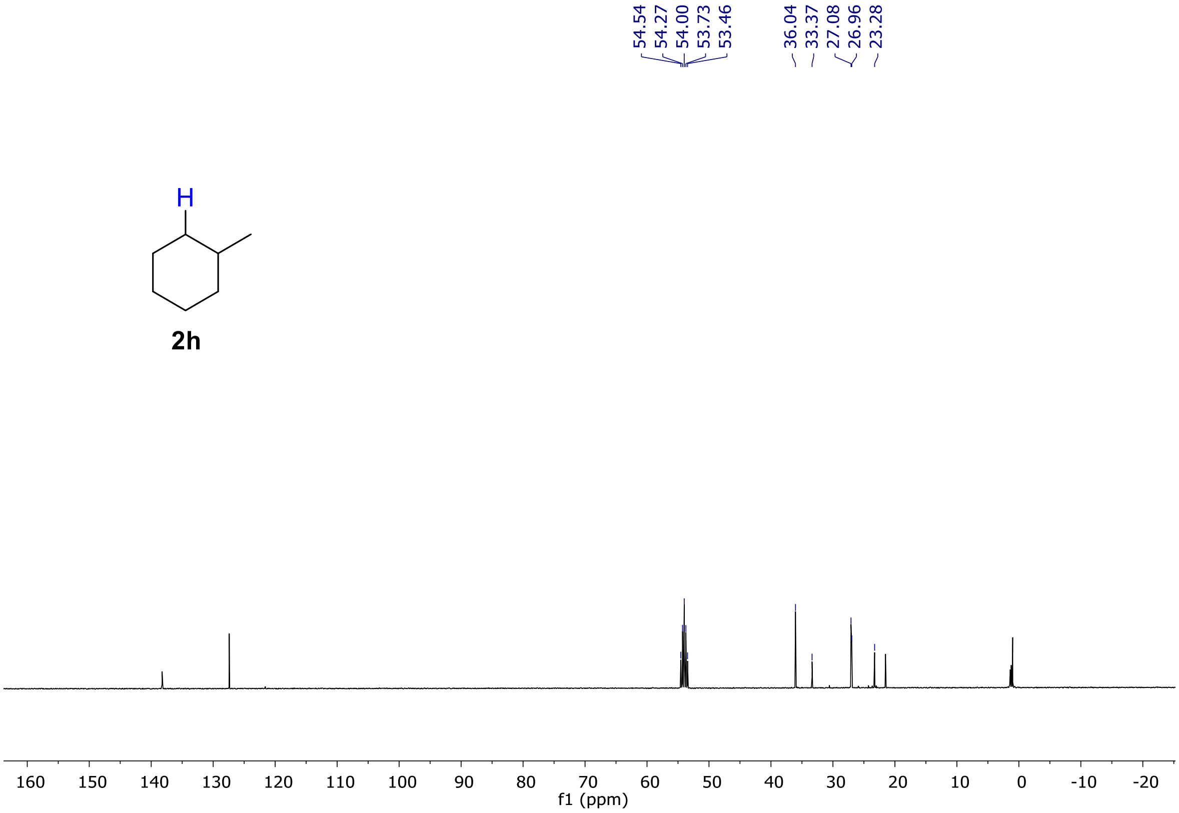

*Figure S35. ^{13}C{^{1}H} NMR spectrum of the crude reaction mixture of **2h**.*

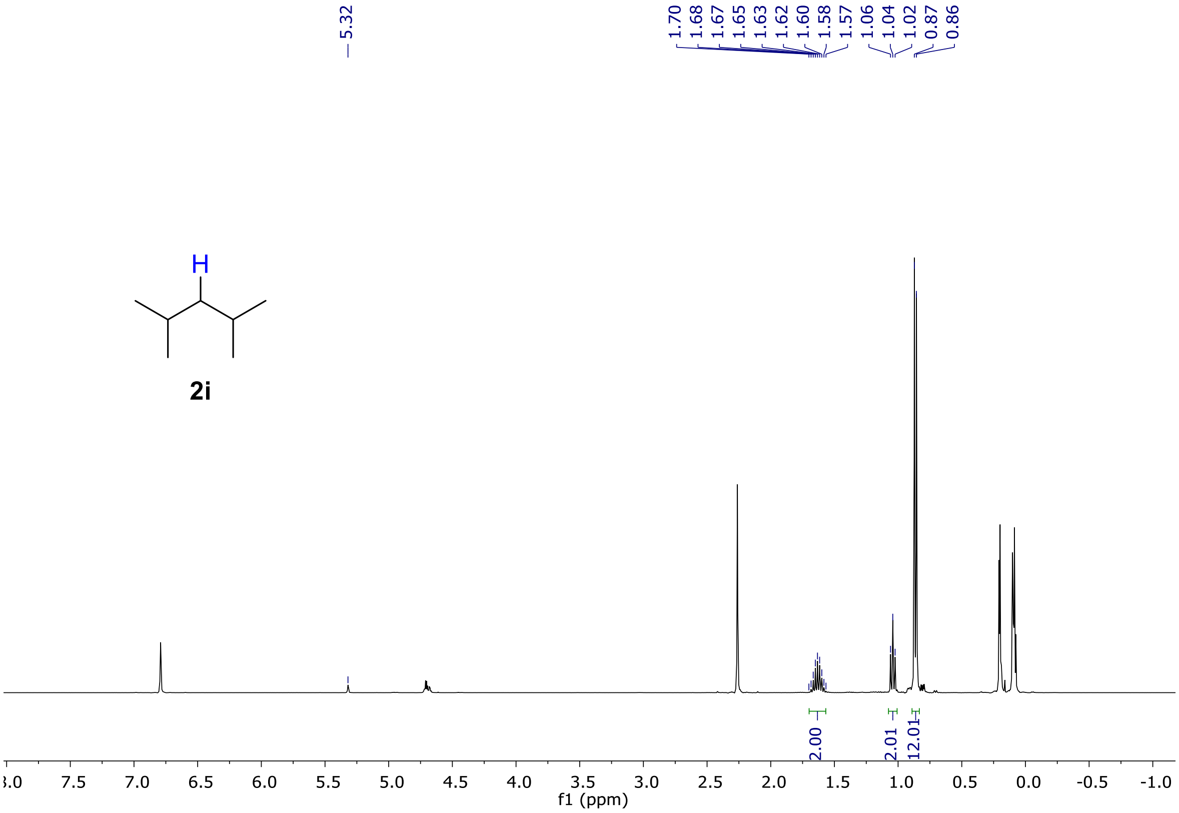

Figure S36.[1]*H NMR spectrum of the crude reaction mixture of* ***2i****.*

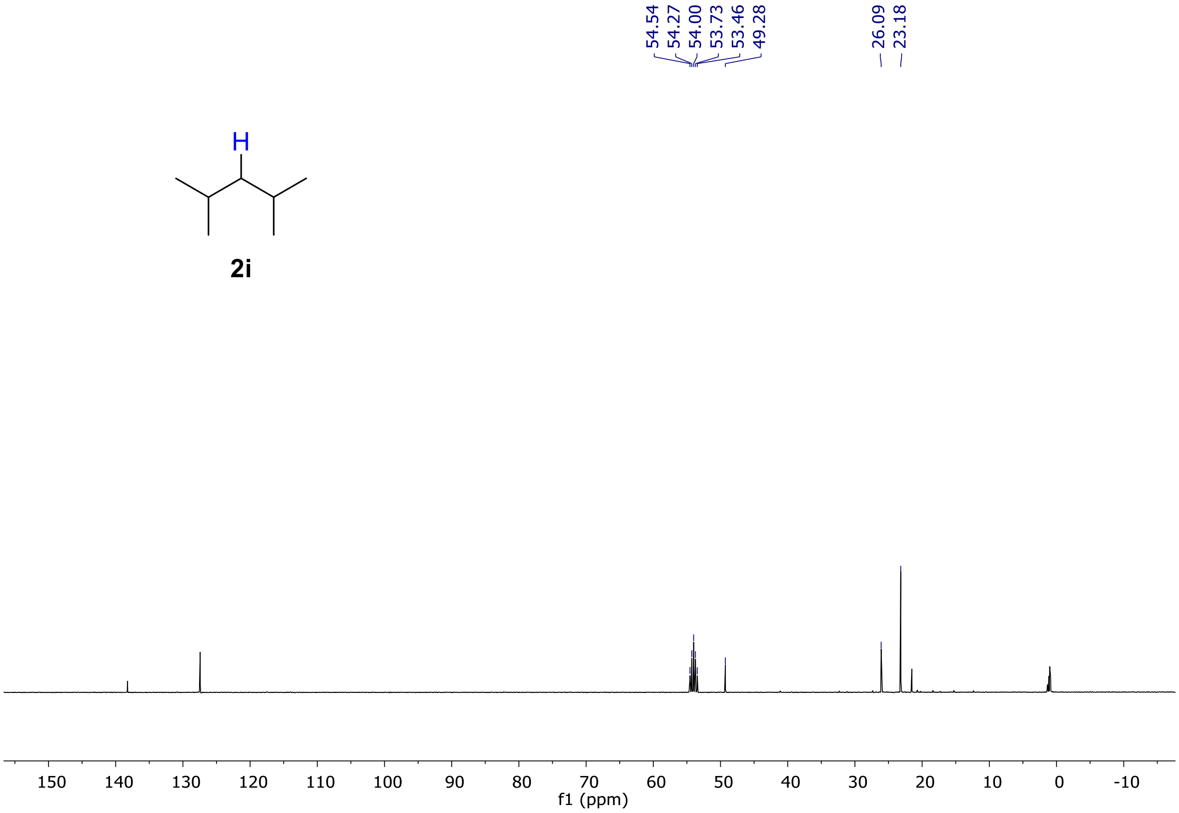

Figure S37. $^{13}C\{^{1}H\}$ *NMR spectrum of the crude reaction mixture of* ***2i****.*

9.1.1 ^{1}H and ^{13}C NMR spectra of the isolated diphenylmethane (2b) using Et_3SiH as hydrosilane

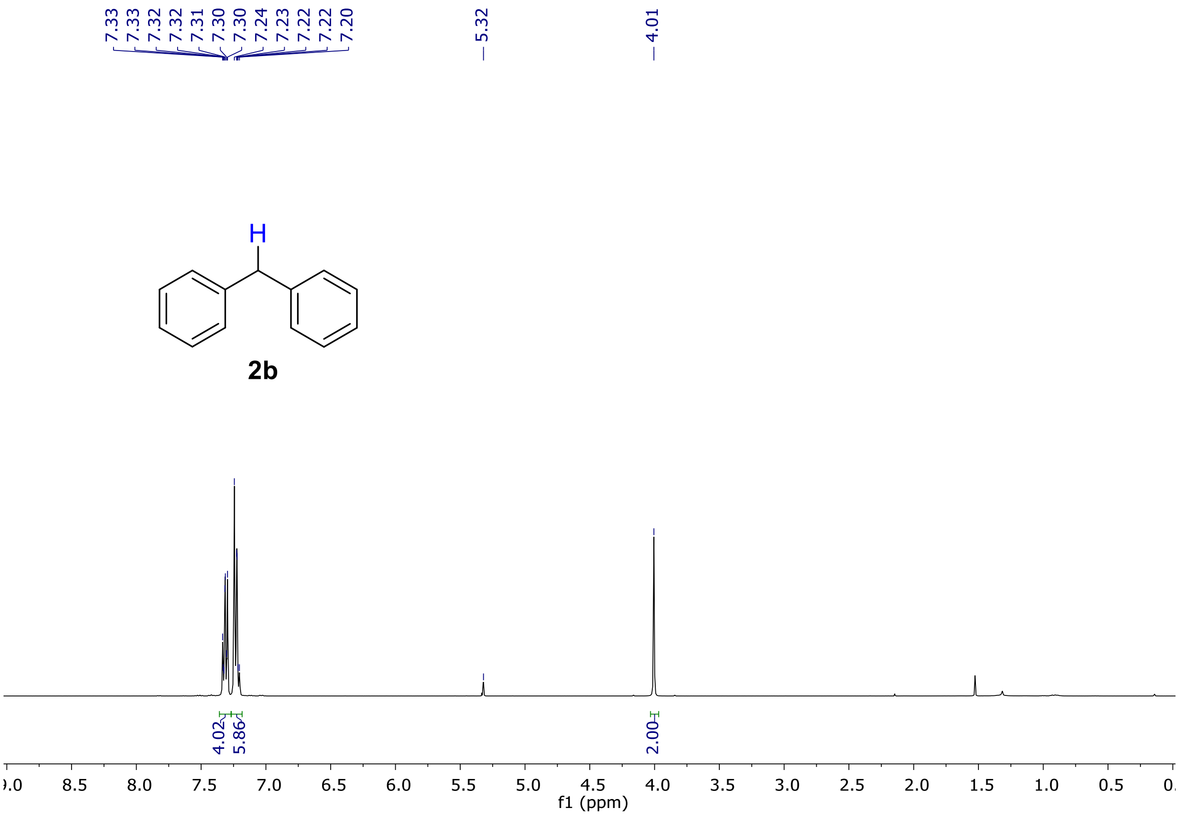

*Figure S38. ^{1}H NMR spectrum of the isolated product **2b**.*

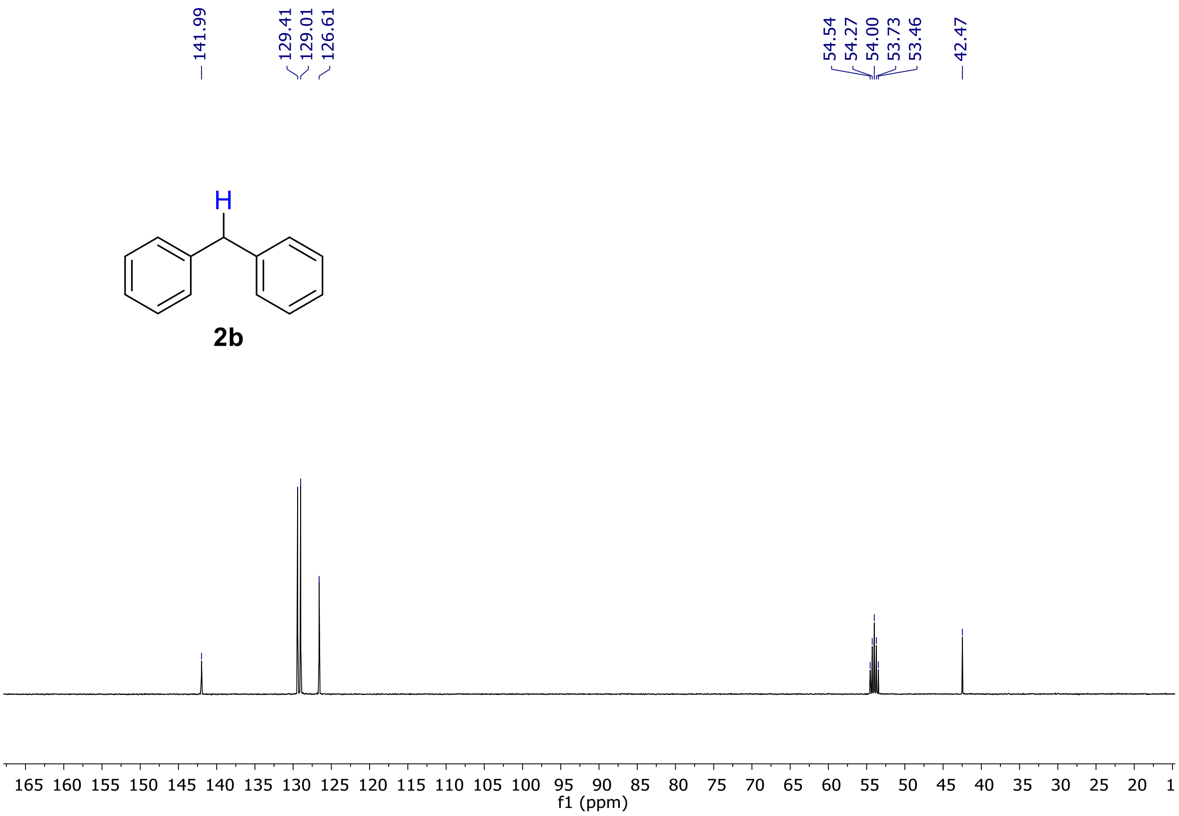

*Figure S39. $^{13}C\{^{1}H\}$ NMR spectrum of the isolated product **2b**.*

9.1.2 ^{1}H and ^{13}C NMR spectra of the isolated diphenylmethane (2b) using TMDS as hydrosilane

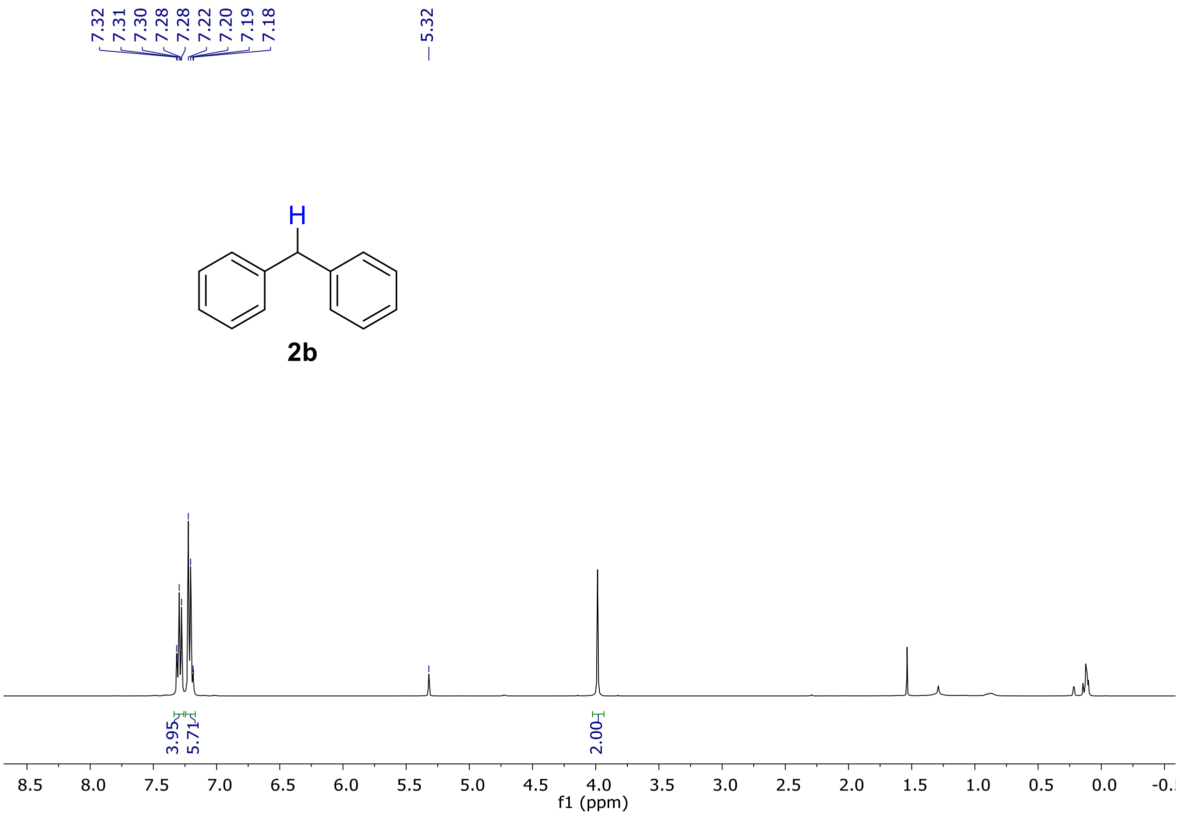

*Figure S40. ^{1}H NMR spectrum of the isolated product **2b**.*

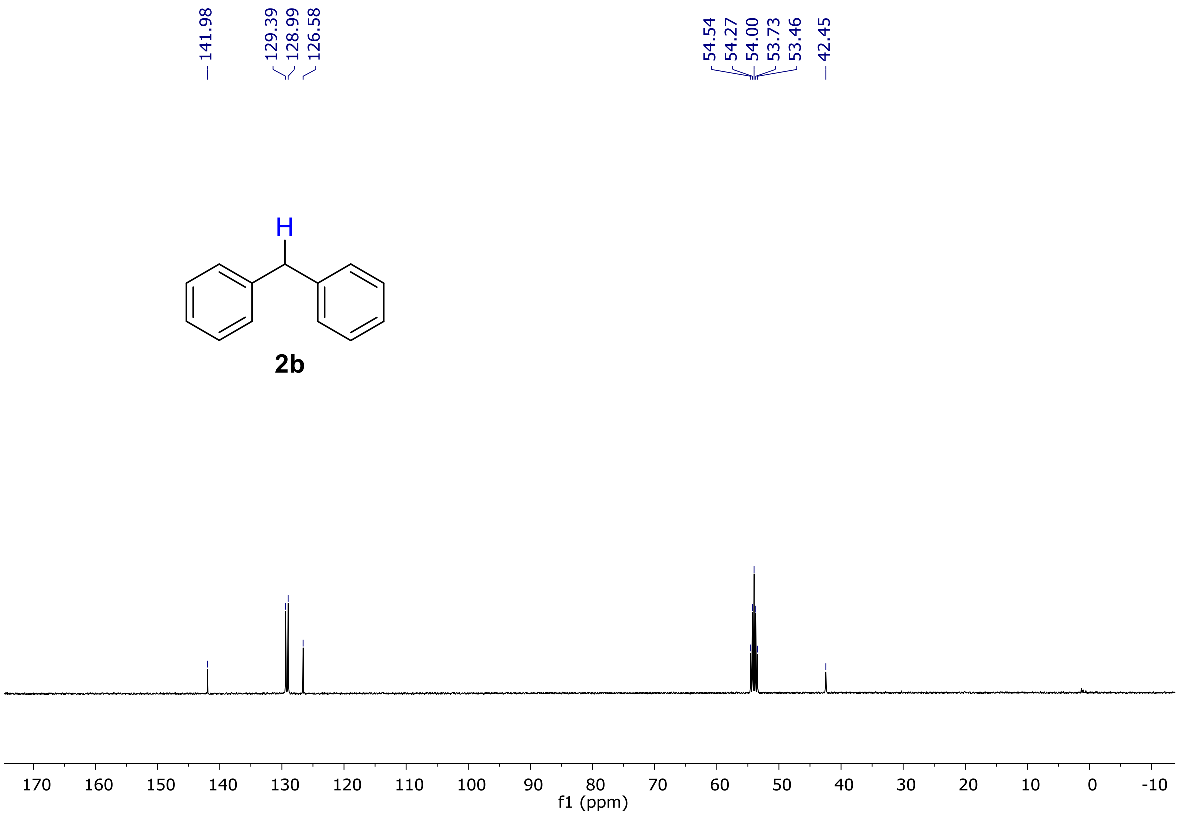

*Figure S41. $^{13}C\{^{1}H\}$ NMR spectrum of the isolated product **2b**.*

9.1.3 ¹H and ¹³C NMR spectra of the isolated hexaethyldisiloxane

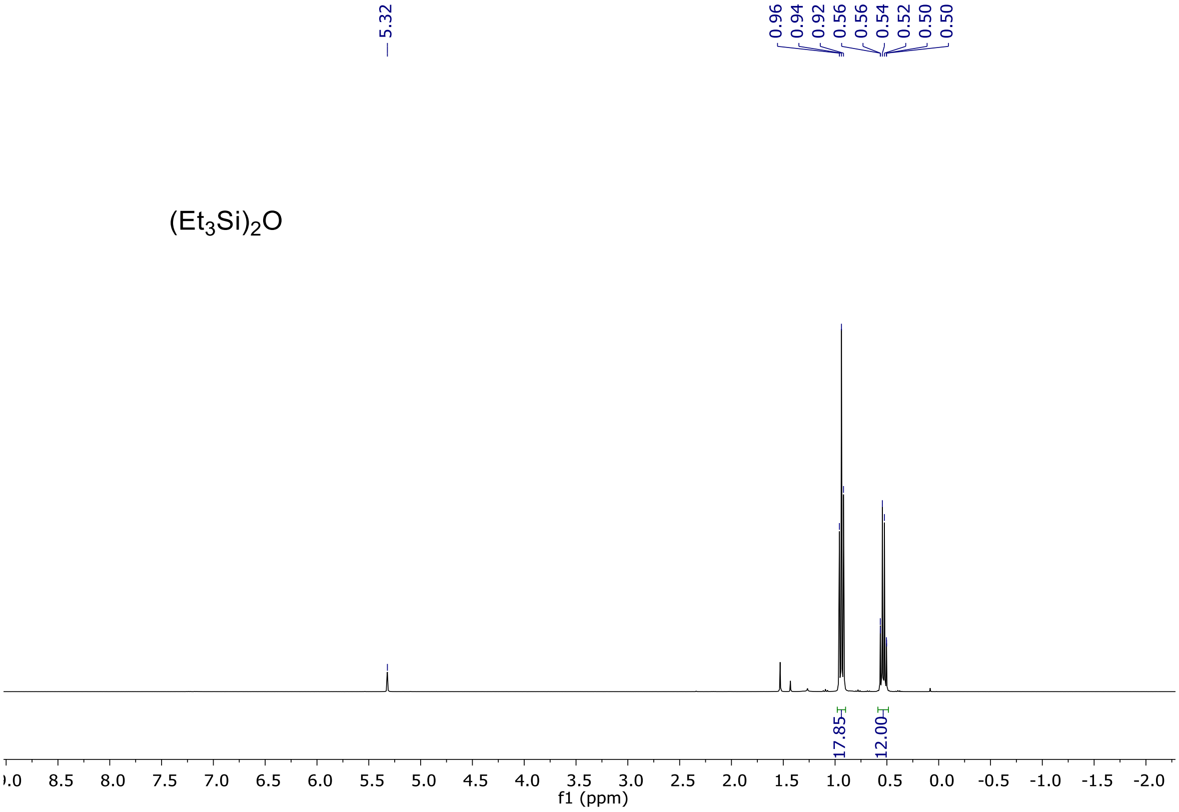

Figure S42. ¹H NMR spectrum of the isolated ***$(Et_3Si)_2O$****.*

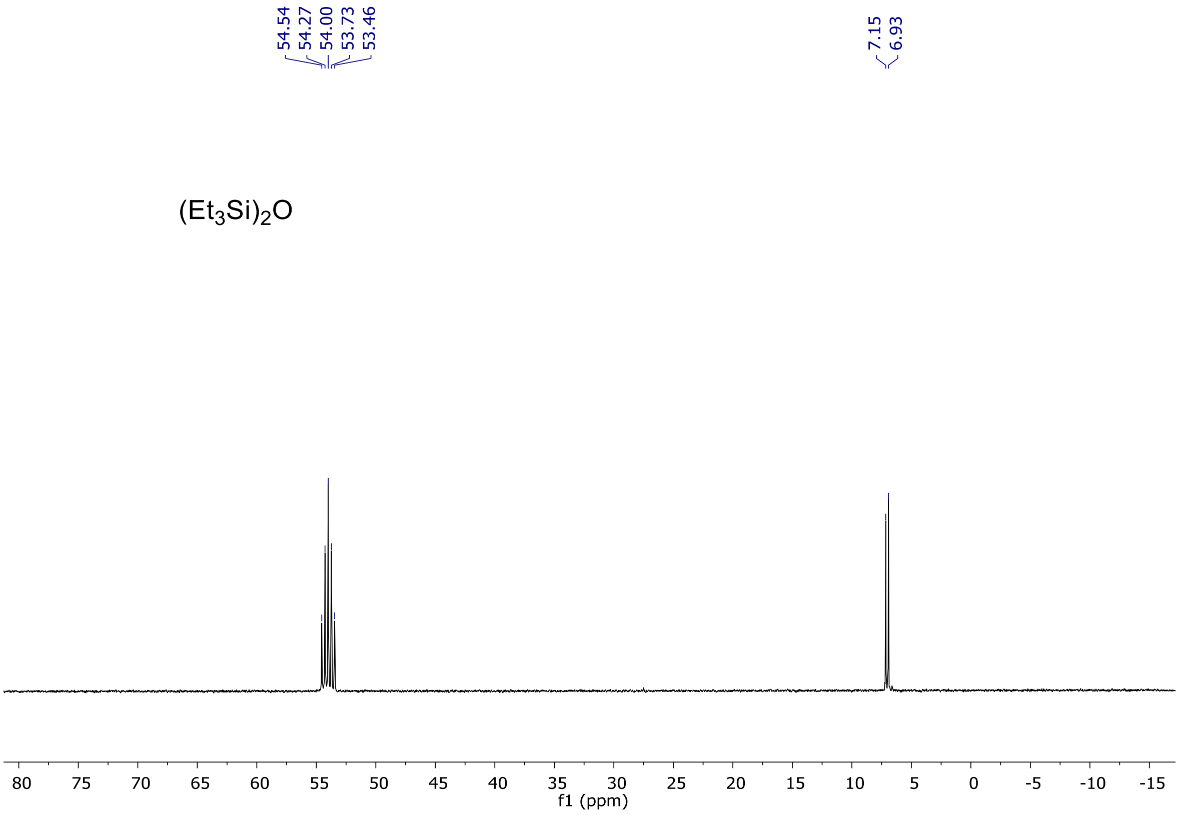

Figure S43. ¹³C{¹H} NMR spectrum of the isolated ***$(Et_3Si)_2O$****.*

9.1.4 ^{1}H and ^{13}C NMR spectra of the isolated (oxybis(ethane-1,1-diyl))dibenzene 3a

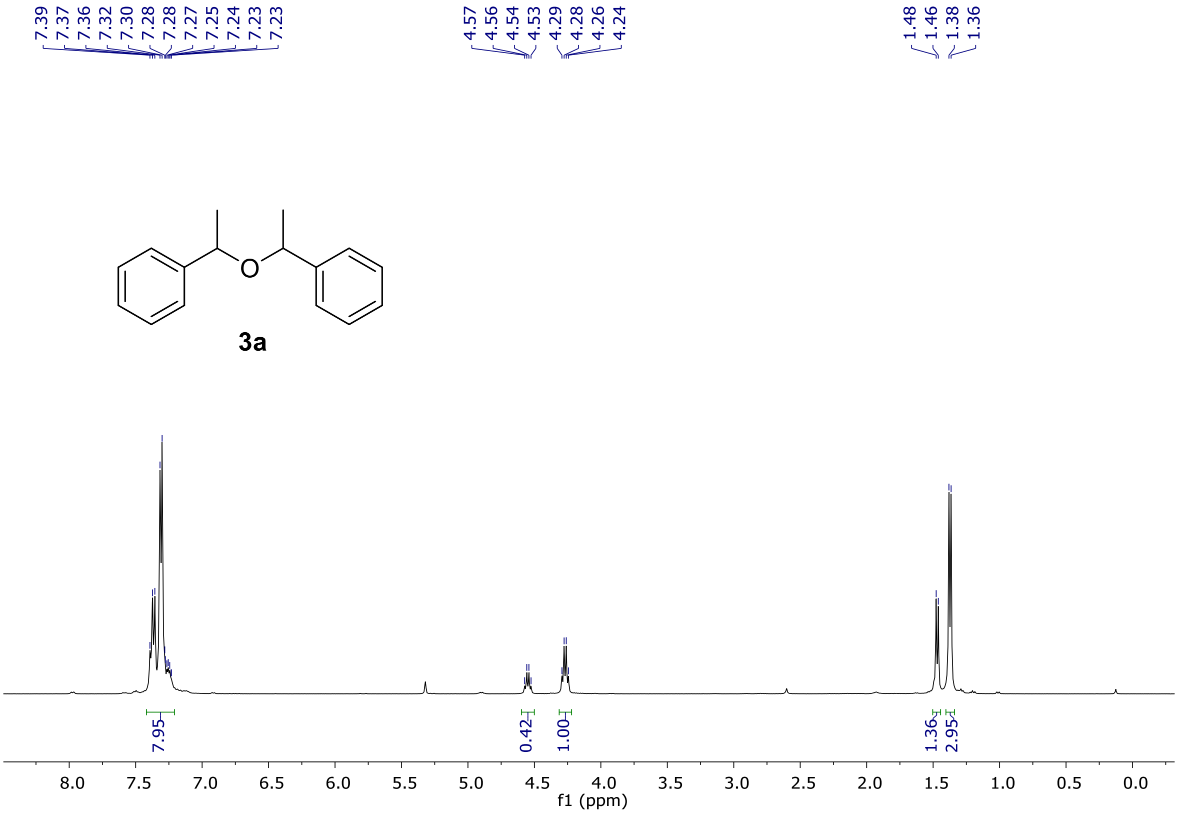

*Figure S44. ^{1}H NMR spectrum of the isolated ether **3a**.*

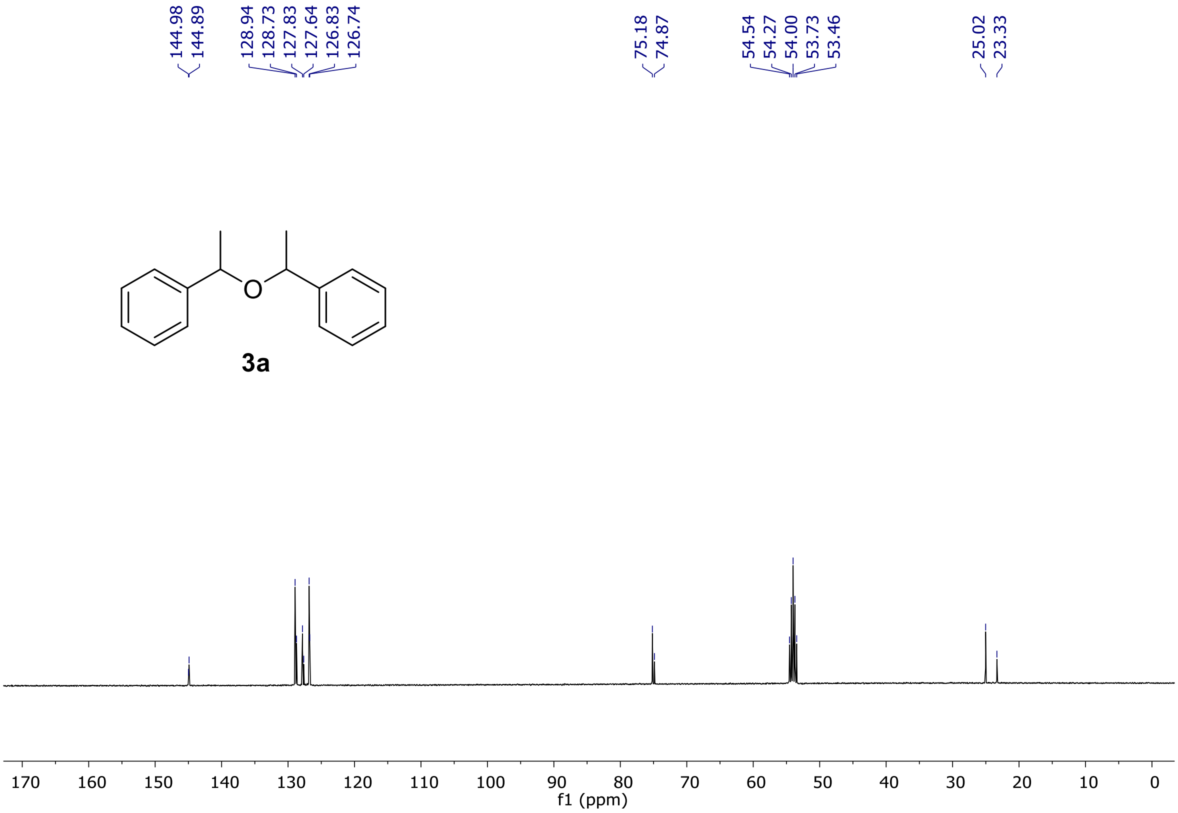

*Figure S45. $^{13}C\{^{1}H\}$ NMR spectrum of the isolated ether **3a**.*

9.2 ^{1}H and ^{13}C NMR Spectra of alkanes obtained by Ketones deoxygenation

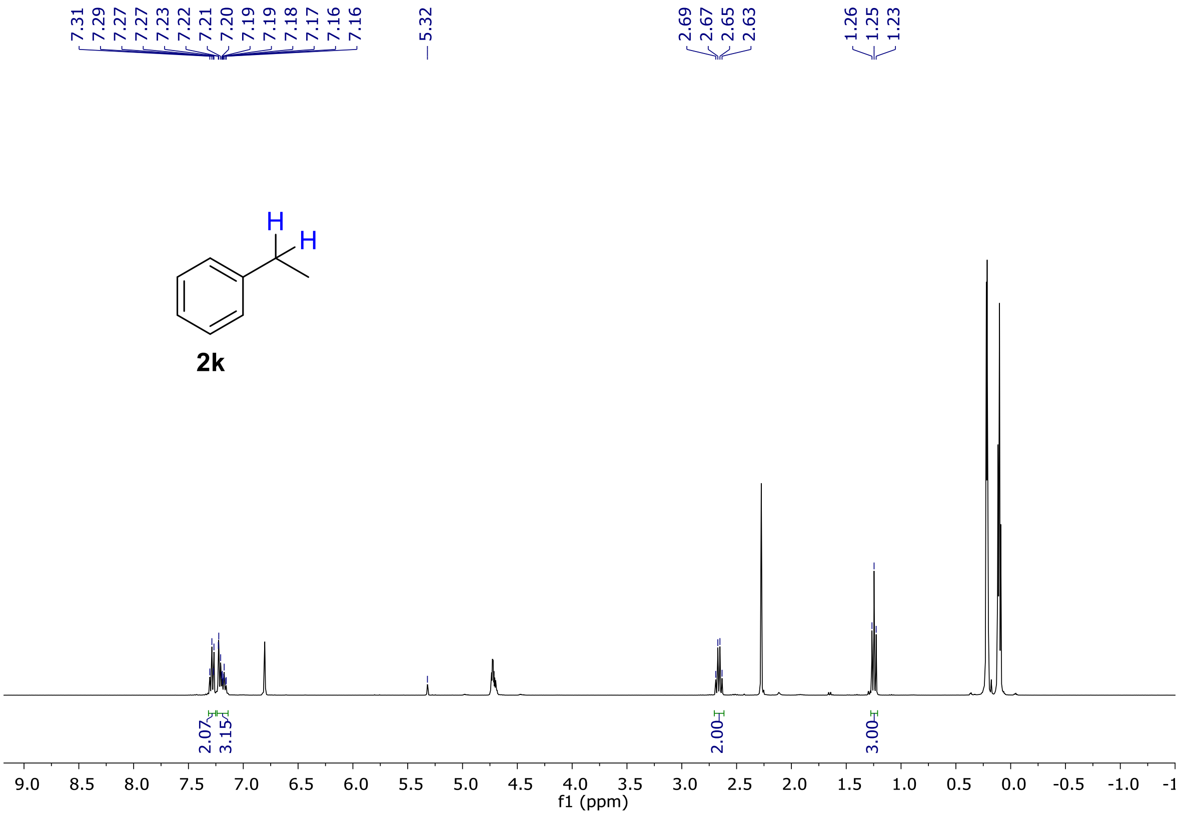

*Figure S46. ^{1}H NMR spectrum of the crude reaction mixture of **2k**.*

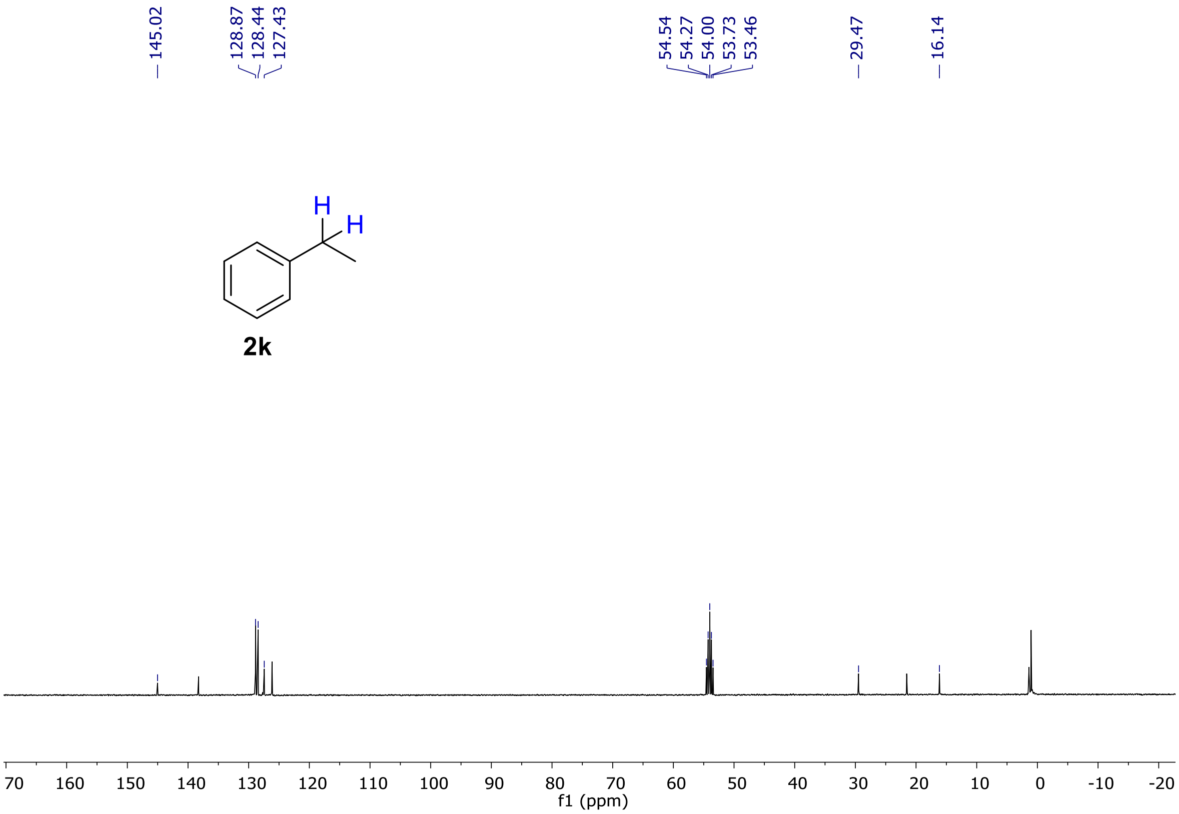

*Figure S47. ^{13}C{^{1}H} NMR spectrum of the crude reaction mixture of **2k**.*

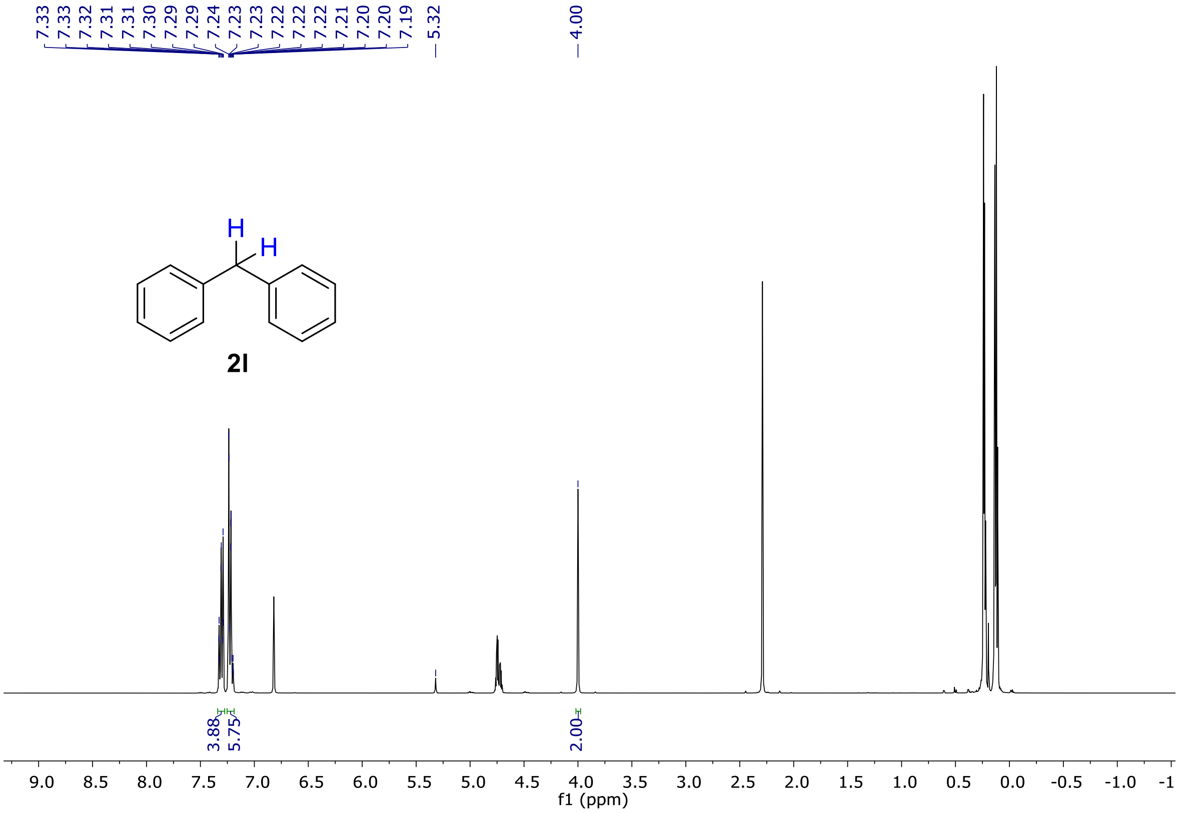

*Figure S48. ^{1}H NMR spectrum of the crude reaction mixture of **2l**.*

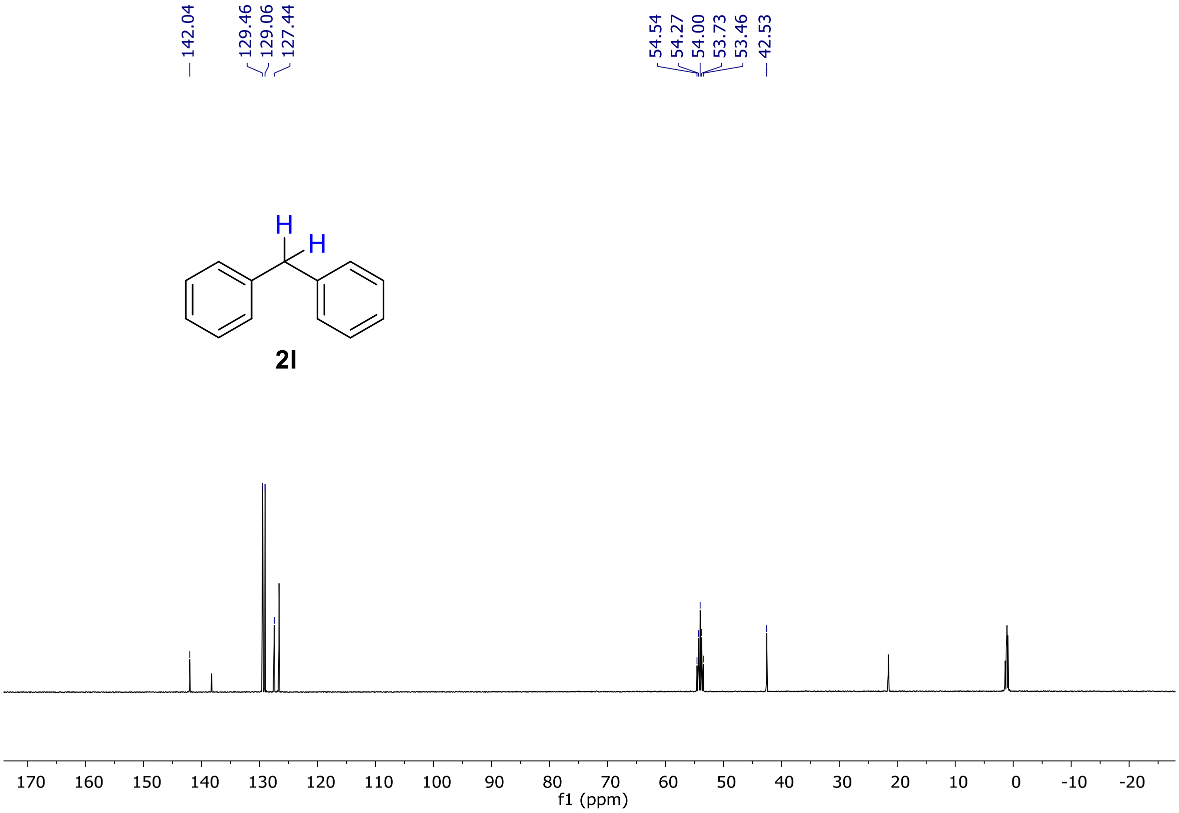

*Figure S49. $^{13}C\{^{1}H\}$ NMR spectrum of the crude reaction mixture of **2l**.*

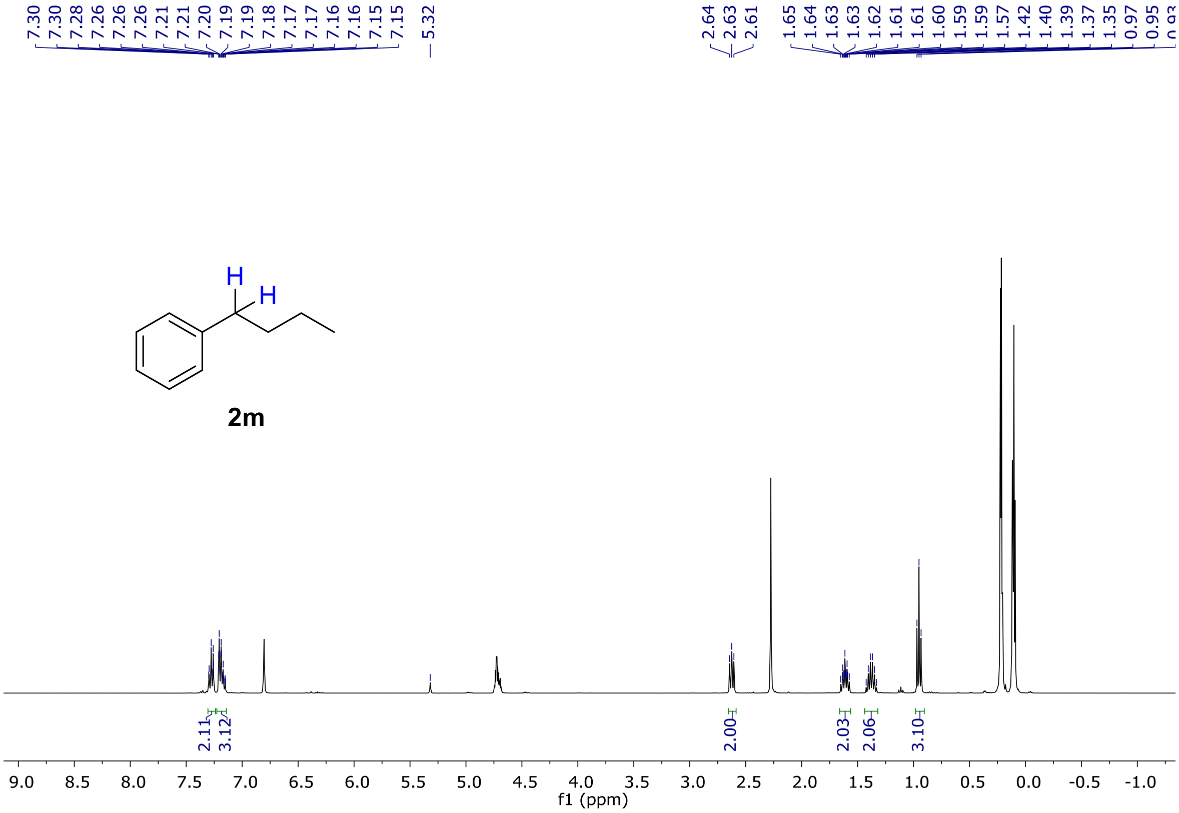

*Figure S50. ^{1}H NMR spectrum of the crude reaction mixture of **2m**.*

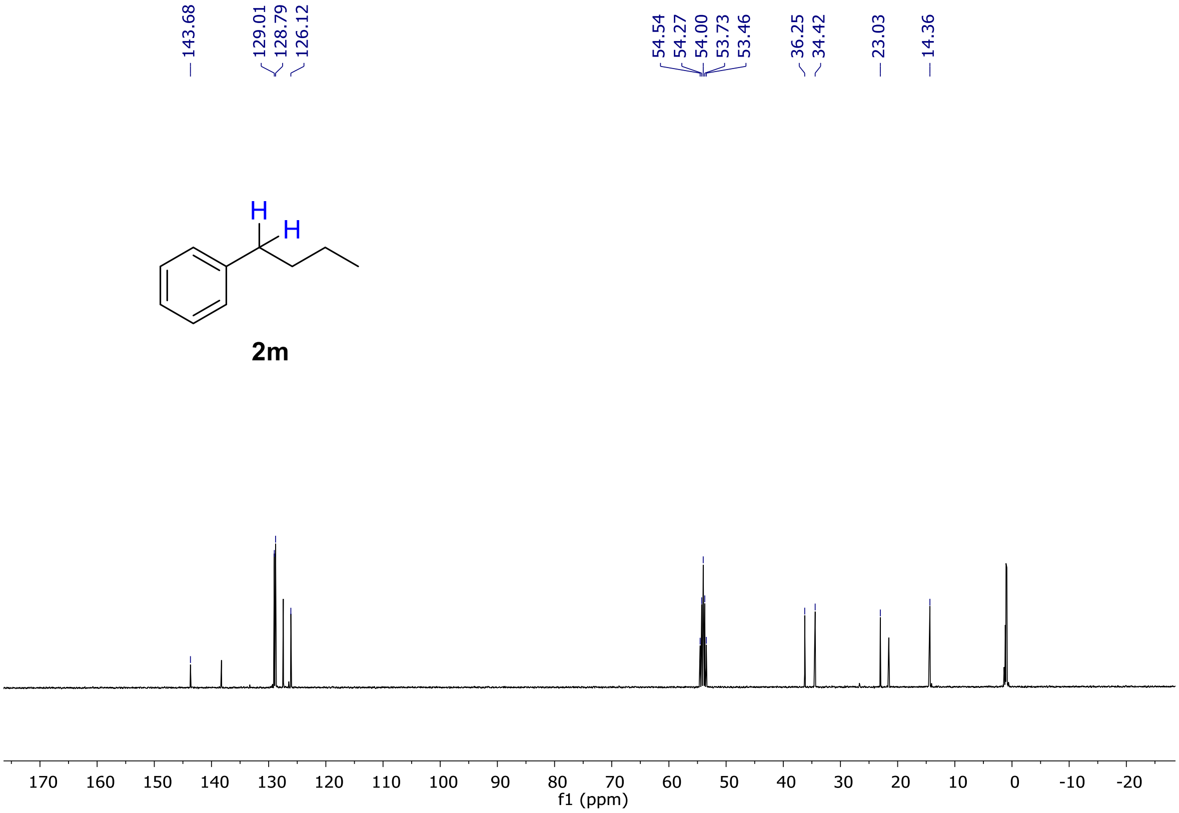

*Figure S51. ^{13}C{^{1}H} NMR spectrum of the crude reaction mixture of **2m**.*

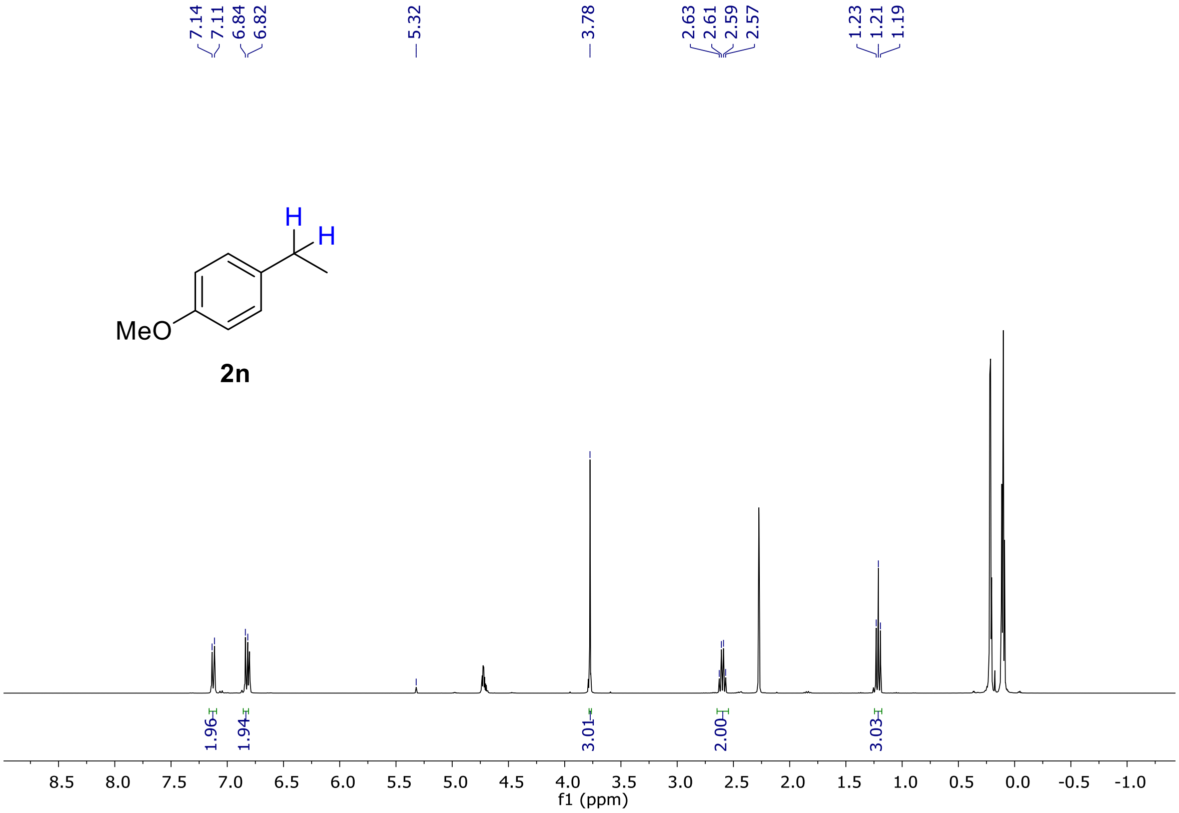

*Figure S52. ^{1}H NMR spectrum of the crude reaction mixture of **2n**.*

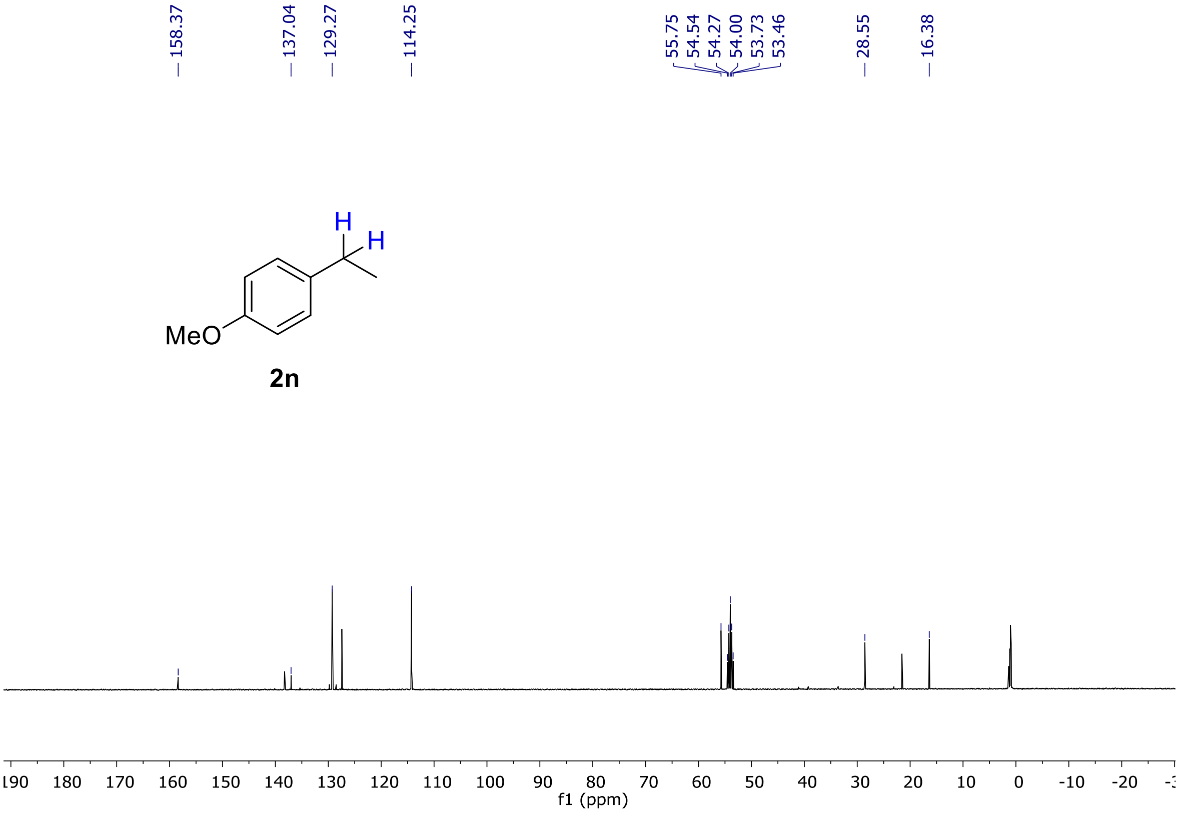

*Figure S53. $^{13}C\{^{1}H\}$ NMR spectrum of the crude reaction mixture of **2n**.*

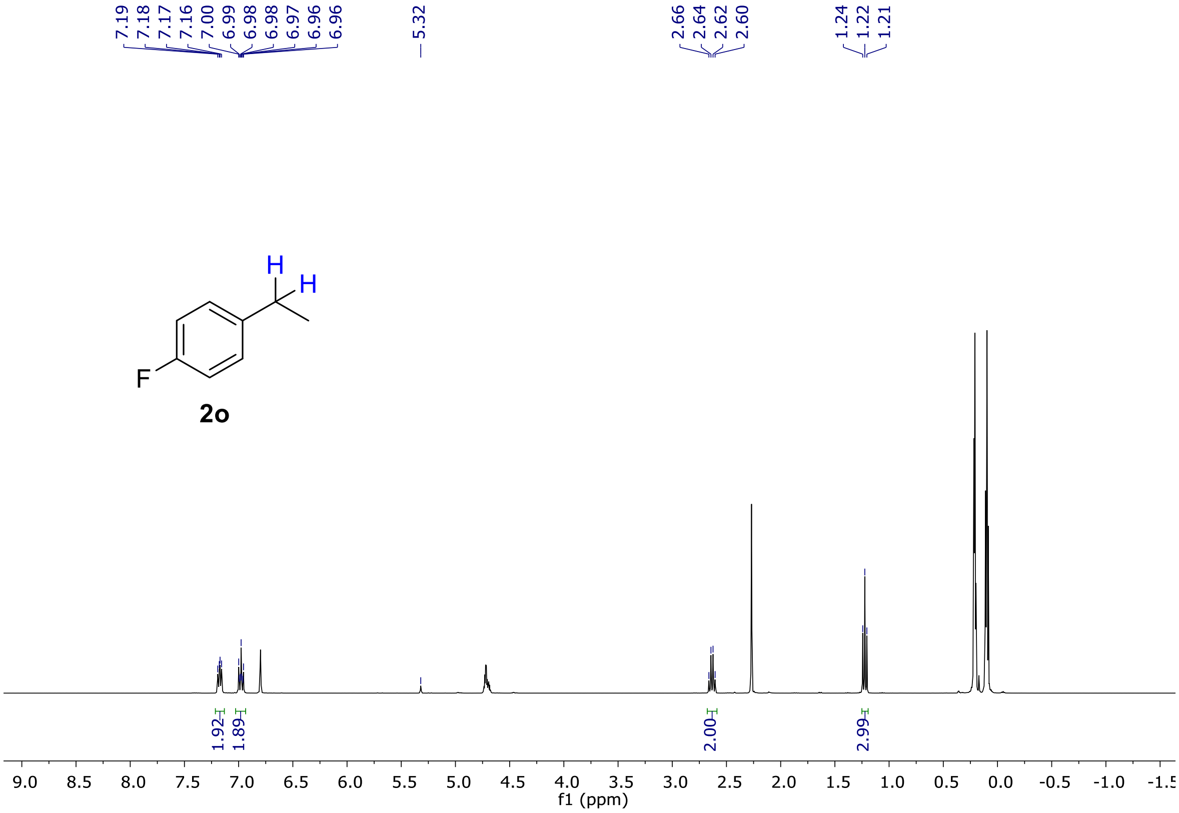

Figure S54. ^{1}H NMR spectrum of the crude reaction mixture of ***2o****.*

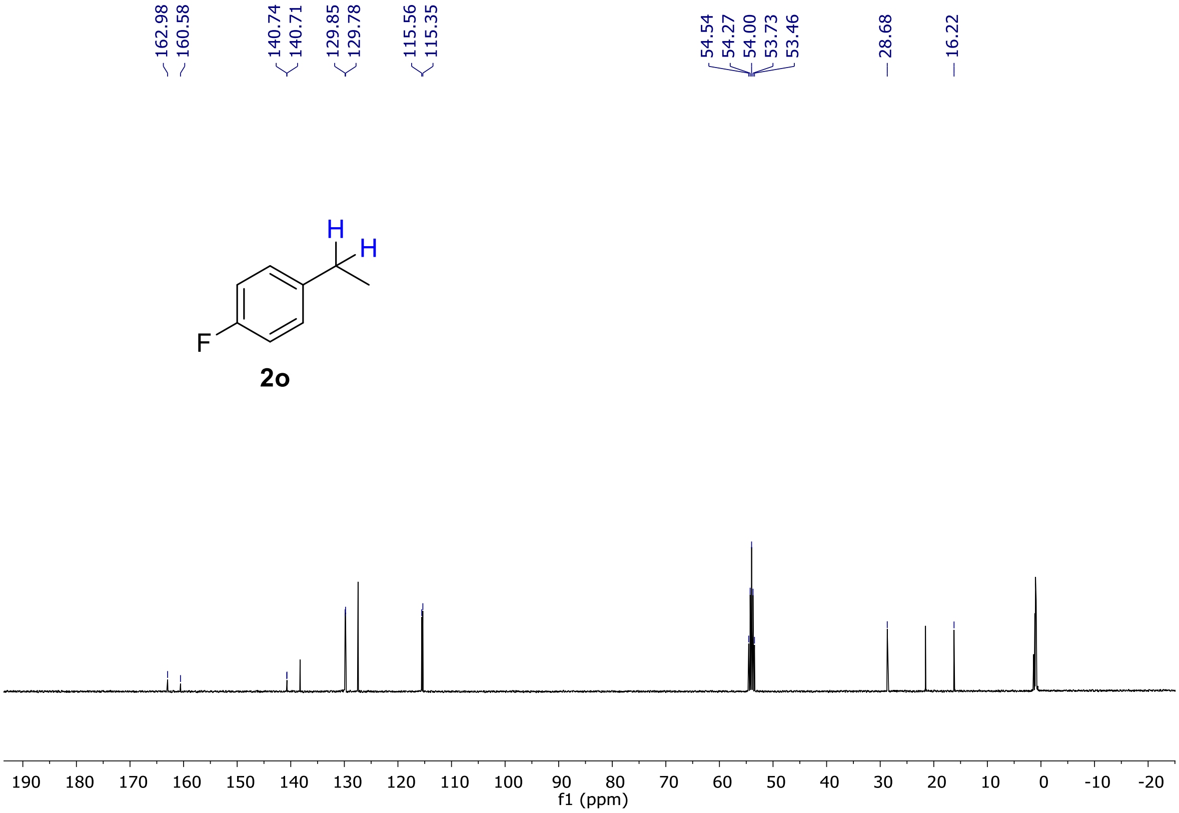

Figure S55. $^{13}C\{^{1}H\}$ NMR spectrum of the crude reaction mixture of ***2o****.*

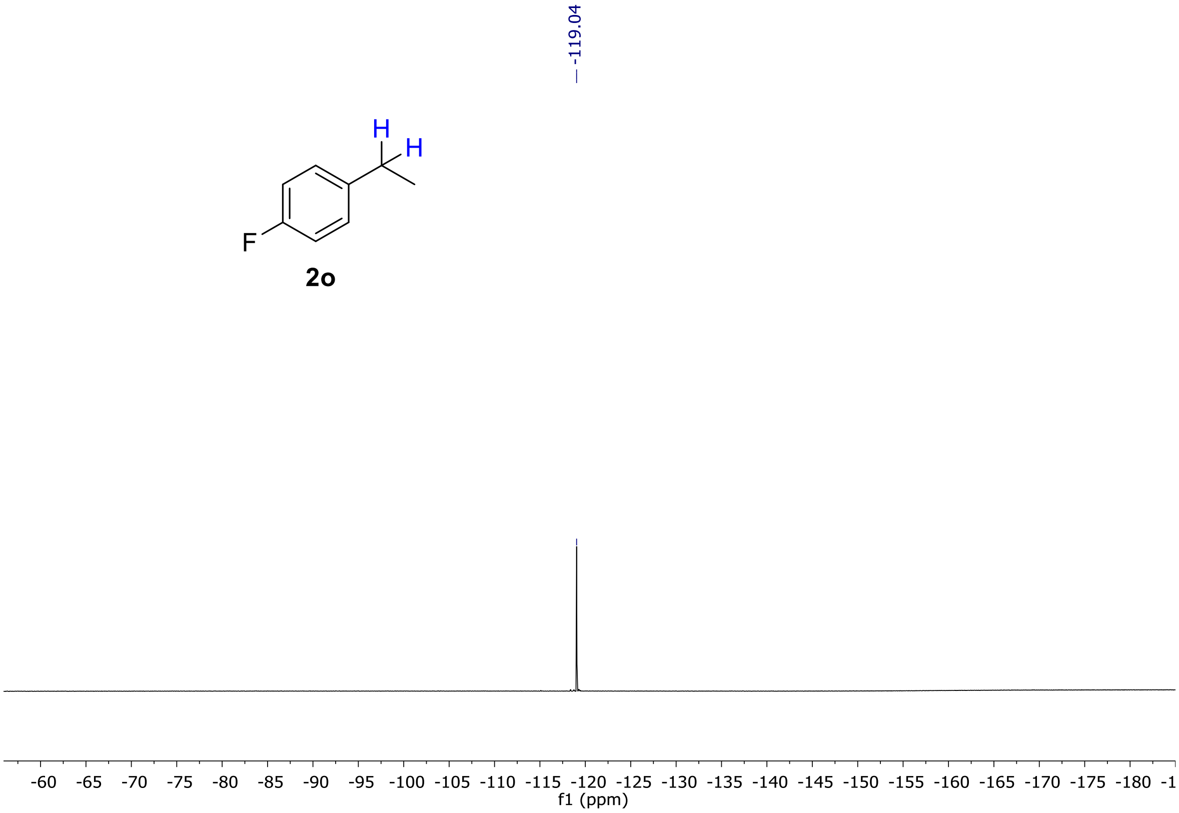

Figure S56. ^{19}*F NMR spectrum of the crude reaction mixture of* ***2o****.*

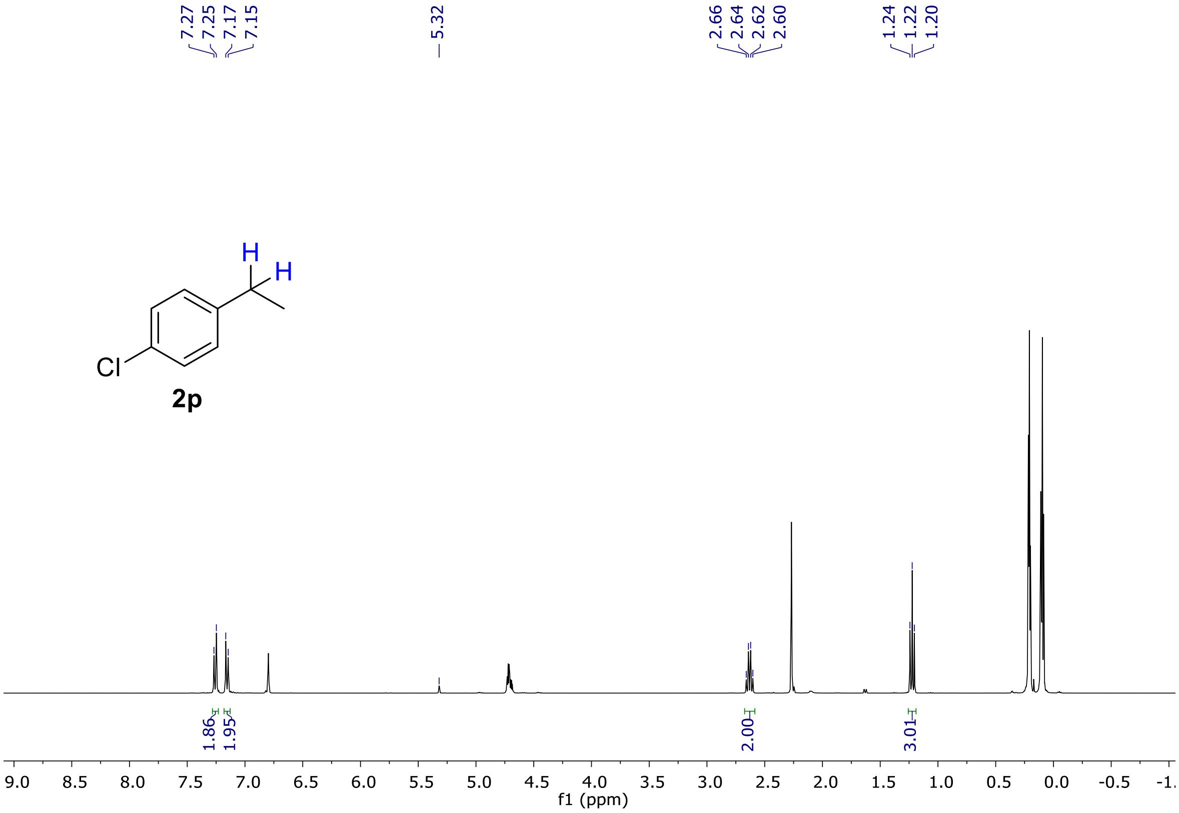

*Figure S57. ^{1}H NMR spectrum of the crude reaction mixture of **2p**.*

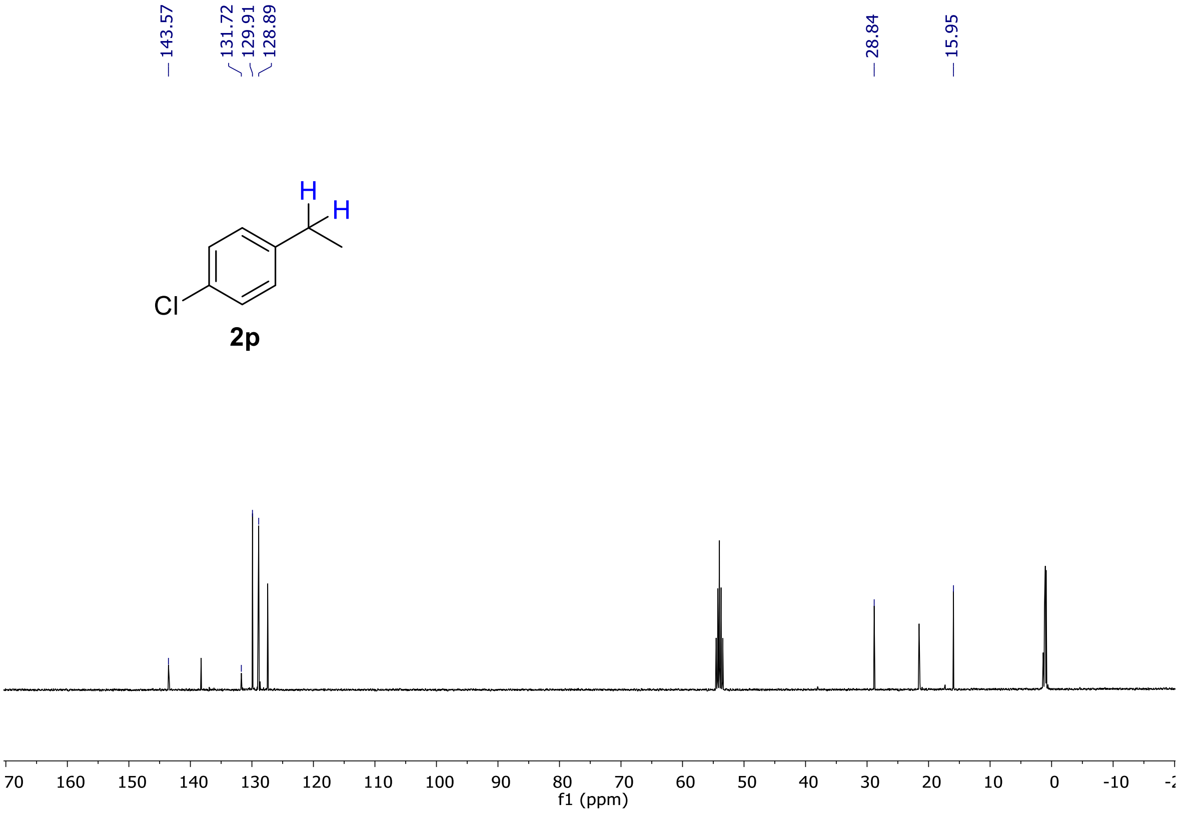

*Figure S58. ^{13}C{^{1}H} NMR spectrum of the crude reaction mixture of **2p**.*

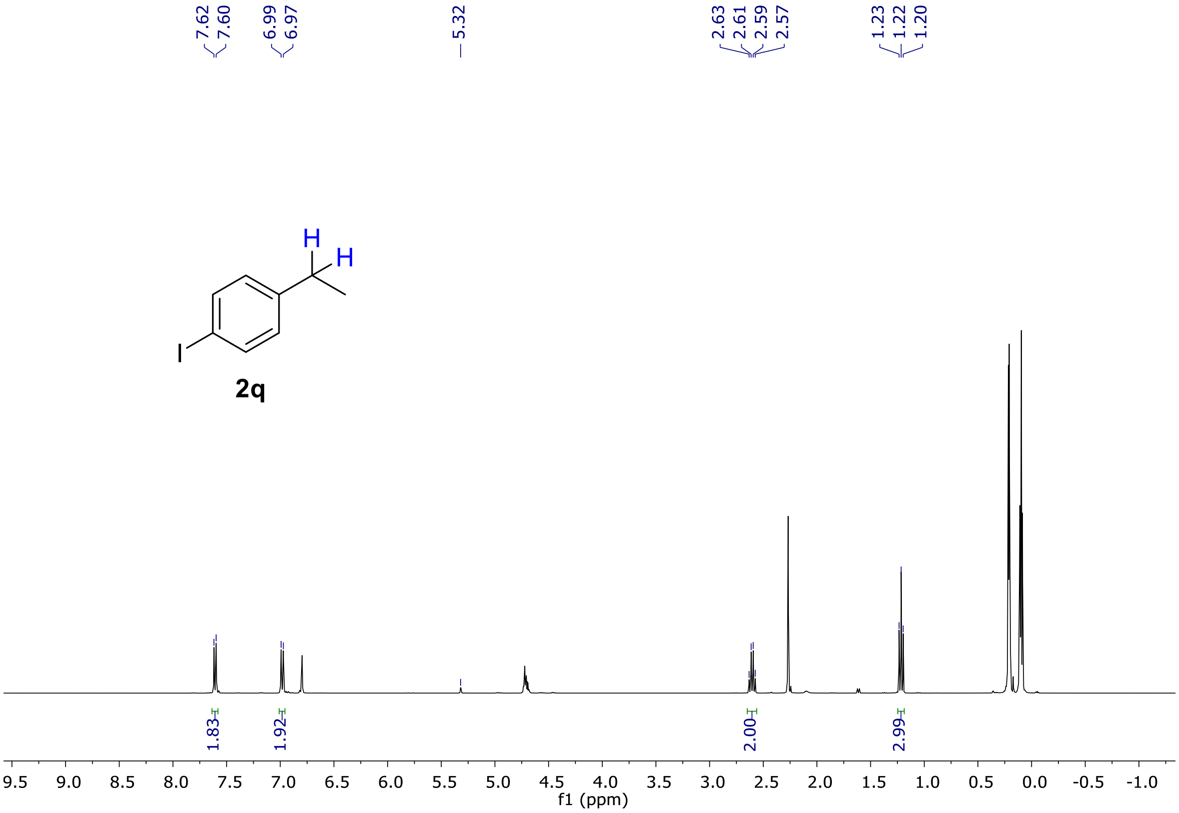

Figure S59. ¹H NMR spectrum of the crude reaction mixture of ***2q****.*

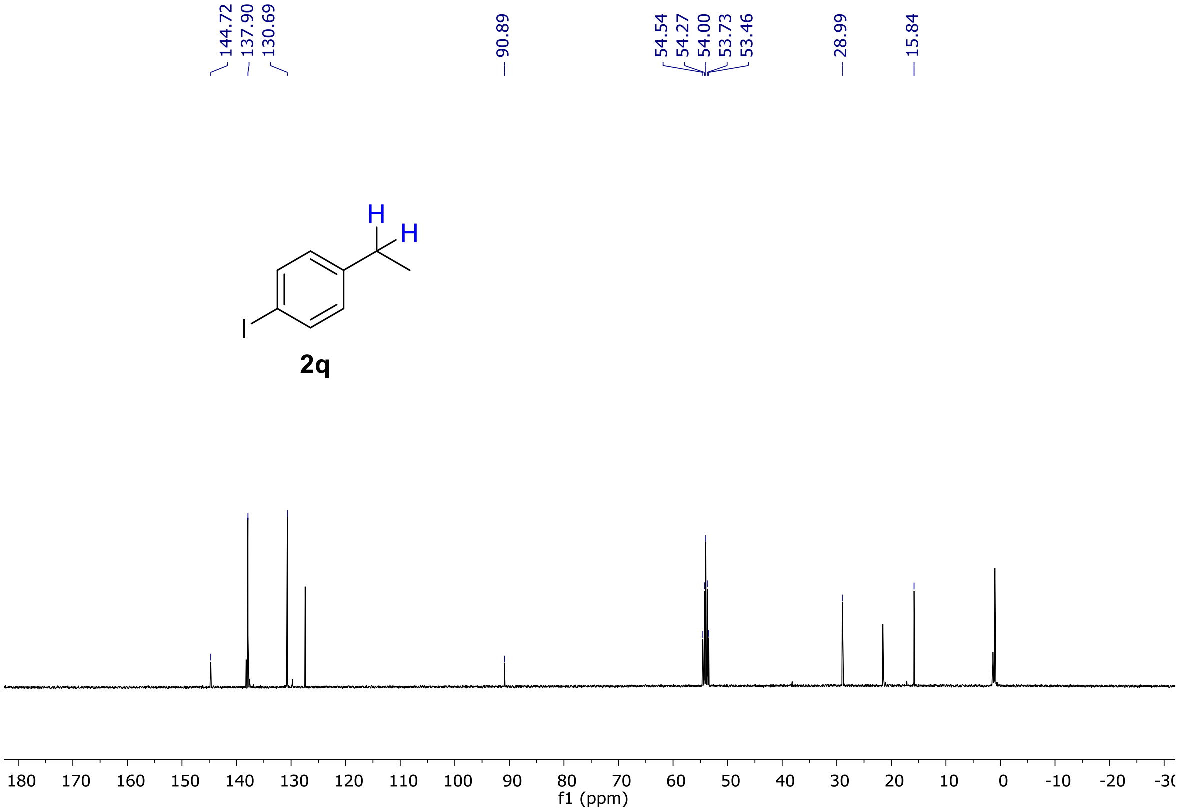

Figure S60. $^{13}C\{^1H\}$ NMR spectrum of the crude reaction mixture of ***2q****.*

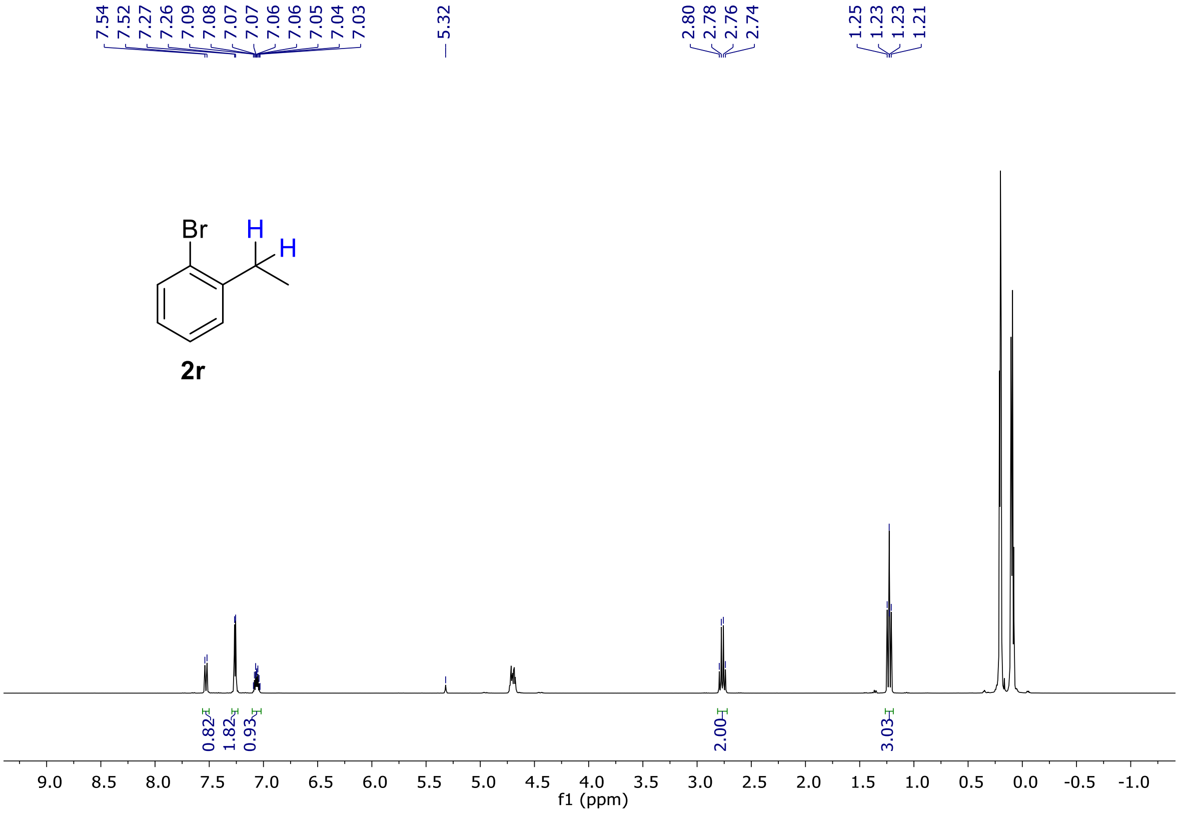

*Figure S61. ^{1}H NMR spectrum of the crude reaction mixture of **2r**.*

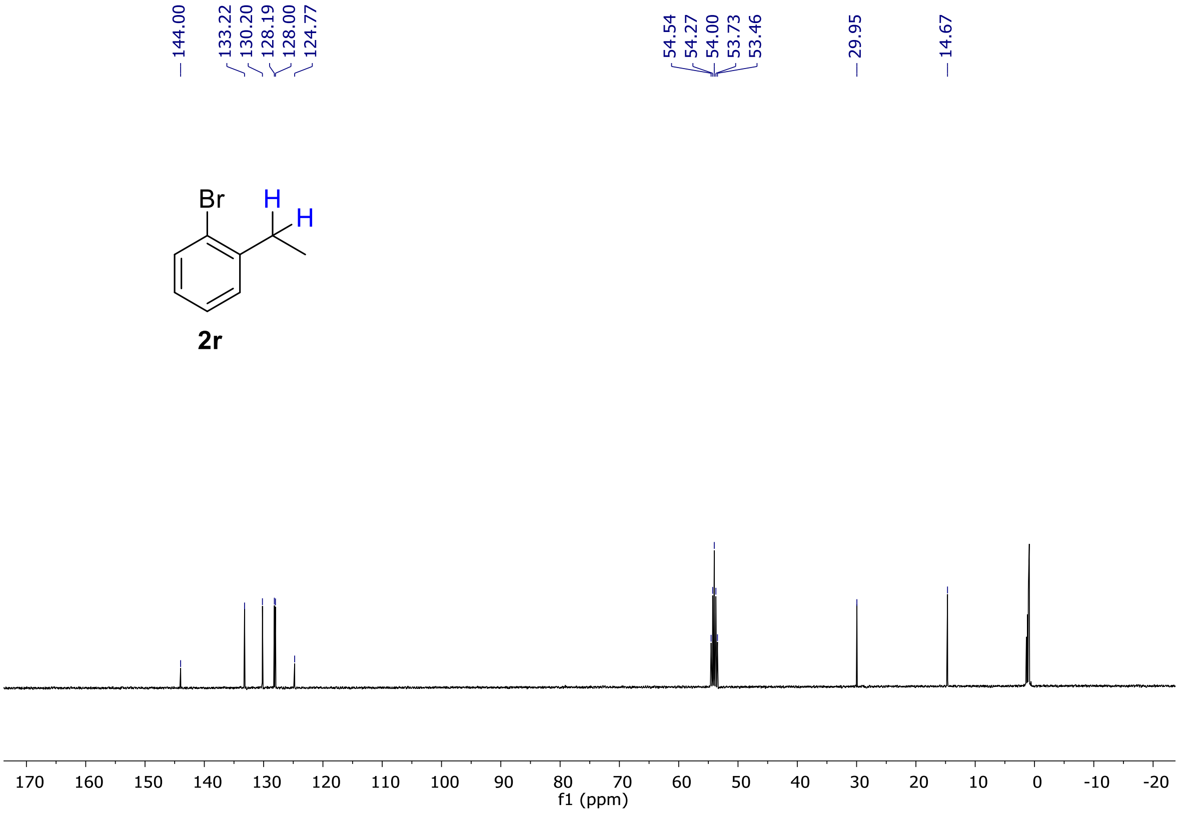

*Figure S62. ^{13}C{^{1}H} NMR spectrum of the crude reaction mixture of **2r**.*

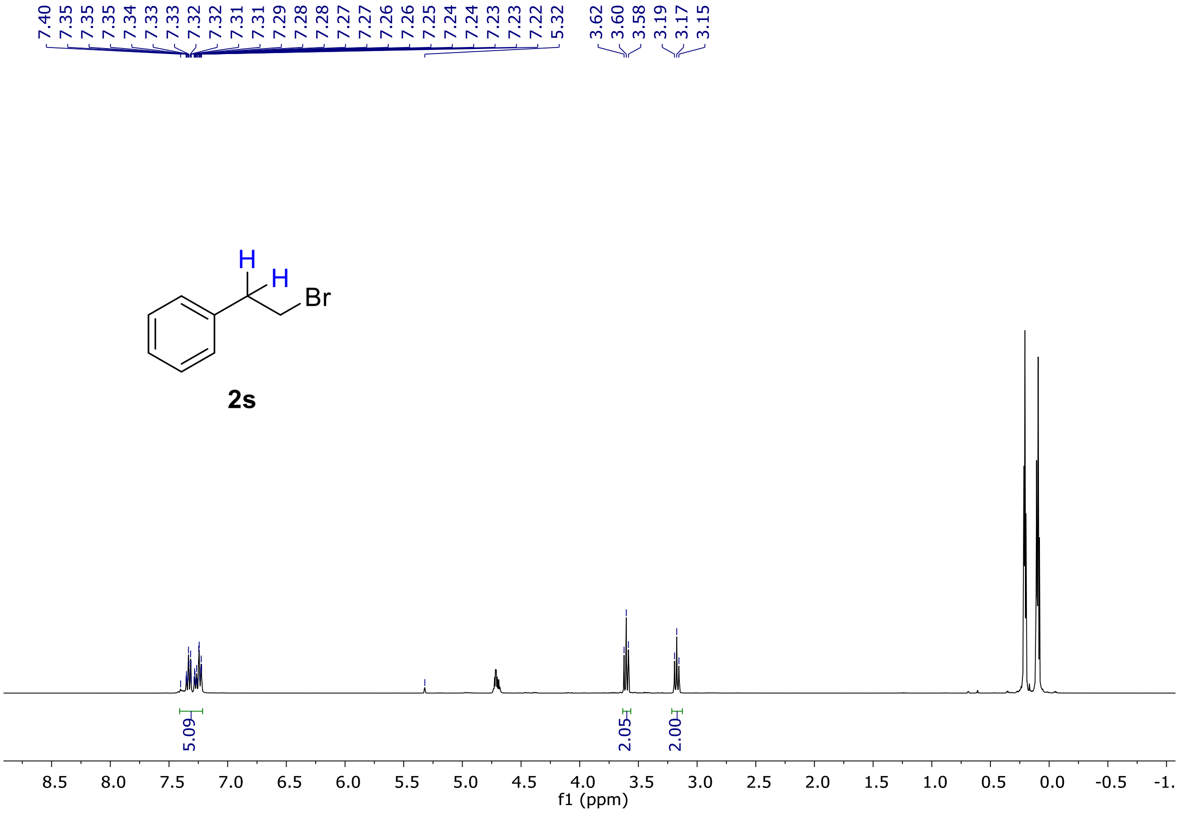

*Figure S63. ^{1}H NMR spectrum of the crude reaction mixture of **2s**.*

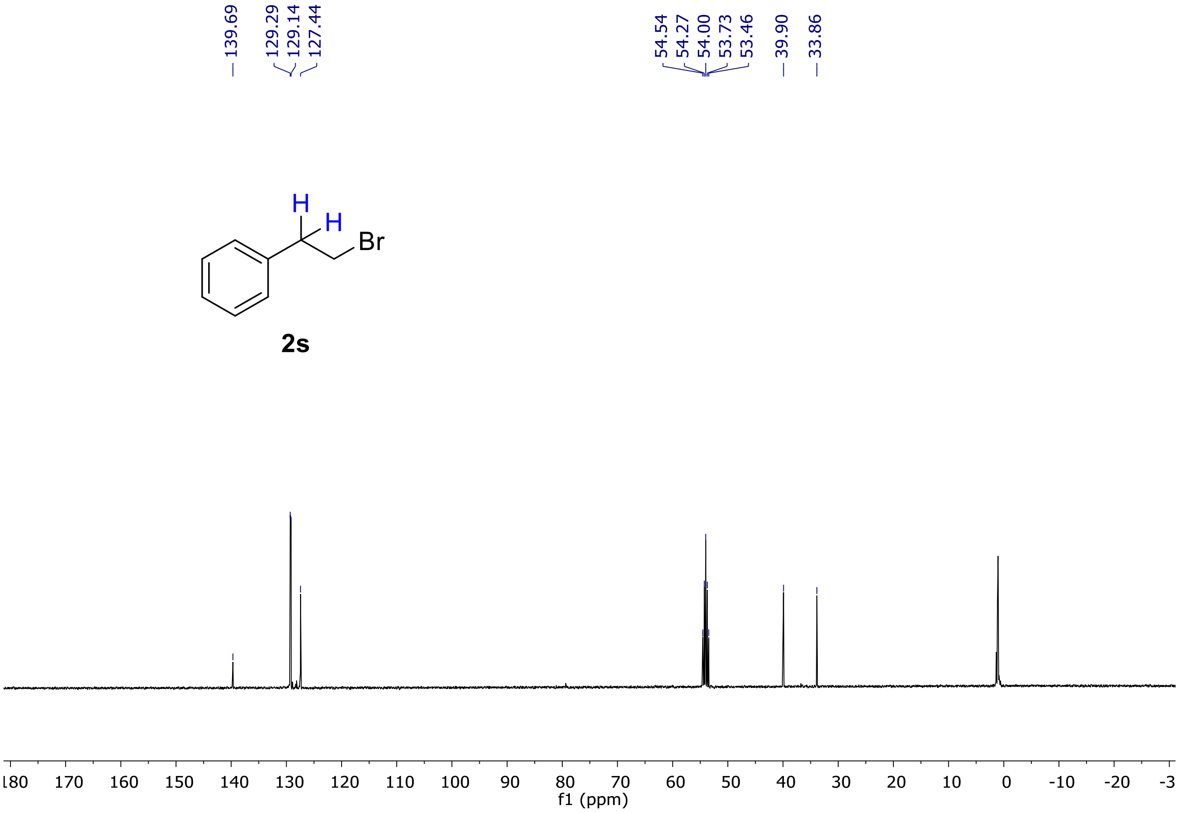

*Figure S64. ^{13}C{^{1}H} NMR spectrum of the crude reaction mixture of **2s**.*

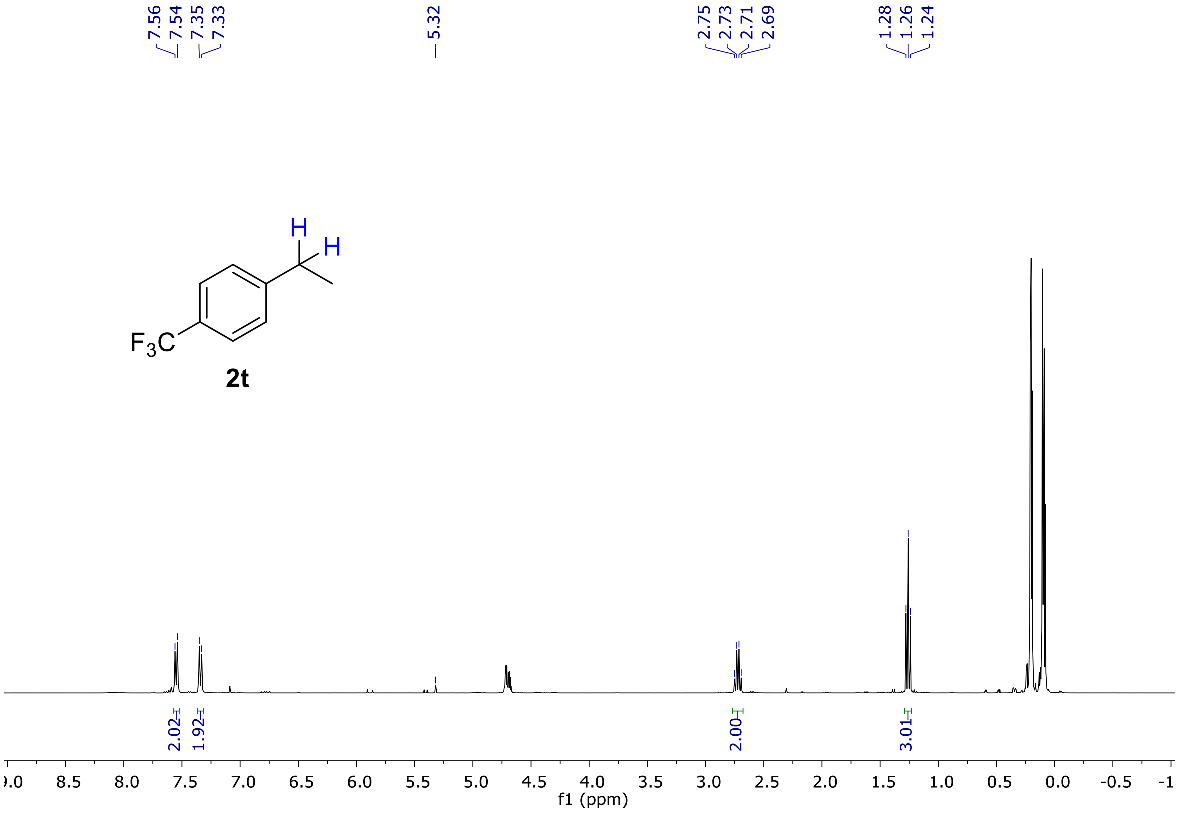

Figure S65. [1]*H NMR spectrum of the crude reaction mixture of* ***2t*****.**

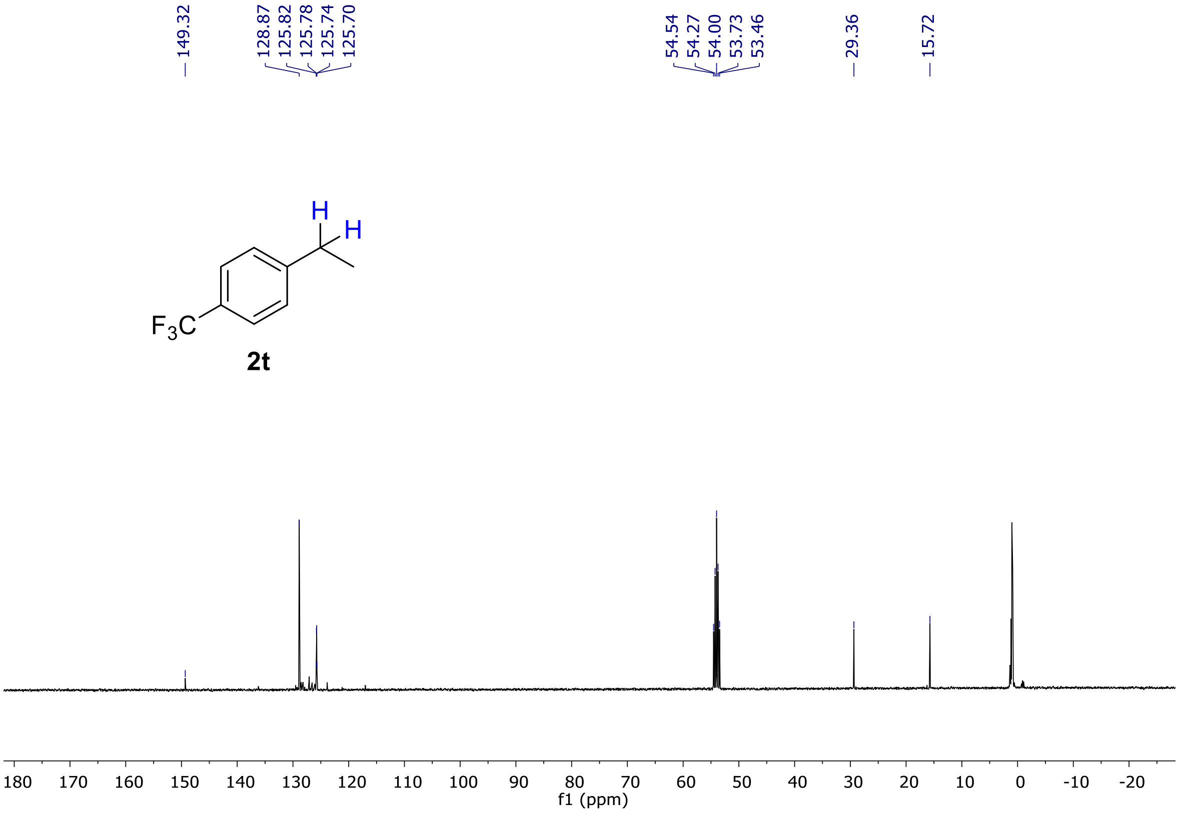

Figure S66. [13]*C{*[1]*H} NMR spectrum of the crude reaction mixture of* ***2t*****.**

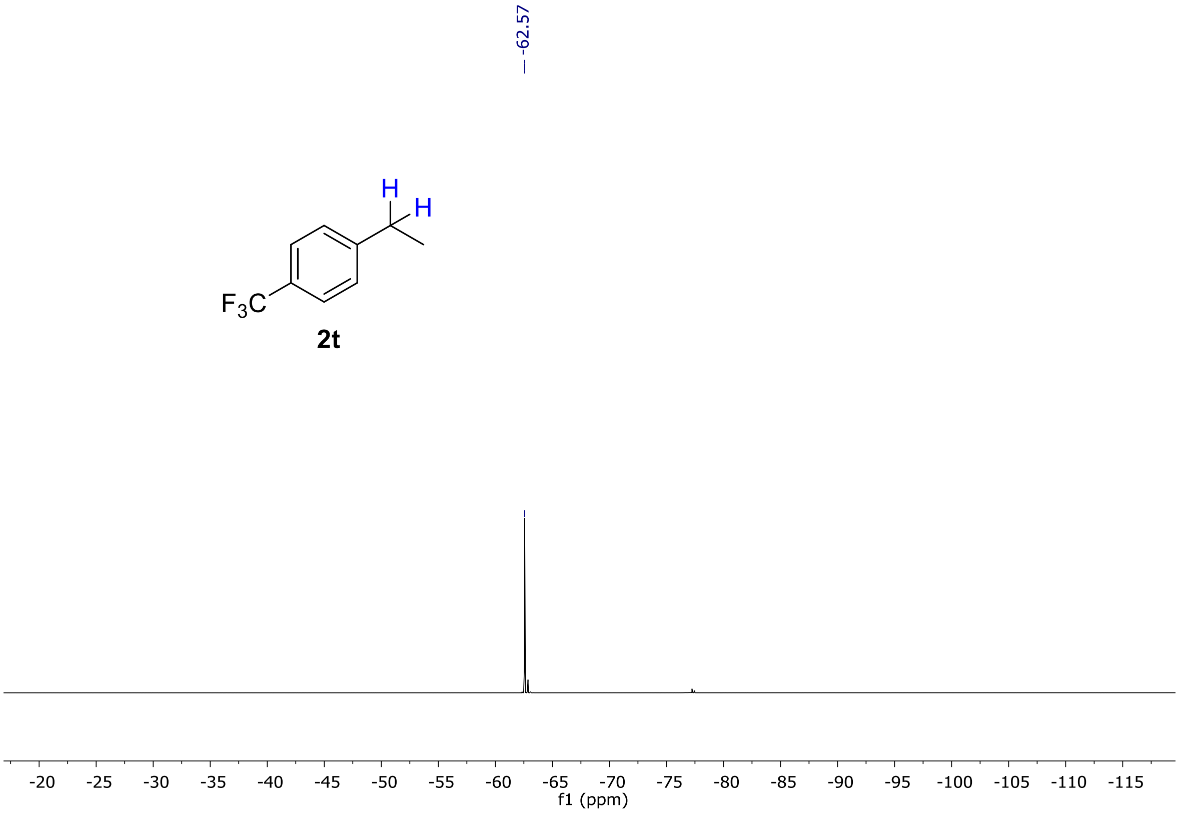

Figure S67. $^{19}F\{^{1}H\}$ *NMR spectrum of the crude reaction mixture of* ***2t***.

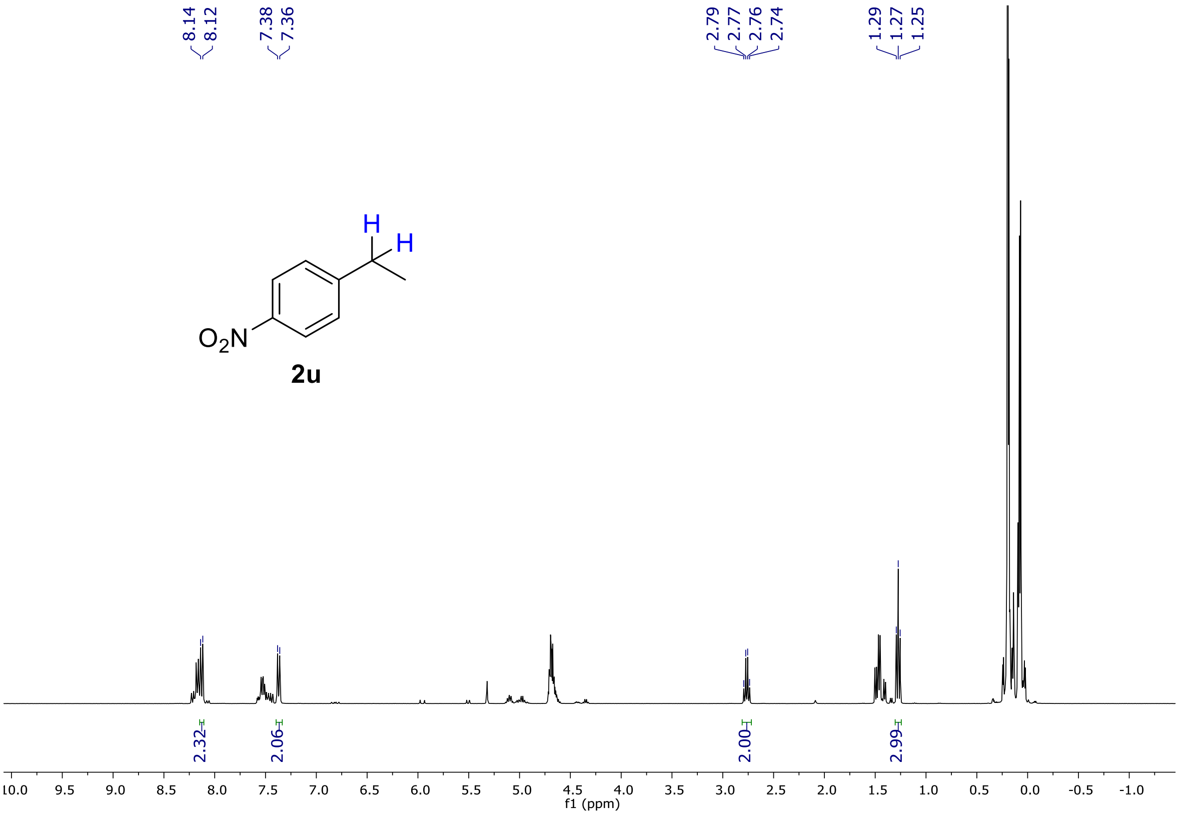

Figure S68. ^{1}H *NMR spectrum of the crude reaction mixture of* ***2u***.

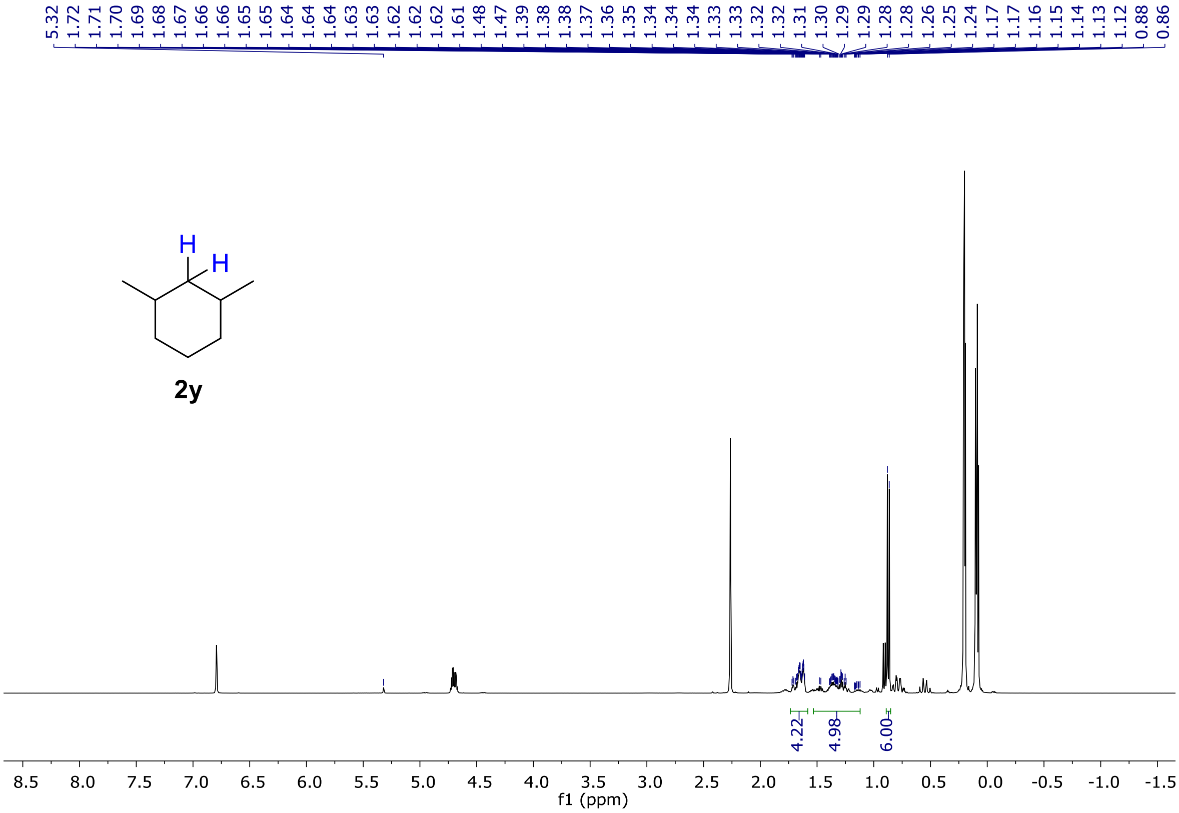

*Figure S69. ^{1}H NMR spectrum of the crude reaction mixture of **2y**.*

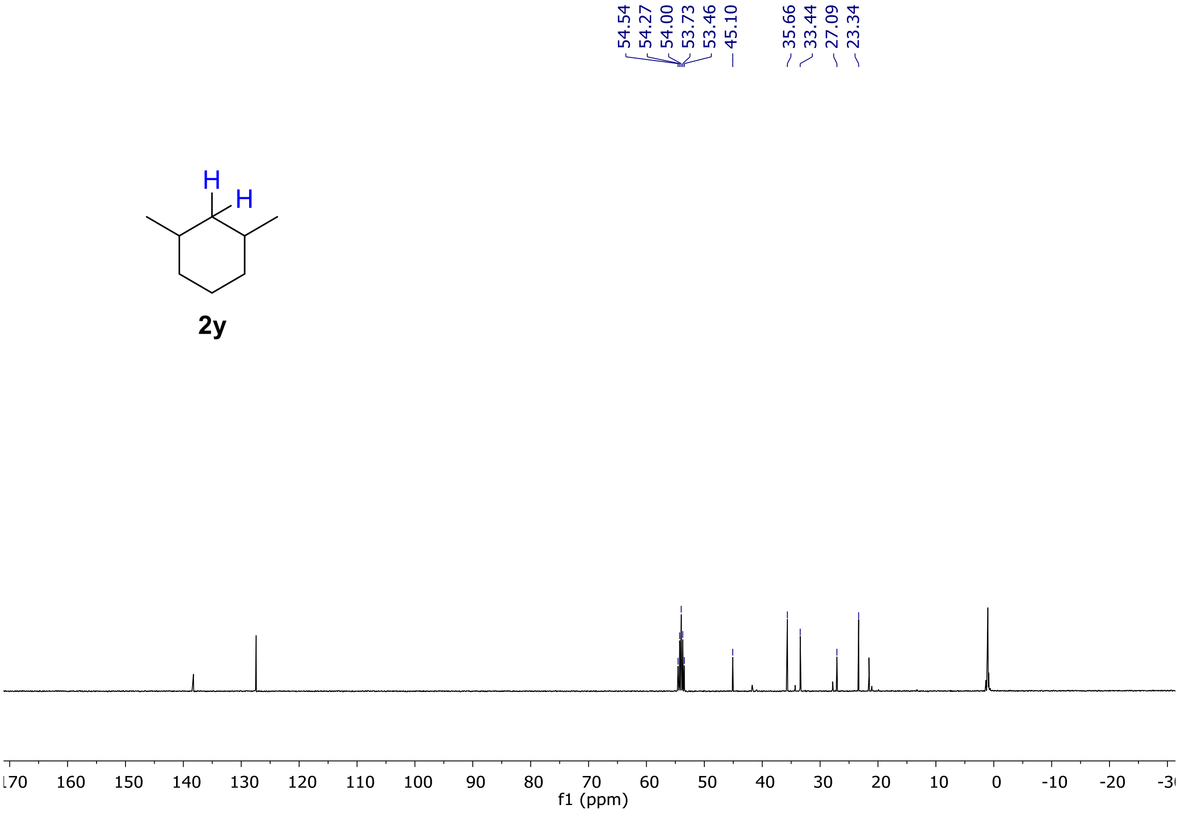

*Figure S70. $^{13}C\{^{1}H\}$ NMR spectrum of the crude reaction mixture of **2y**.*

9.3 ^{1}H and ^{13}C NMR Spectra of alkanes obtained by Hydrodehalogenation of alkyl halides using TMDS

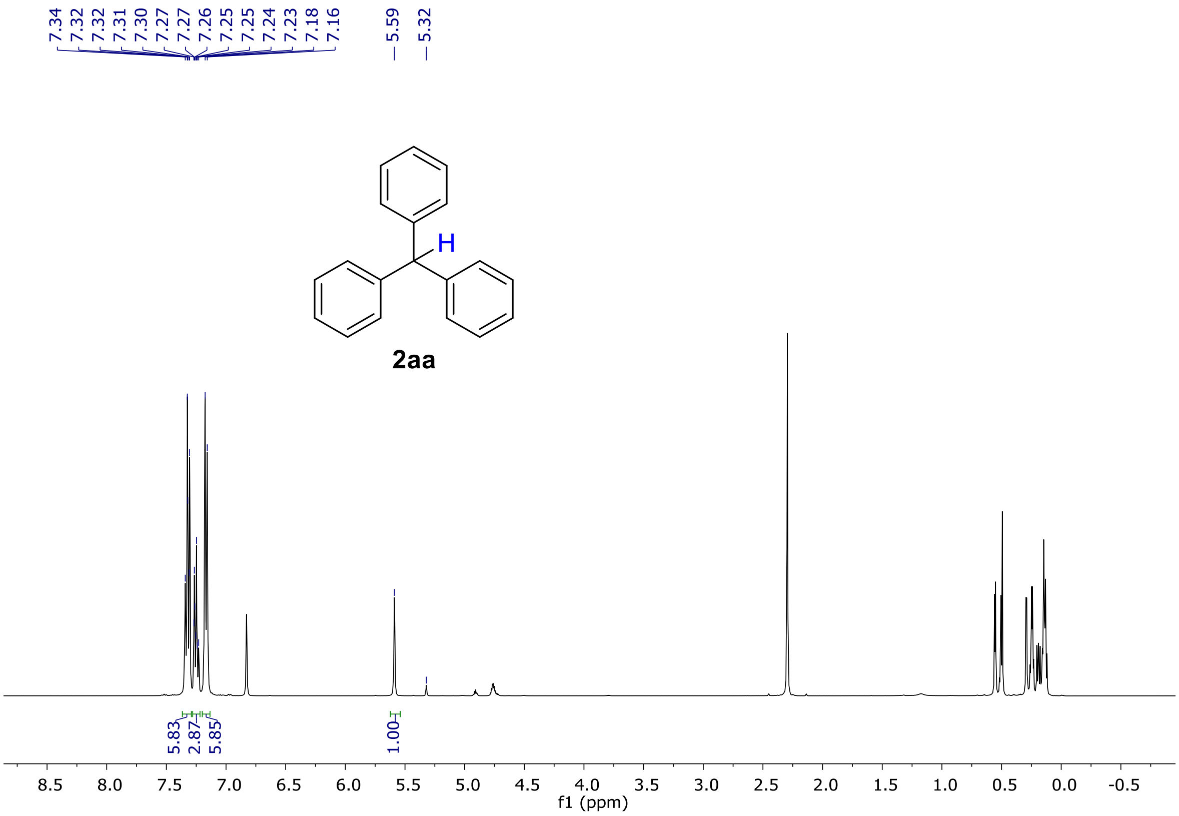

*Figure S71. ^{1}H NMR spectrum of the crude reaction mixture of **2aa**.*

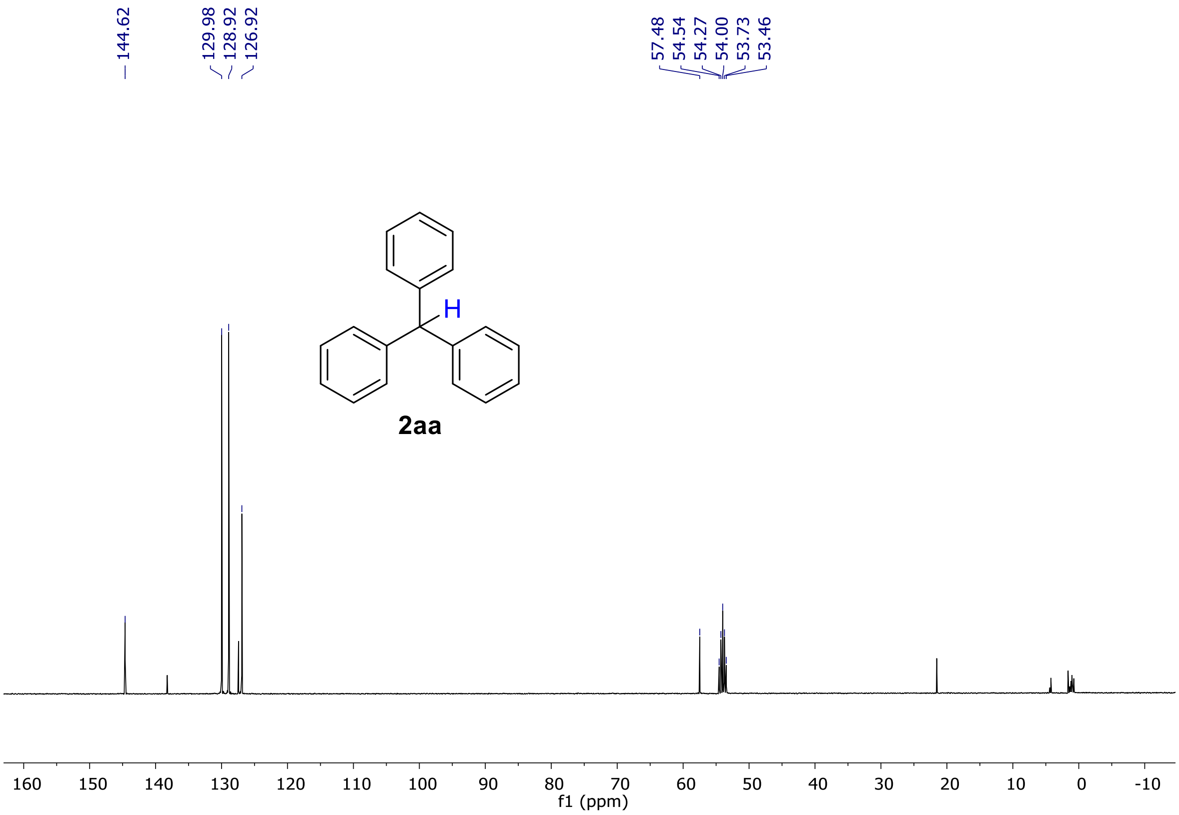

*Figure S72. $^{13}C\{^{1}H\}$ NMR spectrum of the crude reaction mixture of **2aa**.*

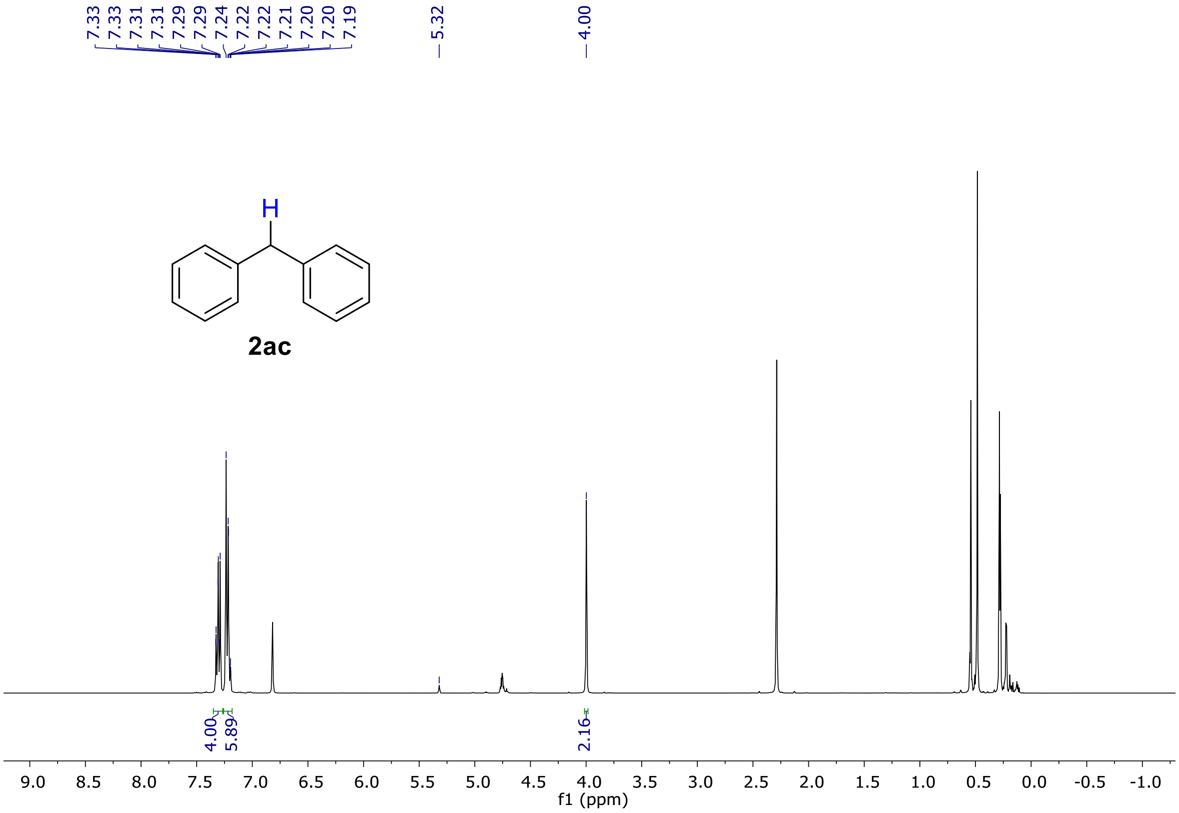

*Figure S73. ¹H NMR spectrum of the crude reaction mixture of **2ac**.*

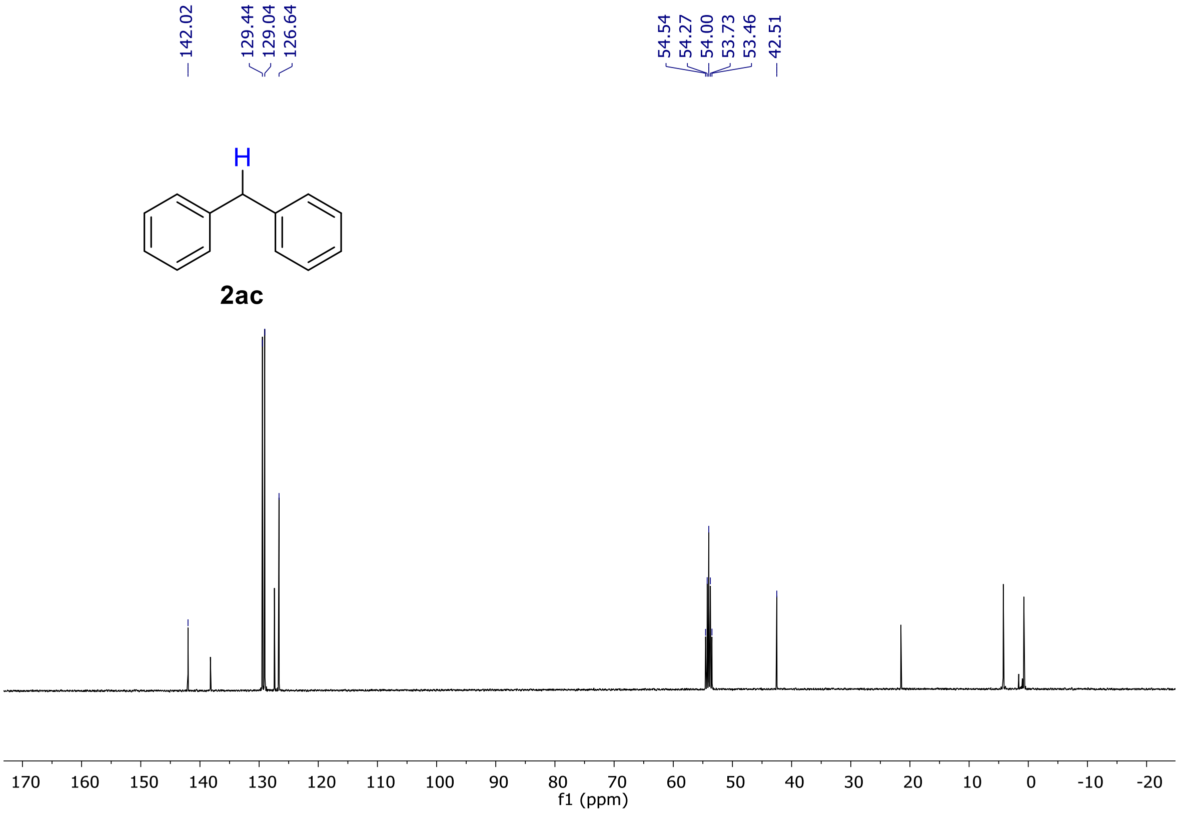

*Figure S74. $^{13}C\{^{1}H\}$ NMR spectrum of the crude reaction mixture of **2ac**.*

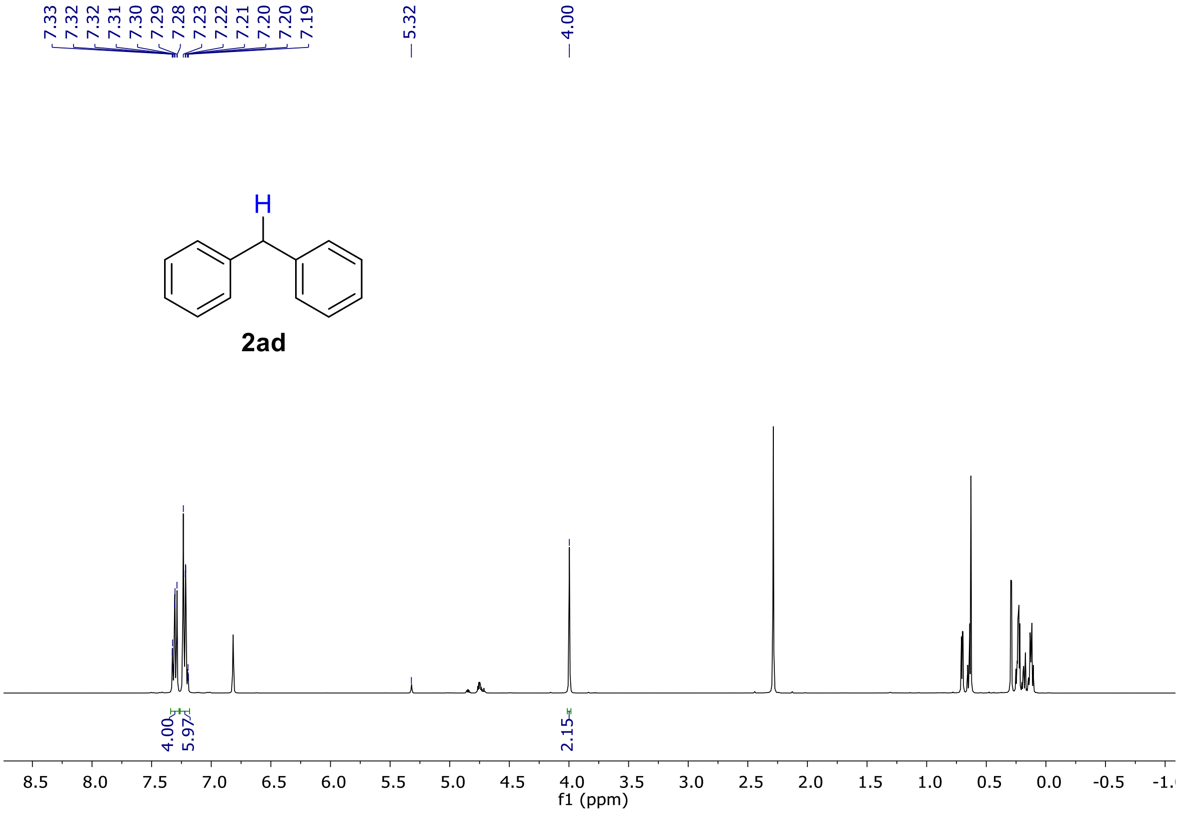

*Figure S75. ^{1}H NMR spectrum of the crude reaction mixture of **2ad**.*

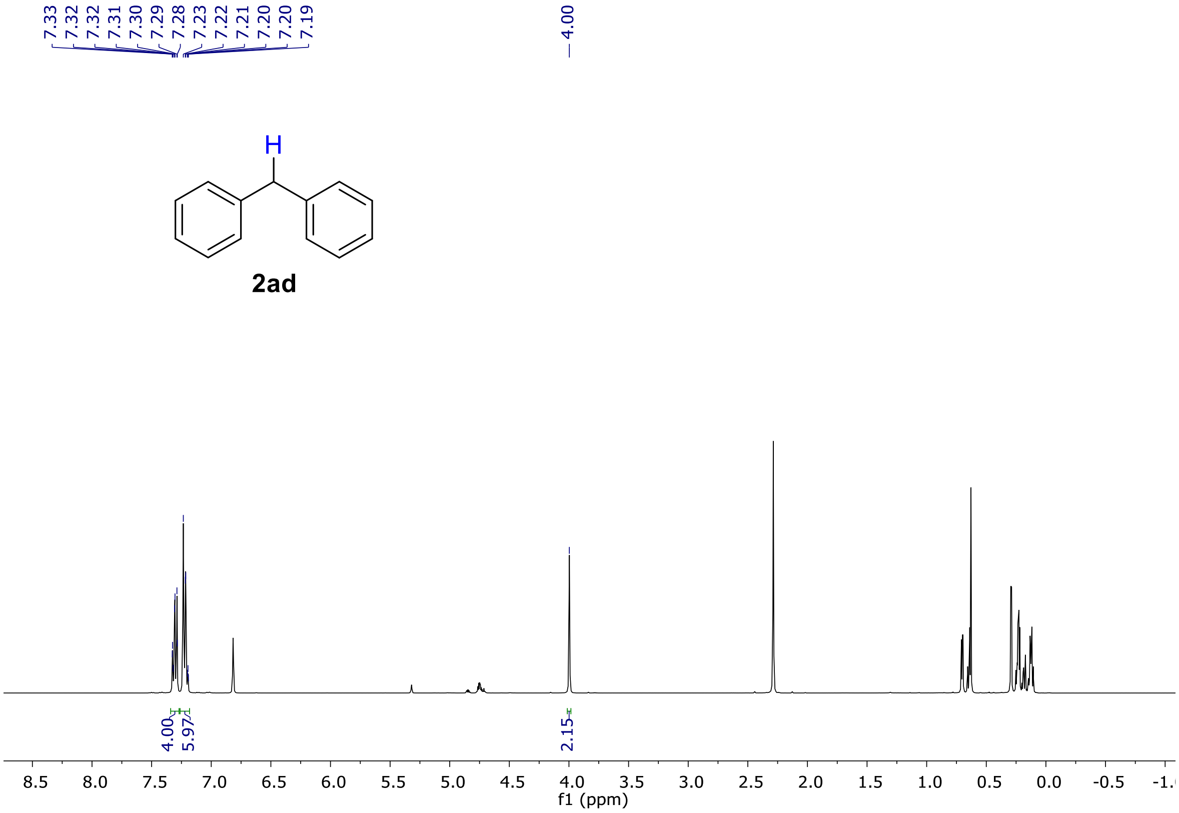

*Figure S76. $^{13}C\{^{1}H\}$ NMR spectrum of the crude reaction mixture of **2ad**.*

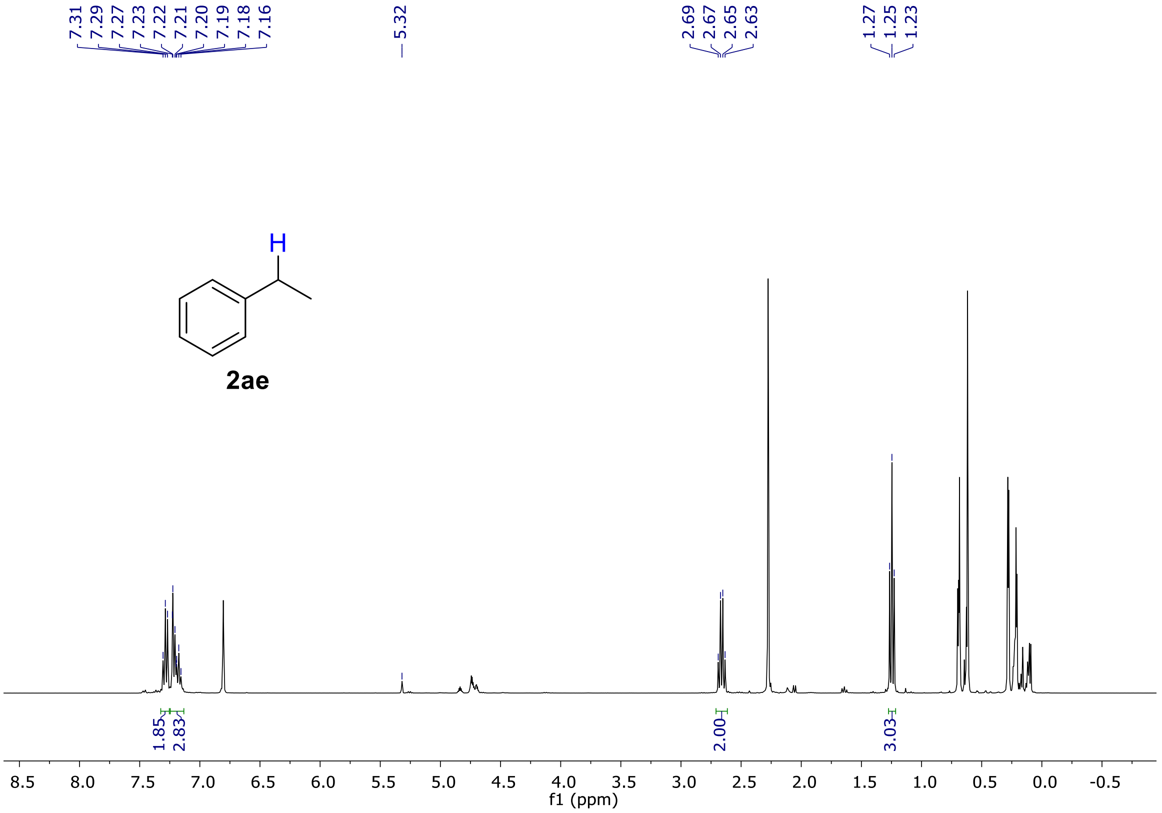

*Figure S77. ^{1}H NMR spectrum of the crude reaction mixture of **2ae**.*

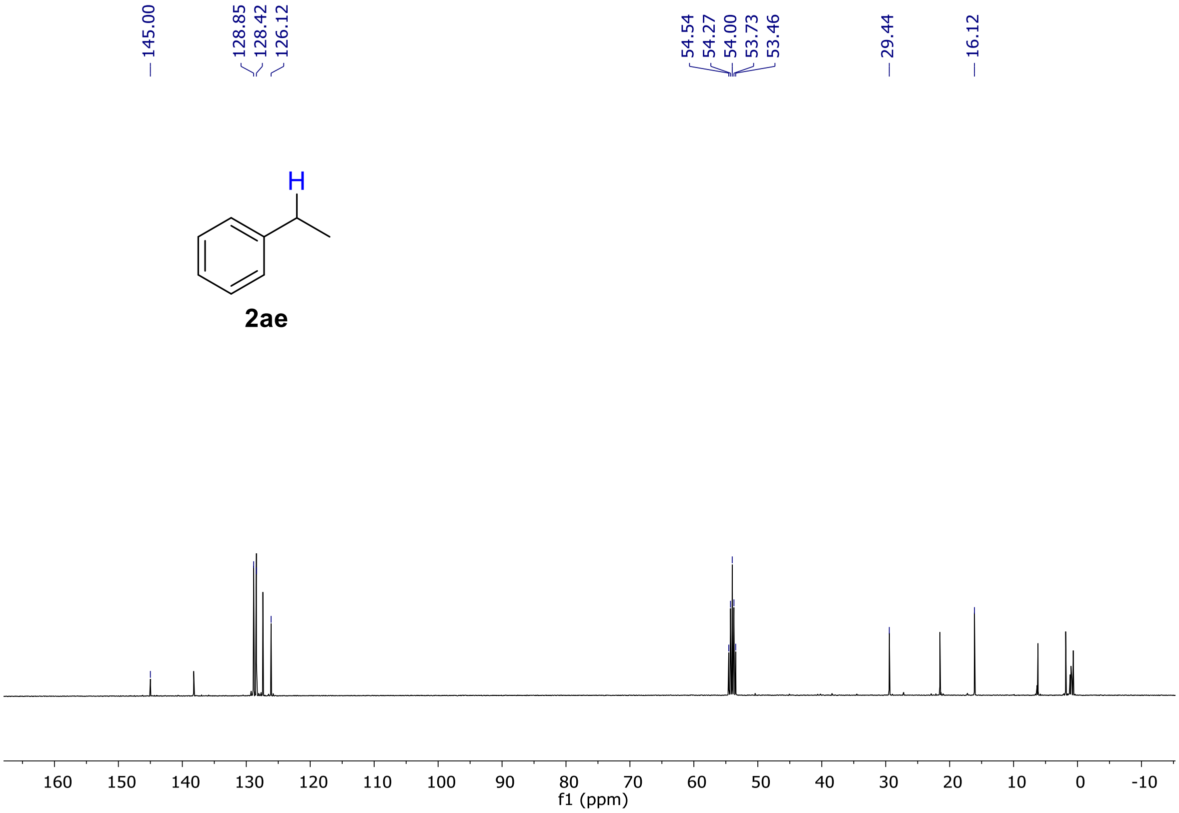

*Figure S78. $^{13}C\{^{1}H\}$ NMR spectrum of the crude reaction mixture of **2ae**.*

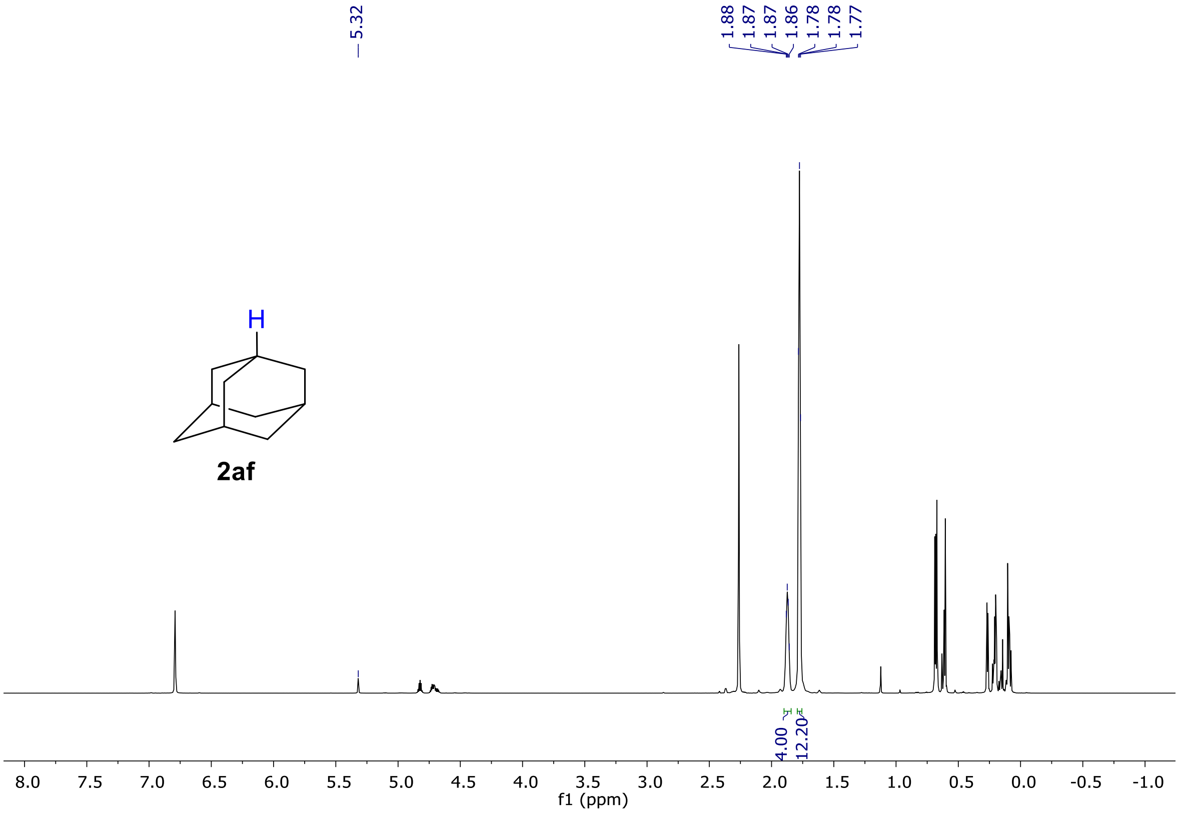

*Figure S79. ^{1}H NMR spectrum of the crude reaction mixture of **2af**.*

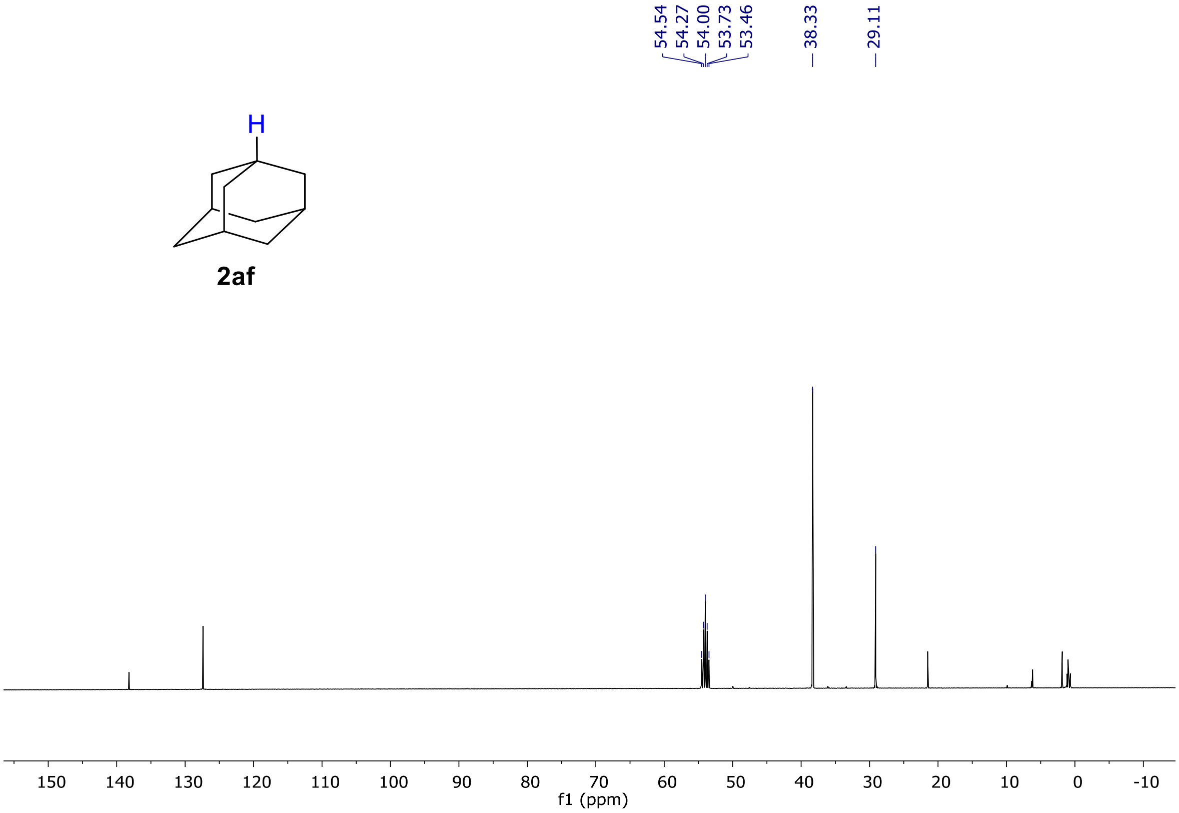

*Figure S80. ^{13}C{^{1}H} NMR spectrum of the crude reaction mixture of **2af**.*

9.4 ^{1}H and ^{13}C NMR Spectra of alkanes obtained by Hydrodehalogenation of alkyl halides 1ab–1ad using Et_3SiH

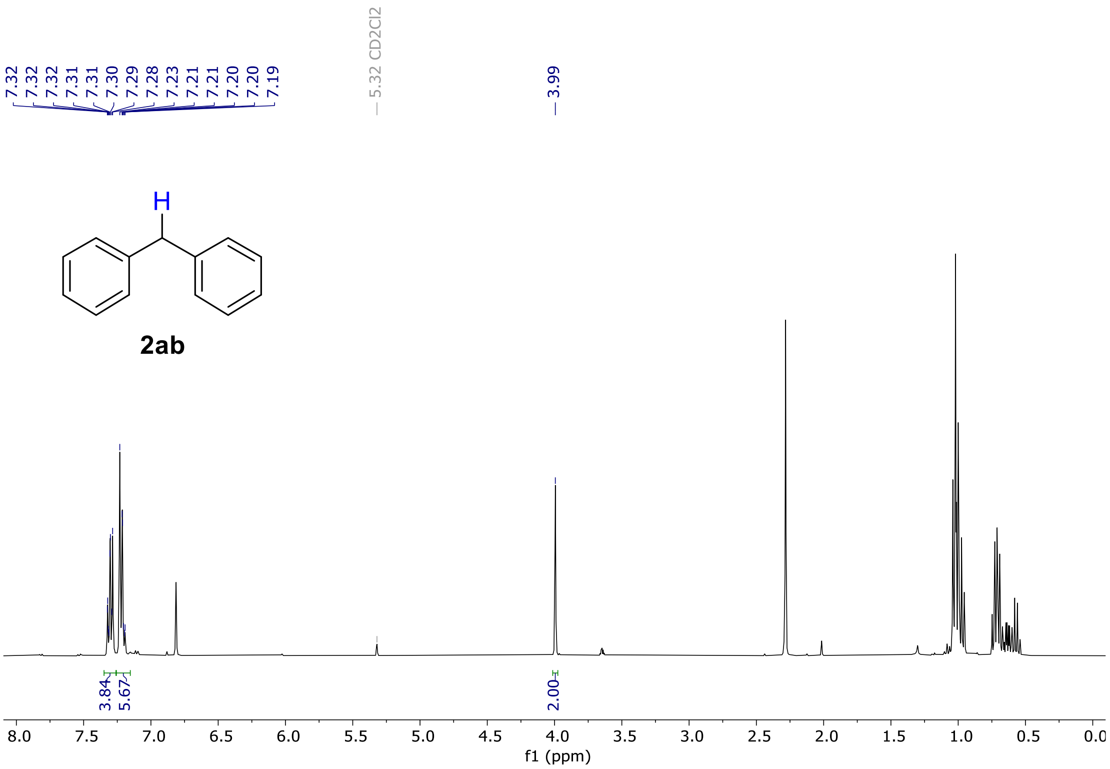

*Figure S81. ^{1}H NMR spectrum of the crude reaction mixture of **2ab**.*

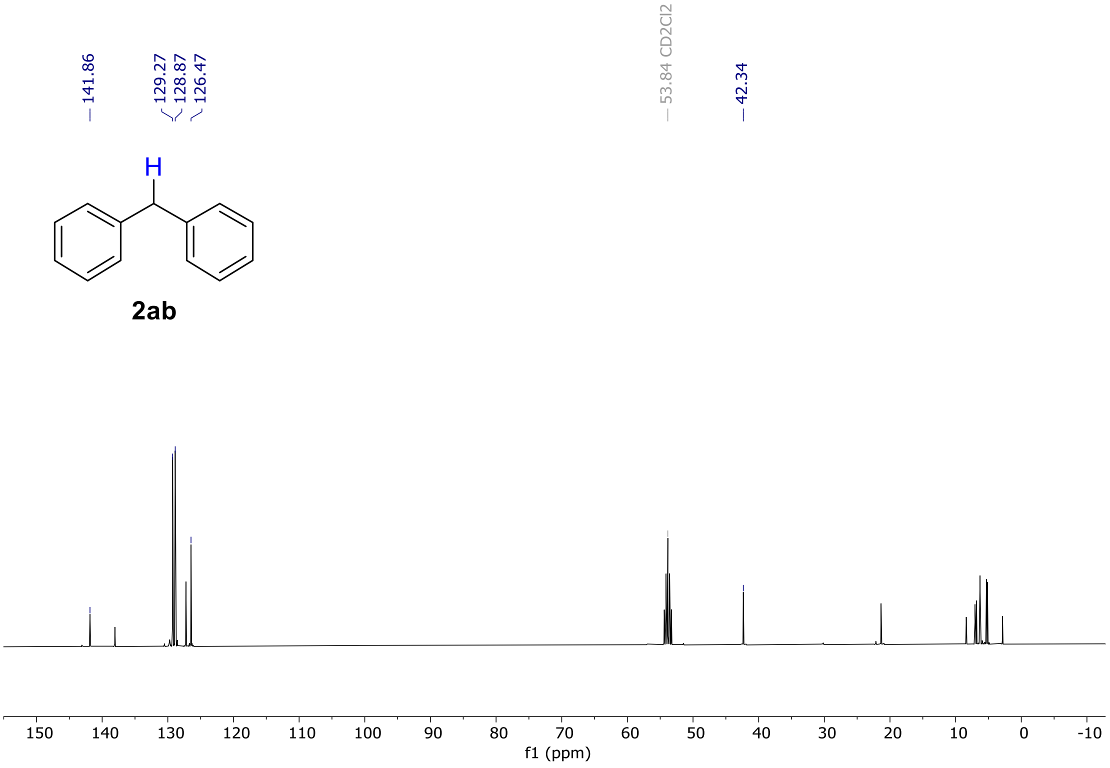

*Figure S82. $^{13}C\{^1H\}$ NMR spectrum of the crude reaction mixture of **2ab**.*

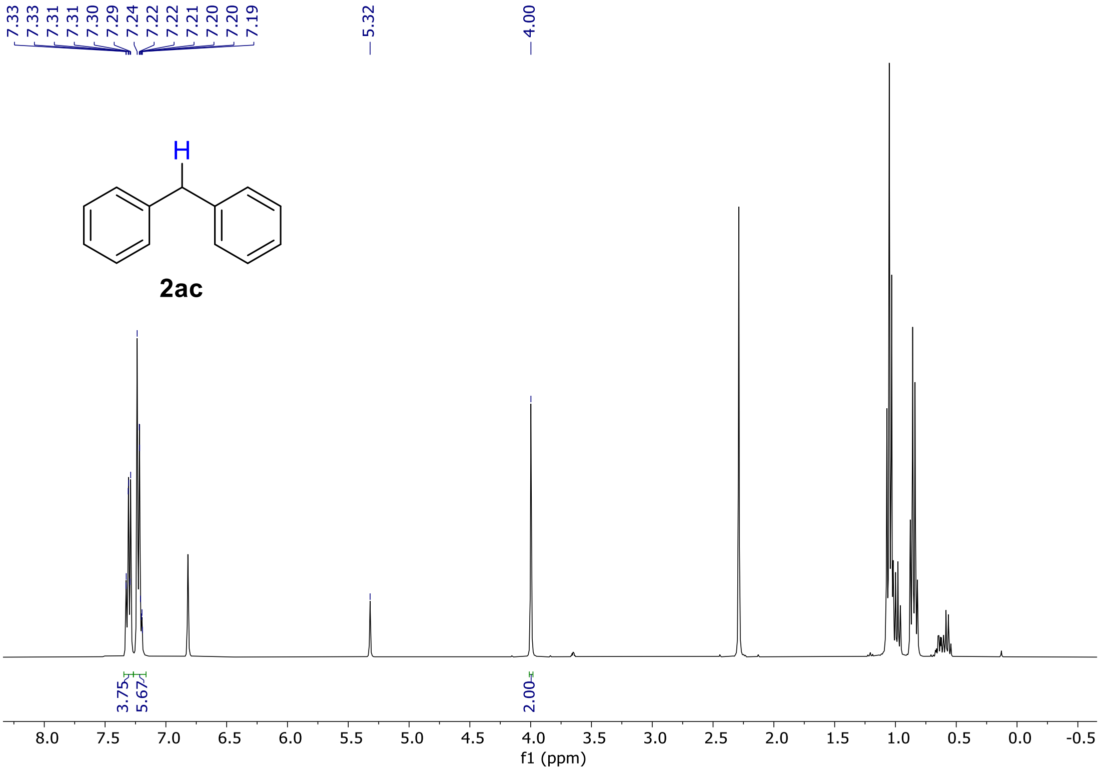

*Figure S83. ^{1}H NMR spectrum of the crude reaction mixture of **2ac**.*

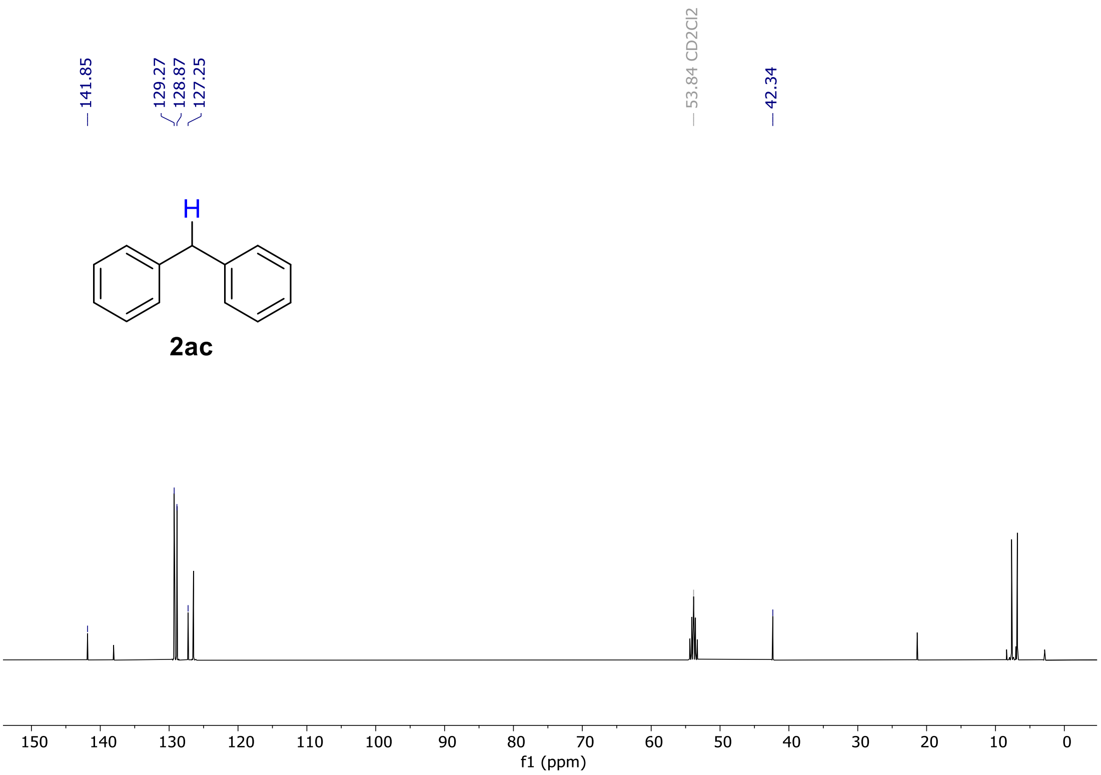

*Figure S84. ^{13}C{^{1}H} NMR spectrum of the crude reaction mixture of **2ac**.*

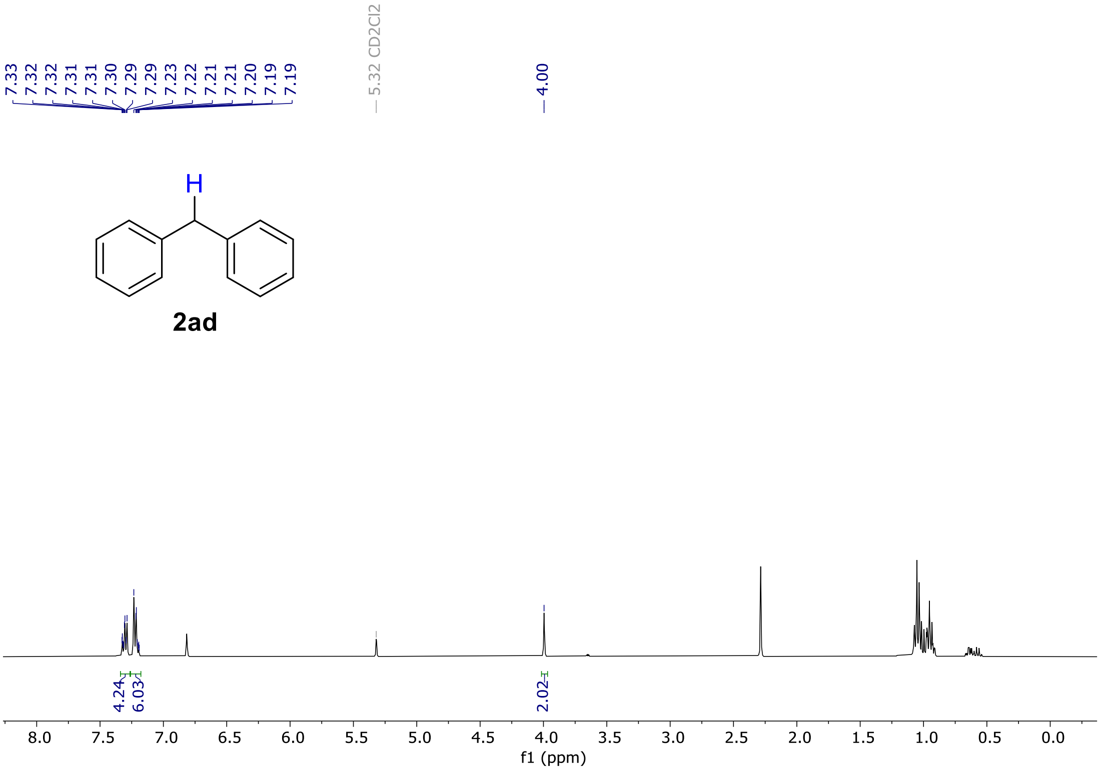

*Figure S85. ^{1}H NMR spectrum of the crude reaction mixture of **2ad**.*

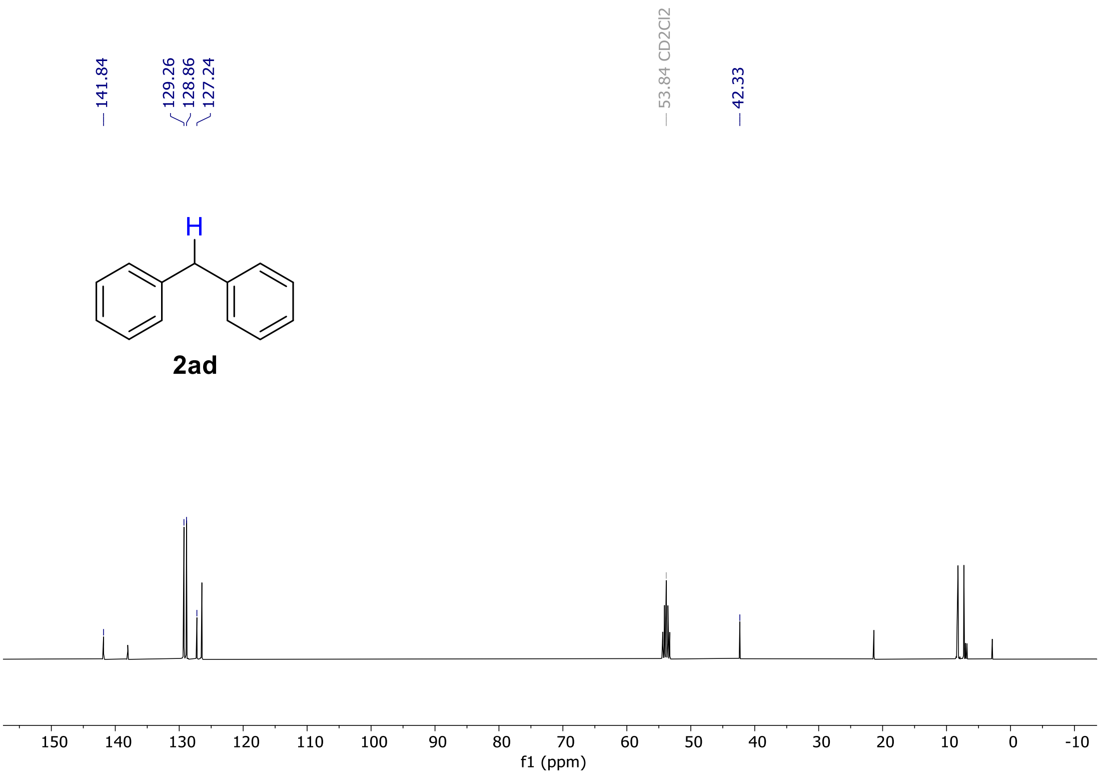

*Figure S86. $^{13}C\{^{1}H\}$ NMR spectrum of the crude reaction mixture of **2ad**.*

9.5 ^{1}H and ^{13}C NMR Spectra of 2aj and 2ak

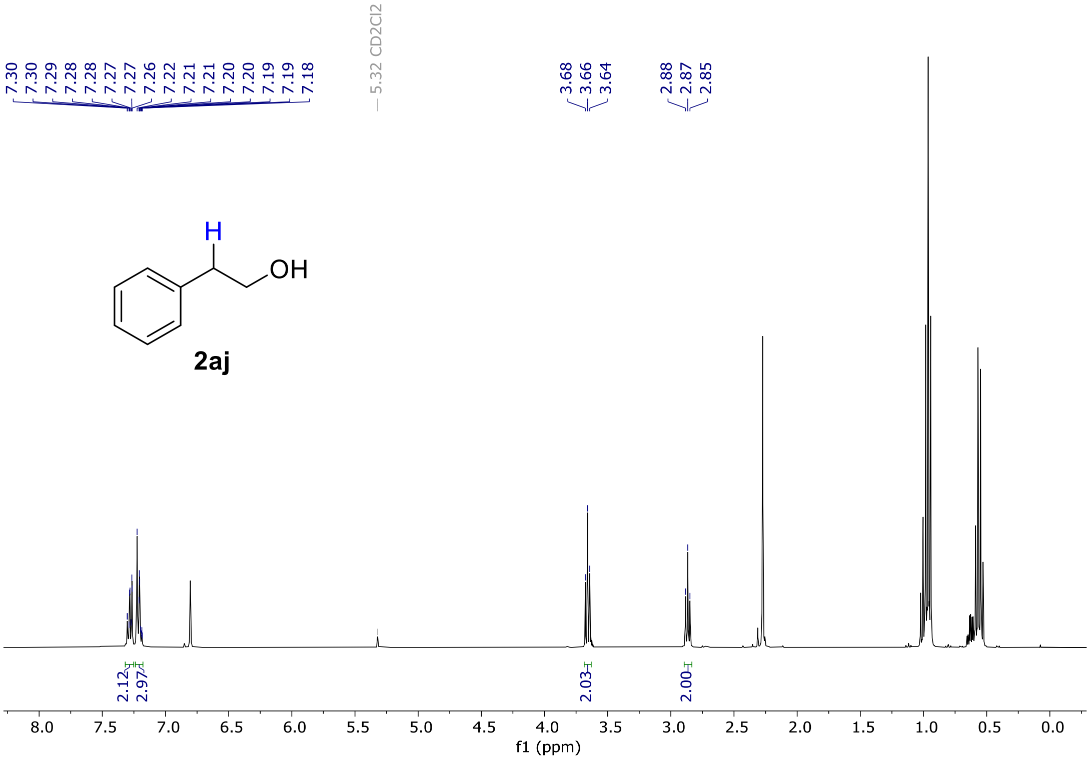

*Figure S87. ^{1}H NMR spectrum of the crude reaction mixture of **2aj**.*

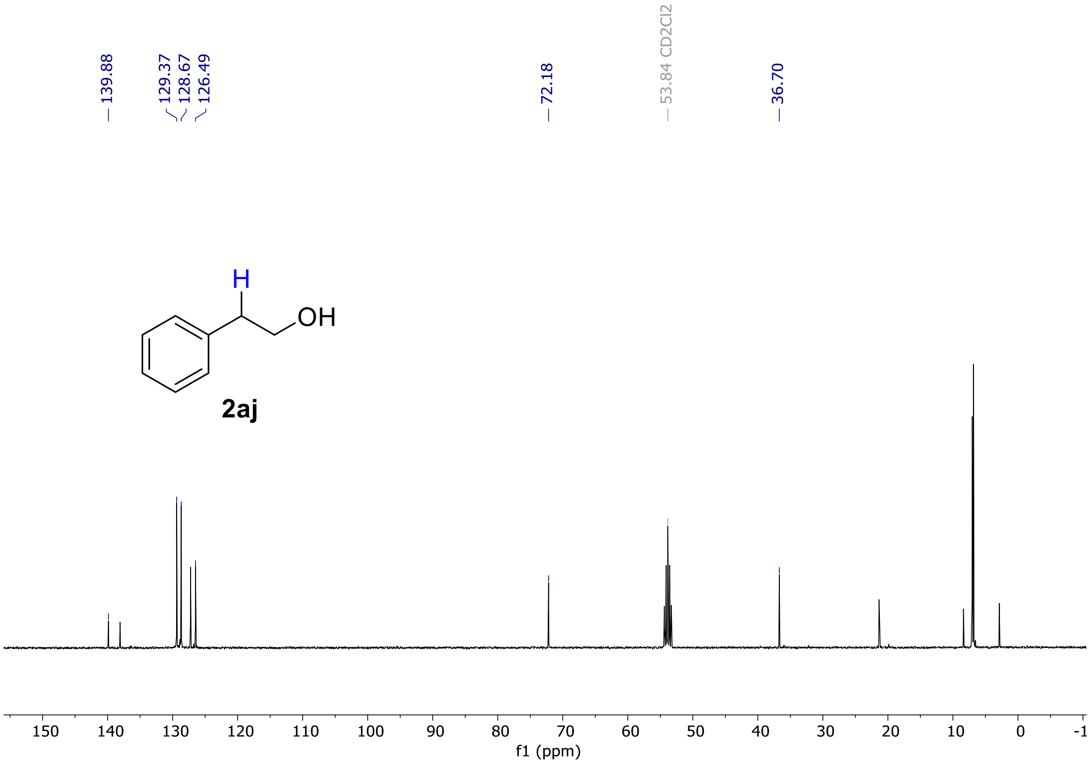

*Figure S88. $^{13}C\{^{1}H\}$ NMR spectrum of the crude reaction mixture of **2aj**.*

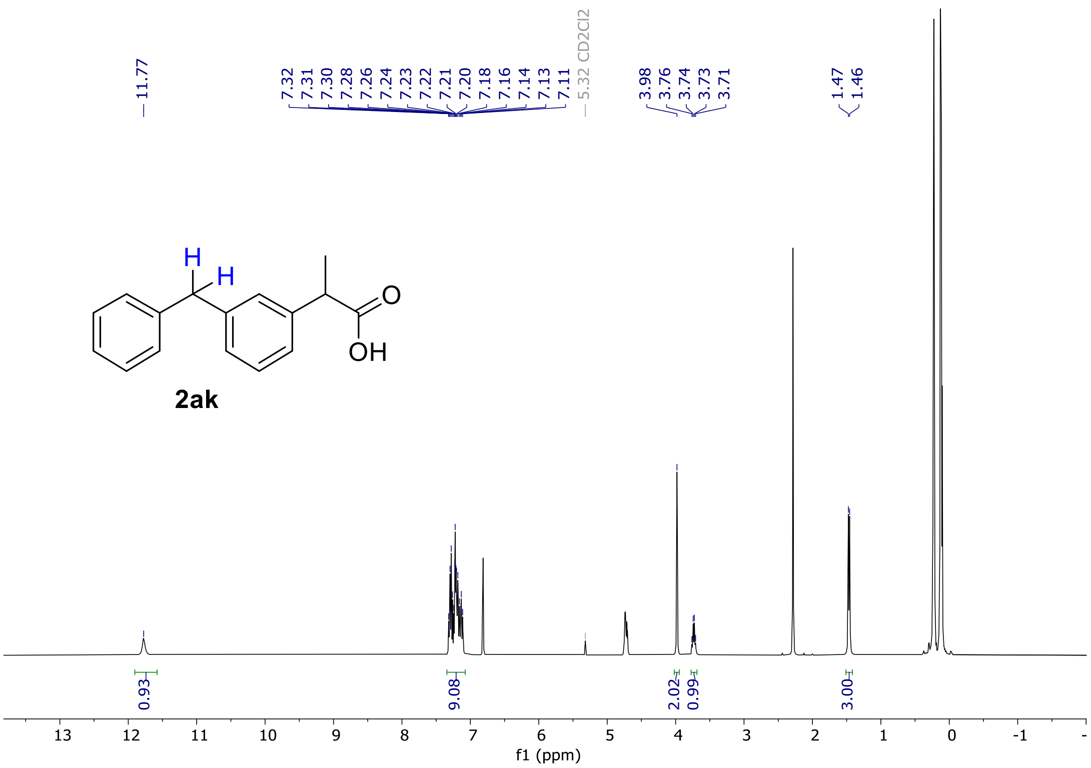

*Figure S89. ^{1}H NMR spectrum of the crude reaction mixture of **2ak**.*

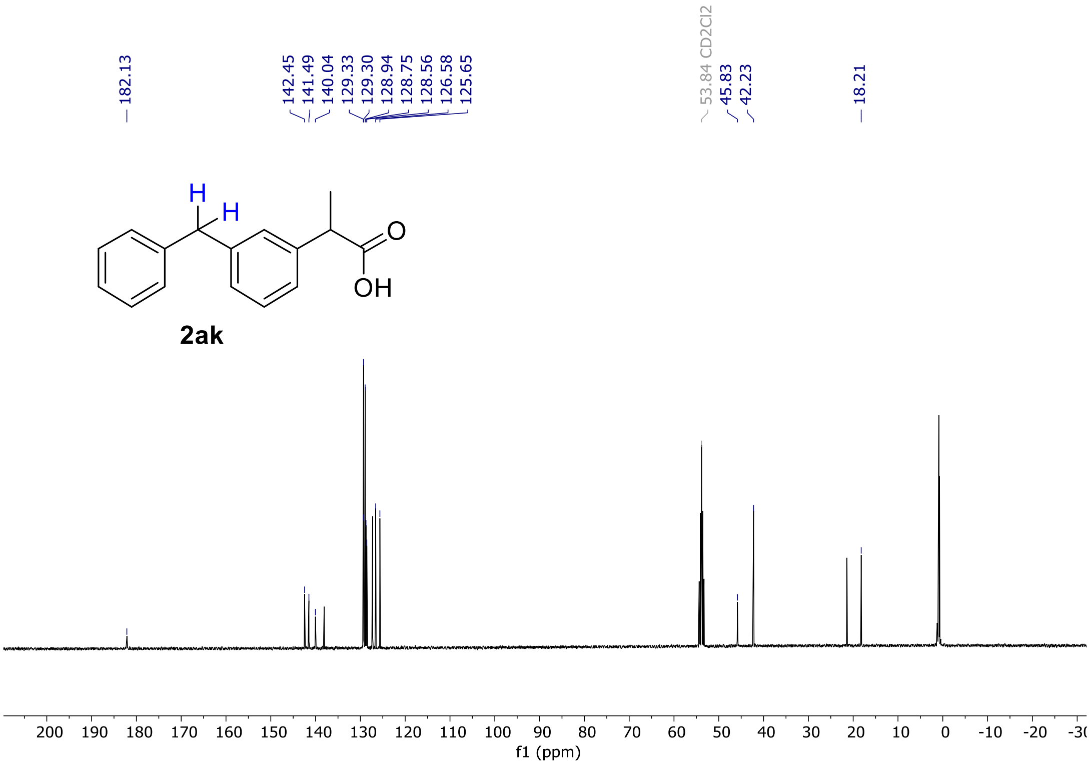

*Figure S90. $^{13}C\{^{1}H\}$ NMR spectrum of the crude reaction mixture of **2ak**.*

9.6 ^{1}H and ^{13}C NMR Spectra of silyl ethers

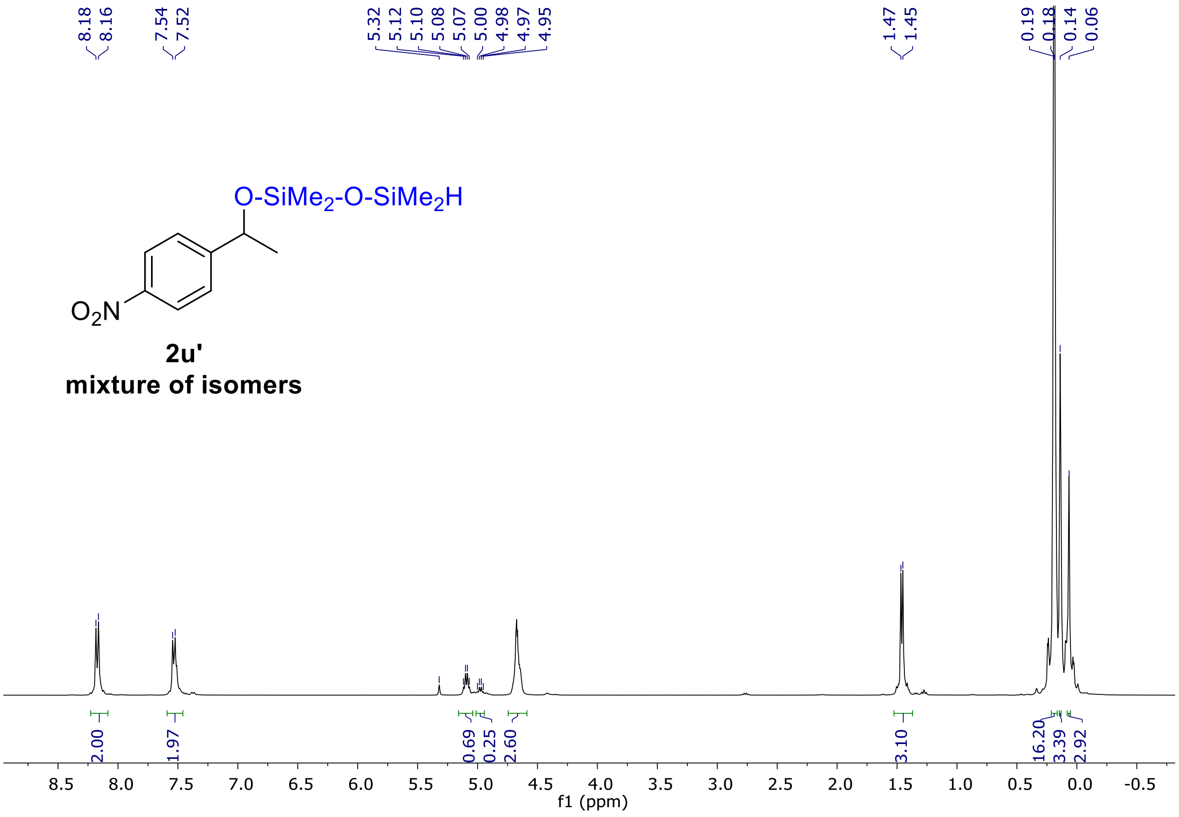

Figure S91. *^{1}H NMR spectrum of the crude reaction mixture of* ***2u'****.*

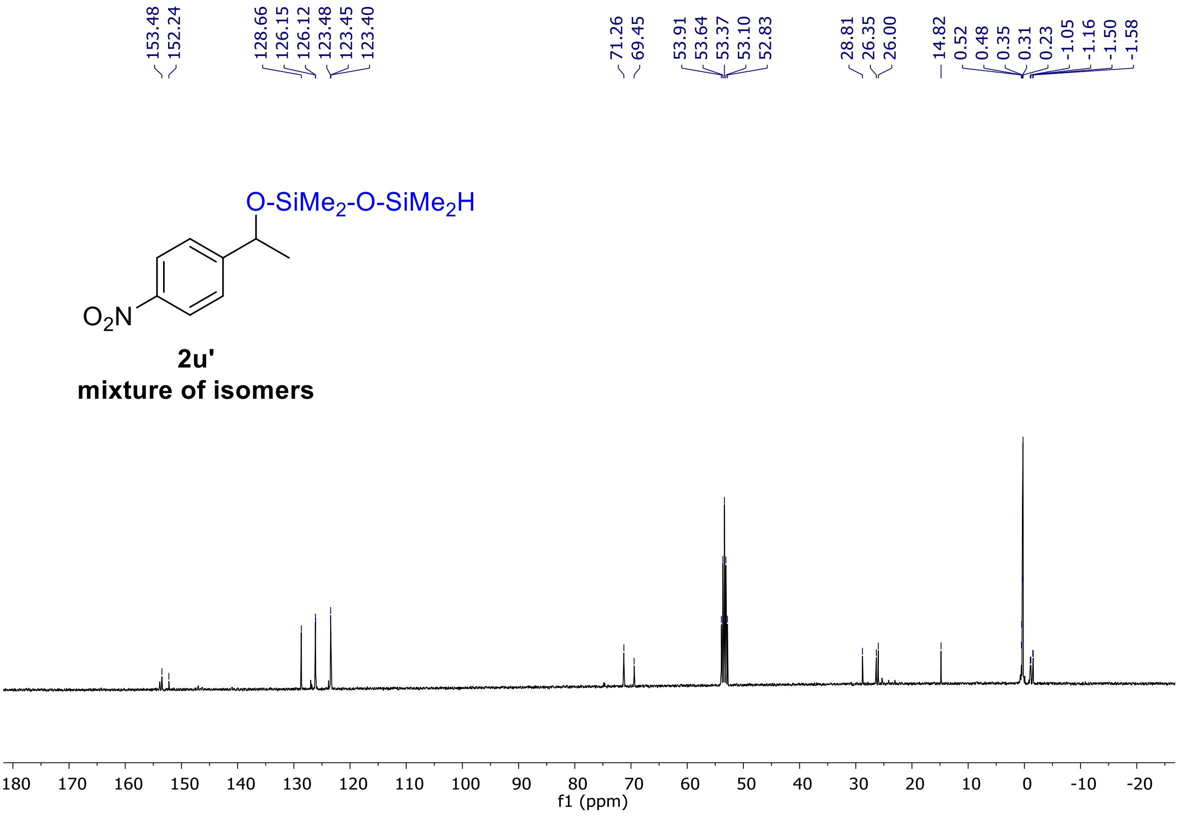

Figure S92. *^{13}C{^{1}H} NMR spectrum of the crude reaction mixture of* ***2u'****.*

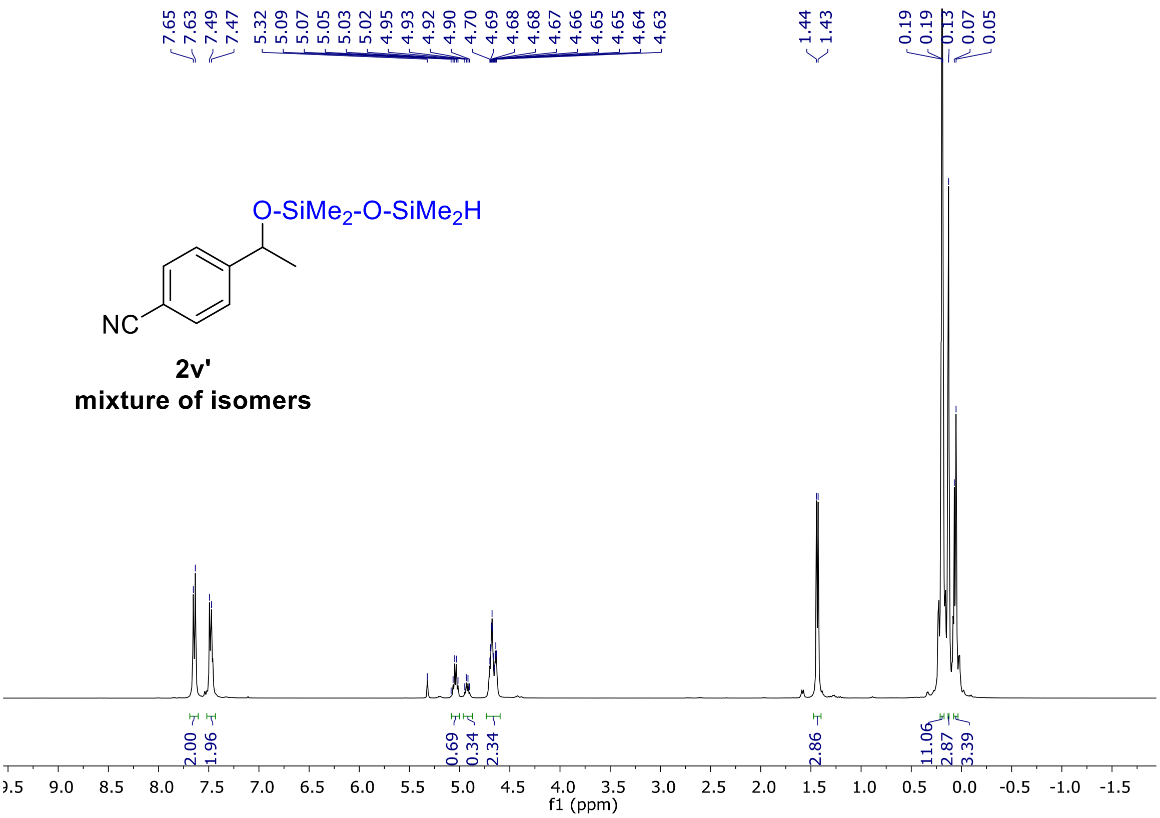

*Figure S93. ¹H NMR spectrum of the crude reaction mixture of **2v′**.*

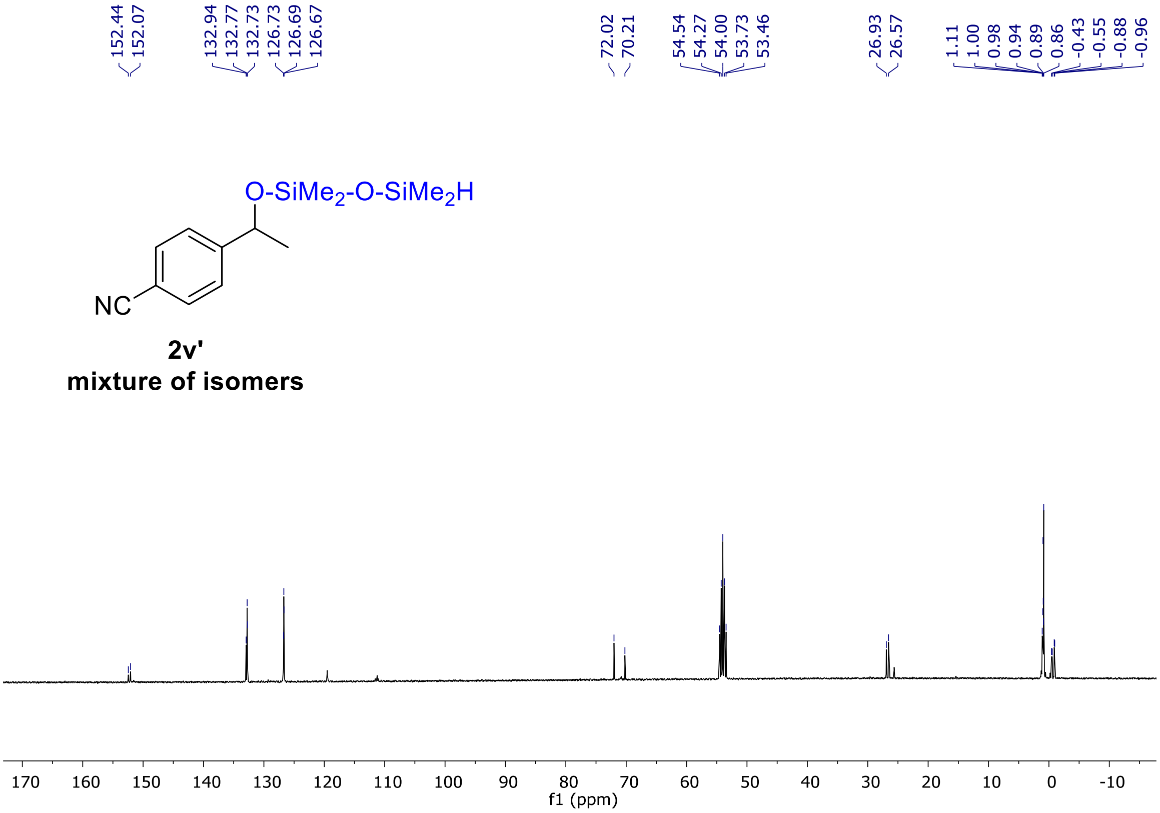

*Figure S94. $^{13}C\{^1H\}$ NMR spectrum of the crude reaction mixture of **2v′**.*

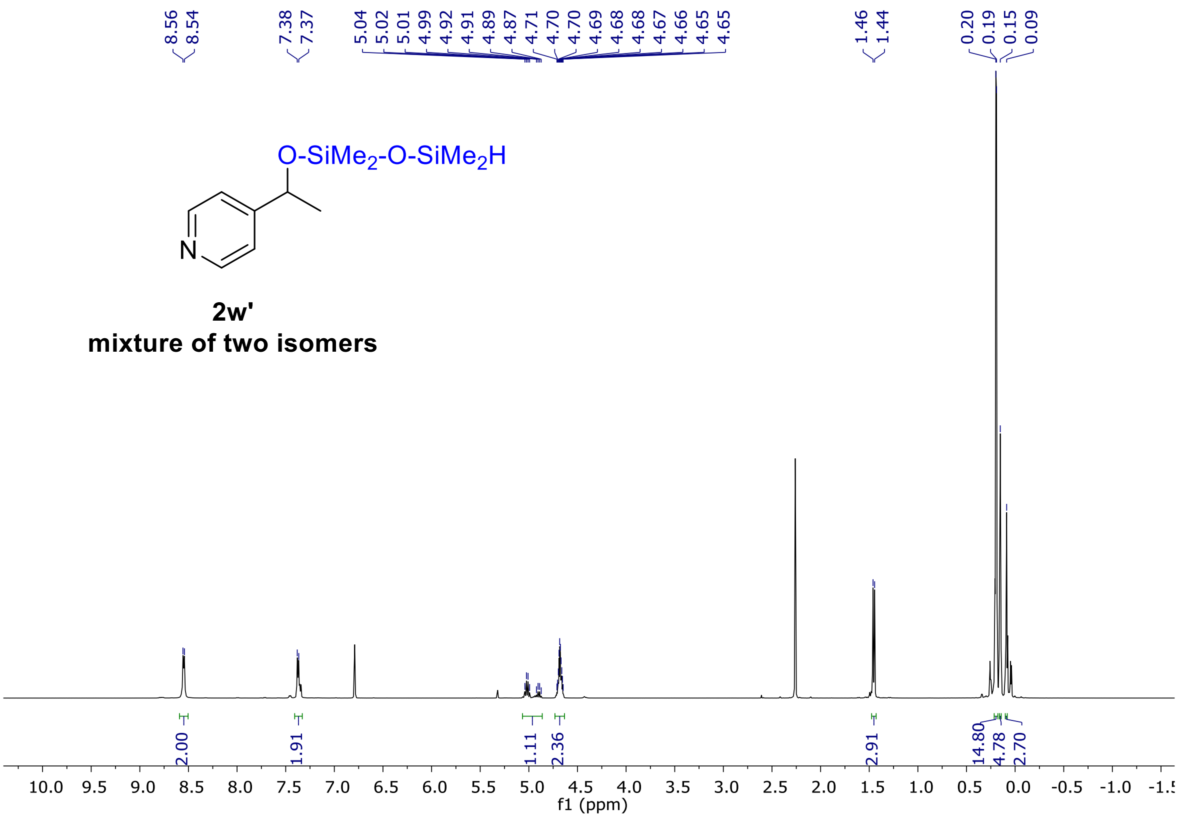

*Figure S95. ^{1}H NMR spectrum of the crude reaction mixture of **2w'**.*

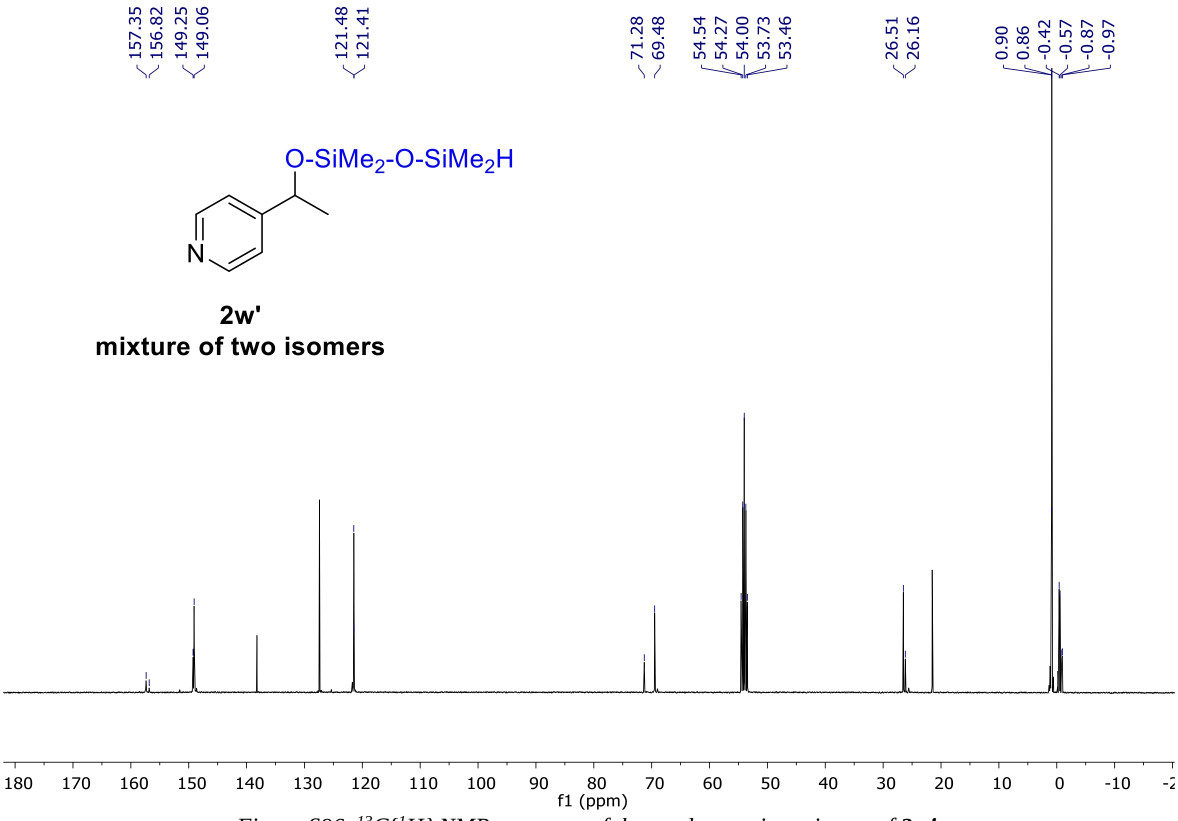

*Figure S96. ^{13}C{^{1}H} NMR spectrum of the crude reaction mixture of **2w'**.*
